# Targeting a portion of central European spider diversity for permanent preservation

**DOI:** 10.3897/BDJ.1.e980

**Published:** 2013-09-16

**Authors:** Klemen Čandek, Matjaž Gregorič, Rok Kostanjšek, Holger Frick, Christian Kropf, Matjaž Kuntner

**Affiliations:** † Institute of Biology, Scientific Research Centre, Slovenian Academy of Sciences and Arts, Ljubljana, Slovenia; ‡ Department of Biology, Biotechnical faculty, University of Ljubljana, Ljubljana, Slovenia; § National Collection of Natural History, Office of Environment, Vaduz, Liechtenstein; | Department of Invertebrates, Natural History Museum, Bern, Switzerland; ¶ National Museum of Natural History, Smithsonian Institution, Washington, DC, United States of America

**Keywords:** Conservation, DNA barcoding, cryobank, biorepository, faunistics

## Abstract

Given the limited success of past and current conservation efforts, an alternative approach is to preserve tissues and genomes of targeted organisms in cryobanks to make them accessible for future generations. Our pilot preservation project aimed to obtain, expertly identify, and permanently preserve a quarter of the known spider species diversity shared between Slovenia and Switzerland, estimated at 275 species. We here report on the faunistic part of this project, which resulted in 324 species (227 in Slovenia, 143 in Switzerland) for which identification was reasonably established. This material is now preserved in cryobanks, is being processed for DNA barcoding, and is available for genomic studies.

## Introduction

Today, the importance of understanding and conserving biodiversity is undisputed. However, climate changes, urbanization, deforestation and pollution all critically affect most of the planet's ecosystems, which consequently are changing too rapidly for organisms to adapt to (Dornelas et al. 2013). Biodiversity exploration, preservation and conservation are thus among the main challenges for humanity, yet our current efforts seem not to be effective enough (Hoban and Vernesi 2012). Because of that, several new approaches for biodiversity conservation recently emerged, among which is cryo-preservation. As an example, Smithsonian Institution recently launched the Global Genome Initiative (http://www.mnh.si.edu/ggi/), whose goal is precisely to preserve tissues and genomes of many targeted organisms to make these materials accessible for future generations. As a pilot preservation project funded by the Swiss Contribution to the enlarged EU we partnered with the Global Genome Initiative to conduct a study that aimed to permanently preserve a large proportion of European spider fauna by deep-freezing their tissues and DNA. With over 43 thousand known species (http://research.amnh.org/iz/spiders/catalog/INTRO1.html), the hyperdiverse order Araneae ranks seventh among animal orders (Coddington and Levi 1991). As obligate predators, spiders also play prominent ecological roles in all terrestrial ecosystems, and are important biocontrol agents (Foelix 2010). Biorepositoring a portion of a known spider fauna, thus, may be an important step in conservation. However, because species identification in spiders is not straightforward, the goal here was to produce expertly identified specimens for preservation, for genomic DNA extraction and for DNA barcoding (Hebert et al. 2003). We targeted a quarter of the known spider diversity shared between Slovenia and Switzerland, which estimated from the available checklists (Maurer and Hänggi 1990, http://www.arages.de/checklist.html#2004_Araneae, own unpublished data for Slovenia) may number roughly 275 species. To do that, we conducted field work at over 100 localities throughout Slovenia and Switzerland. Here, we report on the faunistic data from this project, resulting in well over the targeted numbers of expertly identified taxa.

## Materials and methods

**Figure 1.**
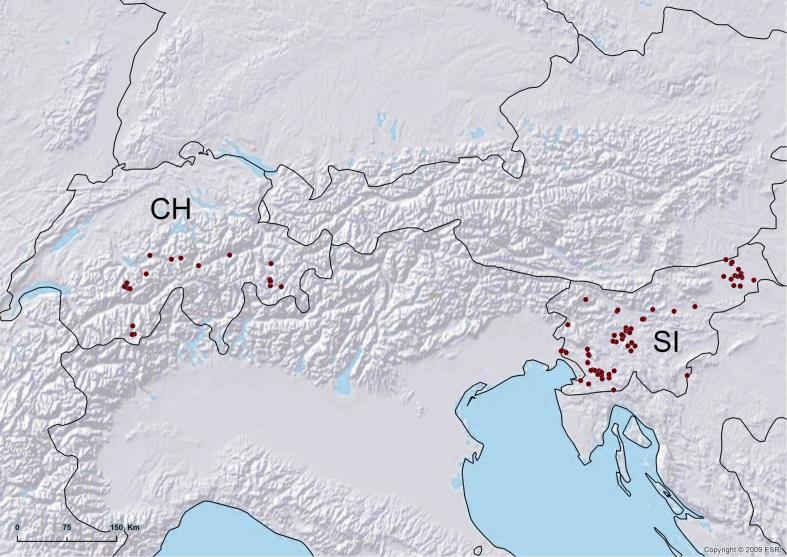
A map of central Europe with sampling localities indicated. Legend: SI = Slovenia; CH = Switzerland

In 2010-2012, we collected spiders at 74 and 33 localities in Slovenia and Switzerland, respectively (Fig. 1). In order to increase the number of targeted spider species, we sampled in a wide variety of habitats ranging from mountain regions, lowland grasslands and forests, to coastal regions and caves. To sample in diverse microhabitats, we used several sampling methods, i.e. hand-collecting, beating vegetation, using sweep nets and sifting leaf litter. We also sampled using a gasoline powered leaf blower with a sock-like filter made out of rough textile to intercept material. Captured spiders were immediately fixed in absolute ethanol and samples were kept in a cooler or refrigerator. In the laboratory, we discarded all juveniles as is standard in quantitative sampling (Coddington et al. 1996, Coddington et al. 2009, Scharff et al. 2003). We expertly identified adults to species level where possible. Identifications were mainly done using the online European spider key (www.araneae.unibe.ch), but we also consulted primary literature as needed. A representative subsample of all identified species was set aside for DNA extraction for DNA barcoding purposes (these results will be reported elsewhere). The voucher specimens resulting from this study will be deposited in the collections of the National Museum of Natural History, Smithsonian Institution, USA, and in Naturhistorisches Museum Bern, Switzerland, but the bulk of the material remains in the study collection of the EZ lab at the Institute of Biology, Scientific Research Centre, Slovenian Academy of Sciences and Arts, Ljubljana, Slovenia (http://ezlab.zrc-sazu.si/).

## Checklists

### Checklist

#### Agelenidae

C. L. Koch, 1837

#### Agelena labyrinthica

(Clerck, 1757)

##### Materials

- **Type status:** Other material. **Occurrence:** recordedBy: Kostanjšek, RTŠB 2011; sex: 1 female; **Location:** locationID: SI01; country: Slovenia; locality: Biš; minimumElevationInMeters: 225; maximumElevationInMeters: 225; decimalLatitude: 46.5374; decimalLongitude: 15.8963; **Event:** eventDate: 2011-07-22; habitat: forest
- **Type status:** Other material. **Occurrence:** recordedBy: Čandek; sex: 2 females, 1 male; **Location:** locationID: SI61; country: Slovenia; locality: Sekirišče; minimumElevationInMeters: 750; maximumElevationInMeters: 750; decimalLatitude: 45.8631; decimalLongitude: 14.5367; **Event:** eventDate: 2011-06-23/2012-06-21; habitat: house, grassland, overgrowth

#### Allagelena gracilens

(C. L. Koch, 1841)

##### Materials

- **Type status:** Other material. **Occurrence:** recordedBy: Kostanjšek, RTŠB 2011; sex: 1 male; **Location:** locationID: SI03; country: Slovenia; locality: Cerkvenjak; minimumElevationInMeters: 310; maximumElevationInMeters: 310; decimalLatitude: 46.5694; decimalLongitude: 15.9481; **Event:** eventDate: 2011-07-22; habitat: grassland with bushes
- **Type status:** Other material. **Occurrence:** recordedBy: Kostanjšek, RTŠB 2012; sex: 1 male; **Location:** locationID: SI72; country: Slovenia; locality: Dolnja Košana; minimumElevationInMeters: 415; maximumElevationInMeters: 415; decimalLatitude: 45.6579; decimalLongitude: 14.1384; **Event:** eventDate: 2012-07-21; habitat: rastlinje ob potoku

#### Coelotes terrestris

(Wider, 1834)

##### Materials

- **Type status:** Other material. **Occurrence:** recordedBy: Kostanjšek, RTŠB 2011; sex: 1 male; **Location:** locationID: SI02; country: Slovenia; locality: Hrastovec; minimumElevationInMeters: 270; maximumElevationInMeters: 270; decimalLatitude: 46.5613; decimalLongitude: 15.7953; **Event:** eventDate: 2011-07-22; habitat: forest

#### Histopona torpida

(C. L. Koch, 1837)

##### Materials

- **Type status:** Other material. **Occurrence:** recordedBy: Kostanjšek, RTŠB 2011; sex: 1 female; **Location:** locationID: SI02; country: Slovenia; locality: Hrastovec; minimumElevationInMeters: 270; maximumElevationInMeters: 270; decimalLatitude: 46.5613; decimalLongitude: 15.7953; **Event:** eventDate: 2011-07-22; habitat: forest
- **Type status:** Other material. **Occurrence:** recordedBy: Čandek; sex: 1 female; **Location:** locationID: SI29; country: Slovenia; locality: Gradišče pri Lukovici, Gradiško jezero; minimumElevationInMeters: 360; maximumElevationInMeters: 360; decimalLatitude: 46.1626; decimalLongitude: 14.7127; **Event:** eventDate: 2011-10-06; habitat: lake edge
- **Type status:** Other material. **Occurrence:** recordedBy: Kuntner, Gregorič, Lokovšek; sex: 1 female; **Location:** locationID: SI39; country: Slovenia; locality: Primostek; minimumElevationInMeters: 157; maximumElevationInMeters: 157; decimalLatitude: 45.6299; decimalLongitude: 15.2997; **Event:** eventDate: 2010-08-24; habitat: grassland
- **Type status:** Other material. **Occurrence:** recordedBy: Kuntner, Lokovšek; sex: 1 female; **Location:** locationID: SI40; country: Slovenia; locality: Slavnik; minimumElevationInMeters: 816; maximumElevationInMeters: 816; decimalLatitude: 45.5499; decimalLongitude: 13.9619; **Event:** eventDate: 2010-08-26; habitat: grassland and forest
- **Type status:** Other material. **Occurrence:** recordedBy: Čandek; sex: 2 females; **Location:** locationID: SI59; country: Slovenia; locality: Budanje; minimumElevationInMeters: 305; maximumElevationInMeters: 305; decimalLatitude: 45.8797; decimalLongitude: 13.9468; **Event:** eventDate: 2011-05-07; habitat: forest

#### Inermocoelotes anoplus

(Kulczyn'ski, 1897)

##### Materials

- **Type status:** Other material. **Occurrence:** recordedBy: Kuntner, Lokovšek; sex: 1 female, 2 males; **Location:** locationID: SI40; country: Slovenia; locality: Slavnik; minimumElevationInMeters: 816; maximumElevationInMeters: 816; decimalLatitude: 45.5499; decimalLongitude: 13.9619; **Event:** eventDate: 2010-08-26; habitat: grassland and forest
- **Type status:** Other material. **Occurrence:** recordedBy: Čandek; sex: 2 females; **Location:** locationID: SI59; country: Slovenia; locality: Budanje; minimumElevationInMeters: 305; maximumElevationInMeters: 305; decimalLatitude: 45.8797; decimalLongitude: 13.9468; **Event:** eventDate: 2011-05-07; habitat: forest

#### Malthonica silvestris

(L. Koch, 1872)

##### Materials

- **Type status:** Other material. **Occurrence:** recordedBy: Kostanjšek, RTŠB 2011; sex: 1 female; **Location:** locationID: SI02; country: Slovenia; locality: Hrastovec; minimumElevationInMeters: 270; maximumElevationInMeters: 270; decimalLatitude: 46.5613; decimalLongitude: 15.7953; **Event:** eventDate: 2011-07-22; habitat: forest
- **Type status:** Other material. **Occurrence:** recordedBy: Čandek; sex: 2 females; **Location:** locationID: SI29; country: Slovenia; locality: Gradišče pri Lukovici, Gradiško jezero; minimumElevationInMeters: 360; maximumElevationInMeters: 360; decimalLatitude: 46.1626; decimalLongitude: 14.7127; **Event:** eventDate: 2011-10-06; habitat: lake edge

#### Tegenaria atrica

C. L. Koch, 1843

##### Materials

- **Type status:** Other material. **Occurrence:** recordedBy: Kuntner, Gregorič, Čandek; sex: 1 female; **Location:** locationID: CH09; country: Switzerland; locality: Pennine Alps, Mattertal; minimumElevationInMeters: 1447; maximumElevationInMeters: 1447; decimalLatitude: 46.0976; decimalLongitude: 7.7789; **Event:** eventDate: 2011-07-08; habitat: forest and meadow near river
- **Type status:** Other material. **Occurrence:** recordedBy: Kuntner; sex: 2 males; **Location:** locationID: SI45; country: Slovenia; locality: Vrh nad Želimljami, Škofljica; minimumElevationInMeters: 546; maximumElevationInMeters: 546; decimalLatitude: 45.9091; decimalLongitude: 14.5934; **Event:** eventDate: 2010-09-12; habitat: house and surroundings
- **Type status:** Other material. **Occurrence:** recordedBy: Aljančič; sex: 1 female; **Location:** locationID: SI62; country: Slovenia; locality: Kranj; minimumElevationInMeters: 394; maximumElevationInMeters: 394; decimalLatitude: 46.2482; decimalLongitude: 14.3591; **Event:** eventDate: 2011-03-30; habitat: house and surroundings

#### Amaurobiidae

Thorell, 1870

#### Amaurobius erberi

(Keyserling, 1863)

##### Materials

- **Type status:** Other material. **Occurrence:** recordedBy: Kuntner, Gregorič, Čandek, Kralj-Fišer, Cheng; sex: 1 female; **Location:** locationID: SI41; country: Slovenia; locality: Socerb, Osp; minimumElevationInMeters: 116; maximumElevationInMeters: 116; decimalLatitude: 45.5819; decimalLongitude: 13.8558; **Event:** eventDate: 2012-06-07; habitat: trail from Socerb to Osp
- **Type status:** Other material. **Occurrence:** recordedBy: Gregorič, Čandek, Kralj-Fišer; sex: 1 female; **Location:** locationID: SI56; country: Slovenia; locality: Dinaric Karst, Novelo; minimumElevationInMeters: 358; maximumElevationInMeters: 359; decimalLatitude: 45.8533; decimalLongitude: 13.6552; **Event:** eventDate: 2011-04-04/05-10; habitat: overgrowth

#### Amaurobius fenestralis

(Ström, 1768)

##### Materials

- **Type status:** Other material. **Occurrence:** recordedBy: Kuntner, Gregorič, Čandek; sex: 1 female; **Location:** locationID: CH09; country: Switzerland; locality: Pennine Alps, Mattertal; minimumElevationInMeters: 1447; maximumElevationInMeters: 1447; decimalLatitude: 46.0976; decimalLongitude: 7.7789; **Event:** eventDate: 2011-07-08; habitat: forest and meadow near river

#### Amaurobius ferox

(Walckenaer, 1830)

##### Materials

- **Type status:** Other material. **Occurrence:** recordedBy: Kuntner, Čandek; sex: 1 female; **Location:** locationID: SI50; country: Slovenia; locality: Sp. Prapreče; minimumElevationInMeters: 351; maximumElevationInMeters: 351; decimalLatitude: 46.1620; decimalLongitude: 14.6933; **Event:** eventDate: 2010-08-03/2012-05-28; habitat: house and surroundings

#### Amaurobius jugorum

L. Koch, 1868

##### Materials

- **Type status:** Other material. **Occurrence:** recordedBy: Kuntner, Lokovšek; sex: 1 female; **Location:** locationID: SI40; country: Slovenia; locality: Slavnik; minimumElevationInMeters: 816; maximumElevationInMeters: 816; decimalLatitude: 45.5499; decimalLongitude: 13.9619; **Event:** eventDate: 2010-08-26; habitat: grassland and forest
- **Type status:** Other material. **Occurrence:** recordedBy: Kostanjšek, RTŠB 2012; sex: 1 female; **Location:** locationID: SI75; country: Slovenia; locality: Buje; minimumElevationInMeters: 470; maximumElevationInMeters: 470; decimalLatitude: 45.6543; decimalLongitude: 14.0924; **Event:** eventDate: 2012-07-20; habitat: forest

#### Anyphaenidae

Bertkau, 1878

#### Anyphaena accentuata

(Walckenaer, 1802)

##### Materials

- **Type status:** Other material. **Occurrence:** recordedBy: Kuntner, Čandek; sex: 1 female; **Location:** locationID: SI50; country: Slovenia; locality: Sp. Prapreče; minimumElevationInMeters: 351; maximumElevationInMeters: 351; decimalLatitude: 46.1620; decimalLongitude: 14.6933; **Event:** eventDate: 2010-08-03/2012-05-28; habitat: house and surroundings

#### Anyphaena sabina

L. Koch, 1866

##### Materials

- **Type status:** Other material. **Occurrence:** recordedBy: Kuntner, Gregorič, Čandek, Kralj-Fišer, Cheng; sex: 1 female; **Location:** locationID: SI41; country: Slovenia; locality: Socerb, Osp; minimumElevationInMeters: 116; maximumElevationInMeters: 116; decimalLatitude: 45.5819; decimalLongitude: 13.8558; **Event:** eventDate: 2012-06-07; habitat: trail from Socerb to Osp

#### Araneidae

Clerck, 1757

#### Aculepeira ceropegia

(Walckenaer, 1802)

##### Materials

- **Type status:** Other material. **Occurrence:** recordedBy: Kuntner, Gregorič, Čandek; sex: 1 female, 1 male; **Location:** locationID: CH06; country: Switzerland; locality: Bernese Alps, Kandersteg; minimumElevationInMeters: 1677; maximumElevationInMeters: 1677; decimalLatitude: 46.5020; decimalLongitude: 7.6992; **Event:** eventDate: 2011-07-07; habitat: alpine meadow
- **Type status:** Other material. **Occurrence:** recordedBy: Kuntner, Gregorič, Čandek; sex: 2 females; **Location:** locationID: CH09; country: Switzerland; locality: Pennine Alps, Mattertal; minimumElevationInMeters: 1447; maximumElevationInMeters: 1447; decimalLatitude: 46.0976; decimalLongitude: 7.7789; **Event:** eventDate: 2011-07-08; habitat: forest and meadow near river
- **Type status:** Other material. **Occurrence:** recordedBy: Kuntner, Gregorič, Čandek; sex: 1 female; **Location:** locationID: CH17; country: Switzerland; locality: Engadin, Bivio; minimumElevationInMeters: 1780; maximumElevationInMeters: 1780; decimalLatitude: 46.4753; decimalLongitude: 9.6469; **Event:** eventDate: 2011-07-11; habitat: forest and river edge
- **Type status:** Other material. **Occurrence:** recordedBy: Kuntner, Gregorič, Čandek; sex: 1 male; **Location:** locationID: CH19; country: Switzerland; locality: Grison Alps, Alp Flix, Salategnas; minimumElevationInMeters: 1910; maximumElevationInMeters: 1910; decimalLatitude: 46.5172; decimalLongitude: 9.6533; **Event:** eventDate: 2011-07-12; habitat: flat uncut grassland
- **Type status:** Other material. **Occurrence:** recordedBy: Kuntner, Gregorič, Čandek; sex: 1 female; **Location:** locationID: CH23; country: Switzerland; locality: Grison Alps, Alp Flix, Salategnas; minimumElevationInMeters: 1900; maximumElevationInMeters: 1900; decimalLatitude: 46.5141; decimalLongitude: 9.6448; **Event:** eventDate: 2011-07-12; habitat: forest opening, grass and shrubs
- **Type status:** Other material. **Occurrence:** recordedBy: Kuntner, Gregorič, Čandek; sex: 1 female; **Location:** locationID: CH32; country: Switzerland; locality: Grison Alps, Alp Flix, Salategnas; minimumElevationInMeters: 1955; maximumElevationInMeters: 1955; decimalLatitude: 46.5203; decimalLongitude: 9.6458; **Event:** eventDate: 2011-07-16; habitat: timberline forest, moss
- **Type status:** Other material. **Occurrence:** recordedBy: Čandek; sex: 1 female, 1 male; **Location:** locationID: SI61; country: Slovenia; locality: Sekirišče; minimumElevationInMeters: 750; maximumElevationInMeters: 750; decimalLatitude: 45.8631; decimalLongitude: 14.5367; **Event:** eventDate: 2011-06-23/2012-06-21; habitat: house, grassland, overgrowth

#### Agalenatea redii

(Scopoli, 1763)

##### Materials

- **Type status:** Other material. **Occurrence:** recordedBy: Čandek; sex: 2 females; **Location:** locationID: SI38; country: Slovenia; locality: Poreče; minimumElevationInMeters: 135; maximumElevationInMeters: 135; decimalLatitude: 45.8188; decimalLongitude: 13.9692; **Event:** eventDate: 2011-05-08; habitat: grassland
- **Type status:** Other material. **Occurrence:** recordedBy: Čandek; sex: 1 female; **Location:** locationID: SI43; country: Slovenia; locality: Vipava; minimumElevationInMeters: 114; maximumElevationInMeters: 114; decimalLatitude: 45.8282; decimalLongitude: 13.9594; **Event:** eventDate: 2011-05-08; habitat: grassland

#### Araneus alsine

(Walckenaer, 1802)

##### Materials

- **Type status:** Other material. **Occurrence:** recordedBy: Čandek; sex: 1 male; **Location:** locationID: SI61; country: Slovenia; locality: Sekirišče; minimumElevationInMeters: 750; maximumElevationInMeters: 750; decimalLatitude: 45.8631; decimalLongitude: 14.5367; **Event:** eventDate: 2011-06-23/2012-06-21; habitat: house, grassland, overgrowth

#### Araneus angulatus

Clerck, 1757

##### Materials

- **Type status:** Other material. **Occurrence:** recordedBy: Kostanjšek, RTŠB 2011; sex: 1 male; **Location:** locationID: SI19; country: Slovenia; locality: Ptujska cesta; minimumElevationInMeters: 240; maximumElevationInMeters: 240; decimalLatitude: 46.6283; decimalLongitude: 15.9973; **Event:** eventDate: 2011-07-26; habitat: grassland
- **Type status:** Other material. **Occurrence:** recordedBy: Gregorič, Čandek, Kralj-Fišer; sex: 2 females; **Location:** locationID: SI52; country: Slovenia; locality: Dinaric Karst, Griže; minimumElevationInMeters: 484; maximumElevationInMeters: 484; decimalLatitude: 45.7506; decimalLongitude: 13.9509; **Event:** eventDate: 2011-04-04/05-10; habitat: overgrowth
- **Type status:** Other material. **Occurrence:** recordedBy: Kuntner; sex: 1 female; **Location:** locationID: SI63; country: Slovenia; locality: Avče; minimumElevationInMeters: 165; maximumElevationInMeters: 165; decimalLatitude: 46.1088; decimalLongitude: 13.6819; **Event:** eventDate: 2010-07-26; habitat: not specified

#### Araneus diadematus

Clerck, 1757

##### Materials

- **Type status:** Other material. **Occurrence:** recordedBy: Kuntner, Gregorič, Čandek; sex: 2 females, 1 male; **Location:** locationID: CH04; country: Switzerland; locality: Bernese Alps, Gasteretal; minimumElevationInMeters: 1460; maximumElevationInMeters: 1460; decimalLatitude: 46.4552; decimalLongitude: 7.7039; **Event:** eventDate: 2011-07-07; habitat: river shore
- **Type status:** Other material. **Occurrence:** recordedBy: Kuntner, Lokovšek; sex: 2 females; **Location:** locationID: SI40; country: Slovenia; locality: Slavnik; minimumElevationInMeters: 816; maximumElevationInMeters: 816; decimalLatitude: 45.5499; decimalLongitude: 13.9619; **Event:** eventDate: 2010-08-26; habitat: grassland and forest
- **Type status:** Other material. **Occurrence:** recordedBy: Kuntner, Čandek; sex: 3 females; **Location:** locationID: SI50; country: Slovenia; locality: Sp. Prapreče; minimumElevationInMeters: 351; maximumElevationInMeters: 351; decimalLatitude: 46.1620; decimalLongitude: 14.6933; **Event:** eventDate: 2010-08-03/2012-05-28; habitat: house and surroundings
- **Type status:** Other material. **Occurrence:** recordedBy: Kostanjšek, RTŠB 2011; sex: 1 female; **Location:** locationID: SI64; country: Slovenia; locality: Polenšak; minimumElevationInMeters: 225; maximumElevationInMeters: 225; decimalLatitude: 46.4723; decimalLongitude: 16.0182; **Event:** eventDate: 2011-07-29; habitat: marshy grassland

#### Araneus marmoreus

Clerck, 1757

##### Materials

- **Type status:** Other material. **Occurrence:** recordedBy: Kuntner, Gregorič, Lokovšek; sex: 1 female; **Location:** locationID: SI39; country: Slovenia; locality: Primostek; minimumElevationInMeters: 157; maximumElevationInMeters: 157; decimalLatitude: 45.6299; decimalLongitude: 15.2997; **Event:** eventDate: 2010-08-24; habitat: grassland
- **Type status:** Other material. **Occurrence:** recordedBy: Kuntner, Čandek; sex: 2 females; **Location:** locationID: SI50; country: Slovenia; locality: Sp. Prapreče; minimumElevationInMeters: 351; maximumElevationInMeters: 351; decimalLatitude: 46.1620; decimalLongitude: 14.6933; **Event:** eventDate: 2010-08-03/2012-05-28; habitat: house and surroundings
- **Type status:** Other material. **Occurrence:** recordedBy: Čandek; sex: 1 female; **Location:** locationID: SI65; country: Slovenia; locality: Dramlje; minimumElevationInMeters: 409; maximumElevationInMeters: 409; decimalLatitude: 46.2799; decimalLongitude: 15.4044; **Event:** eventDate: 2011-08-27; habitat: house and surroundings
- **Type status:** Other material. **Occurrence:** recordedBy: Kostanjšek, RTŠB 2011; sex: 1 female; **Location:** locationID: SI76; country: Slovenia; locality: Velovlek; minimumElevationInMeters: 225; maximumElevationInMeters: 225; decimalLatitude: 46.4942; decimalLongitude: 15.9174; **Event:** eventDate: 2011-07-25; habitat: forest

#### Araneus quadratus

Clerck, 1757

##### Materials

- **Type status:** Other material. **Occurrence:** recordedBy: Kuntner, Gregorič, Čandek; sex: 3 males; **Location:** locationID: CH23; country: Switzerland; locality: Grison Alps, Alp Flix, Salategnas; minimumElevationInMeters: 1900; maximumElevationInMeters: 1900; decimalLatitude: 46.5141; decimalLongitude: 9.6448; **Event:** eventDate: 2011-07-12; habitat: forest opening, grass and shrubs
- **Type status:** Other material. **Occurrence:** recordedBy: Kuntner, Gregorič, Čandek; sex: 1 female; **Location:** locationID: CH30; country: Switzerland; locality: Grison Alps, Alp Flix - Lai Flix; minimumElevationInMeters: 1967; maximumElevationInMeters: 1967; decimalLatitude: 46.5358; decimalLongitude: 9.6409; **Event:** eventDate: 2011-07-16; habitat: next to alpine lake
- **Type status:** Other material. **Occurrence:** recordedBy: Kuntner, Gregorič, Čandek; sex: 1 male; **Location:** locationID: CH32; country: Switzerland; locality: Grison Alps, Alp Flix, Salategnas; minimumElevationInMeters: 1955; maximumElevationInMeters: 1955; decimalLatitude: 46.5203; decimalLongitude: 9.6458; **Event:** eventDate: 2011-07-16; habitat: timberline forest, moss
- **Type status:** Other material. **Occurrence:** recordedBy: Gregorič; sex: 1 female; **Location:** locationID: SI44; country: Slovenia; locality: Vnanje Gorice, Brezovica pri Ljubljani; minimumElevationInMeters: 289; maximumElevationInMeters: 289; decimalLatitude: 46.0028; decimalLongitude: 14.4342; **Event:** eventDate: 2010-09-10; habitat: house
- **Type status:** Other material. **Occurrence:** recordedBy: Čandek; sex: 1 female; **Location:** locationID: SI46; country: Slovenia; locality: Šešče pri Preboldu; minimumElevationInMeters: 284; maximumElevationInMeters: 285; decimalLatitude: 46.2356; decimalLongitude: 15.1228; **Event:** eventDate: 2011-06-13/2012-06-22; habitat: house and surroundings

#### Araneus sturmi

(Hahn, 1831)

##### Materials

- **Type status:** Other material. **Occurrence:** recordedBy: Kuntner, Gregorič, Čandek, Kralj-Fišer, Cheng; sex: 1 female; **Location:** locationID: SI41; country: Slovenia; locality: Socerb, Osp; minimumElevationInMeters: 116; maximumElevationInMeters: 116; decimalLatitude: 45.5819; decimalLongitude: 13.8558; **Event:** eventDate: 2012-06-07; habitat: trail from Socerb to Osp
- **Type status:** Other material. **Occurrence:** recordedBy: Čandek; sex: 1 male; **Location:** locationID: SI61; country: Slovenia; locality: Sekirišče; minimumElevationInMeters: 750; maximumElevationInMeters: 750; decimalLatitude: 45.8631; decimalLongitude: 14.5367; **Event:** eventDate: 2011-06-23/2012-06-21; habitat: house, grassland, overgrowth

#### Araniella cucurbitina

(Clerck, 1757)

##### Materials

- **Type status:** Other material. **Occurrence:** recordedBy: Kuntner, Gregorič, Čandek, Kralj-Fišer, Cheng; sex: 1 female; **Location:** locationID: SI41; country: Slovenia; locality: Socerb, Osp; minimumElevationInMeters: 116; maximumElevationInMeters: 116; decimalLatitude: 45.5819; decimalLongitude: 13.8558; **Event:** eventDate: 2012-06-07; habitat: trail from Socerb to Osp
- **Type status:** Other material. **Occurrence:** recordedBy: Kuntner, Čandek; sex: 2 males; **Location:** locationID: SI50; country: Slovenia; locality: Sp. Prapreče; minimumElevationInMeters: 351; maximumElevationInMeters: 351; decimalLatitude: 46.1620; decimalLongitude: 14.6933; **Event:** eventDate: 2010-08-03/2012-05-28; habitat: house and surroundings
- **Type status:** Other material. **Occurrence:** recordedBy: Čandek; sex: 1 male; **Location:** locationID: SI58; country: Slovenia; locality: Budanje; minimumElevationInMeters: 243; maximumElevationInMeters: 243; decimalLatitude: 45.8743; decimalLongitude: 13.9497; **Event:** eventDate: 2011-05-07; habitat: school and surroundings

#### Araniella displicata

(Hentz, 1847)

##### Materials

- **Type status:** Other material. **Occurrence:** recordedBy: Kuntner, Gregorič, Čandek; sex: 1 female; **Location:** locationID: CH32; country: Switzerland; locality: Grison Alps, Alp Flix, Salategnas; minimumElevationInMeters: 1955; maximumElevationInMeters: 1955; decimalLatitude: 46.5203; decimalLongitude: 9.6458; **Event:** eventDate: 2011-07-16; habitat: timberline forest, moss

#### Argiope bruennichi

(Scopoli, 1772)

##### Materials

- **Type status:** Other material. **Occurrence:** recordedBy: Kostanjšek, RTŠB 2011; sex: 1 male; **Location:** locationID: SI04; country: Slovenia; locality: Cerkvenjak; minimumElevationInMeters: 230; maximumElevationInMeters: 230; decimalLatitude: 46.5641; decimalLongitude: 15.9863; **Event:** eventDate: 2011-07-22; habitat: grassland
- **Type status:** Other material. **Occurrence:** recordedBy: Kuntner, Lokovšek; sex: 2 females; **Location:** locationID: SI40; country: Slovenia; locality: Slavnik; minimumElevationInMeters: 816; maximumElevationInMeters: 816; decimalLatitude: 45.5499; decimalLongitude: 13.9619; **Event:** eventDate: 2010-08-26; habitat: grassland and forest
- **Type status:** Other material. **Occurrence:** recordedBy: Kuntner, Čandek; sex: 4 females; **Location:** locationID: SI50; country: Slovenia; locality: Sp. Prapreče; minimumElevationInMeters: 351; maximumElevationInMeters: 351; decimalLatitude: 46.1620; decimalLongitude: 14.6933; **Event:** eventDate: 2010-08-03/2012-05-28; habitat: house and surroundings
- **Type status:** Other material. **Occurrence:** recordedBy: Čandek; sex: 1 female; **Location:** locationID: SI61; country: Slovenia; locality: Sekirišče; minimumElevationInMeters: 750; maximumElevationInMeters: 750; decimalLatitude: 45.8631; decimalLongitude: 14.5367; **Event:** eventDate: 2011-06-23/2012-06-21; habitat: house, grassland, overgrowth

#### Cercidia prominens

(Westring, 1851)

##### Materials

- **Type status:** Other material. **Occurrence:** recordedBy: Kuntner, Gregorič, Čandek, Kralj-Fišer, Cheng; sex: 1 female; **Location:** locationID: SI41; country: Slovenia; locality: Socerb, Osp; minimumElevationInMeters: 116; maximumElevationInMeters: 116; decimalLatitude: 45.5819; decimalLongitude: 13.8558; **Event:** eventDate: 2012-06-07; habitat: trail from Socerb to Osp
- **Type status:** Other material. **Occurrence:** recordedBy: Gregorič, Čandek, Kralj-Fišer; sex: 2 males; **Location:** locationID: SI52; country: Slovenia; locality: Dinaric Karst, Griže; minimumElevationInMeters: 484; maximumElevationInMeters: 484; decimalLatitude: 45.7506; decimalLongitude: 13.9509; **Event:** eventDate: 2011-04-04/05-10; habitat: overgrowth
- **Type status:** Other material. **Occurrence:** recordedBy: Gregorič, Čandek, Kralj-Fišer; sex: 2 females; **Location:** locationID: SI55; country: Slovenia; locality: Dinaric Karst, Lokvice; minimumElevationInMeters: 273; maximumElevationInMeters: 275; decimalLatitude: 45.8659; decimalLongitude: 13.6102; **Event:** eventDate: 2011-04-04/05-10; habitat: overgrowth
- **Type status:** Other material. **Occurrence:** recordedBy: Čandek; sex: 1 female, 1 male; **Location:** locationID: SI60; country: Slovenia; locality: Budanje; minimumElevationInMeters: 295; maximumElevationInMeters: 295; decimalLatitude: 45.8799; decimalLongitude: 13.9459; **Event:** eventDate: 2011-05-07; habitat: forest clearing

#### Cyclosa conica

(Pallas, 1772)

##### Materials

- **Type status:** Other material. **Occurrence:** recordedBy: Kuntner, Gregorič, Čandek; sex: 1 female; **Location:** locationID: CH01; country: Switzerland; locality: Bernese Alps, Gasteretal; minimumElevationInMeters: 1662; maximumElevationInMeters: 1662; decimalLatitude: 46.4457; decimalLongitude: 7.7413; **Event:** eventDate: 2011-07-07; habitat: alpine meadow
- **Type status:** Other material. **Occurrence:** recordedBy: Kuntner, Lokovšek; sex: 1 female; **Location:** locationID: SI40; country: Slovenia; locality: Slavnik; minimumElevationInMeters: 816; maximumElevationInMeters: 816; decimalLatitude: 45.5499; decimalLongitude: 13.9619; **Event:** eventDate: 2010-08-26; habitat: grassland and forest

#### Gibbaranea bituberculata

(Walckenaer, 1802)

##### Materials

- **Type status:** Other material. **Occurrence:** recordedBy: Kuntner, Gregorič, Čandek, Kralj-Fišer, Cheng; sex: 2 females; **Location:** locationID: SI41; country: Slovenia; locality: Socerb, Osp; minimumElevationInMeters: 116; maximumElevationInMeters: 116; decimalLatitude: 45.5819; decimalLongitude: 13.8558; **Event:** eventDate: 2012-06-07; habitat: trail from Socerb to Osp
- **Type status:** Other material. **Occurrence:** recordedBy: Čandek; sex: 3 females; **Location:** locationID: SI43; country: Slovenia; locality: Vipava; minimumElevationInMeters: 114; maximumElevationInMeters: 114; decimalLatitude: 45.8282; decimalLongitude: 13.9594; **Event:** eventDate: 2011-05-08; habitat: grassland
- **Type status:** Other material. **Occurrence:** recordedBy: Gregorič, Čandek, Kralj-Fišer; sex: 1 female, 1 male; **Location:** locationID: SI52; country: Slovenia; locality: Dinaric Karst, Griže; minimumElevationInMeters: 484; maximumElevationInMeters: 484; decimalLatitude: 45.7506; decimalLongitude: 13.9509; **Event:** eventDate: 2011-04-04/05-10; habitat: overgrowth
- **Type status:** Other material. **Occurrence:** recordedBy: Čandek; sex: 1 female; **Location:** locationID: SI60; country: Slovenia; locality: Budanje; minimumElevationInMeters: 295; maximumElevationInMeters: 295; decimalLatitude: 45.8799; decimalLongitude: 13.9459; **Event:** eventDate: 2011-05-07; habitat: forest clearing

#### Hypsosinga albovittata

(Westring, 1851)

##### Materials

- **Type status:** Other material. **Occurrence:** recordedBy: Kuntner, Gregorič, Čandek; sex: 1 female; **Location:** locationID: CH08; country: Switzerland; locality: Bernese Alps, Rothorn; minimumElevationInMeters: 2250; maximumElevationInMeters: 2250; decimalLatitude: 46.0183; decimalLongitude: 7.7687; **Event:** eventDate: 2011-07-08; habitat: grass, shrubs, spruce

#### Hypsosinga pygmaea

(Sundevall, 1831)

##### Materials

- **Type status:** Other material. **Occurrence:** recordedBy: Kostanjšek, RTŠB 2011; sex: 1 female; **Location:** locationID: SI20; country: Slovenia; locality: Dragotinci; minimumElevationInMeters: 225; maximumElevationInMeters: 225; decimalLatitude: 46.5885; decimalLongitude: 16.0297; **Event:** eventDate: 2011-07-27; habitat: grassland
- **Type status:** Other material. **Occurrence:** recordedBy: Kostanjšek, RTŠB 2012; sex: 1 female; **Location:** locationID: SI26; country: Slovenia; locality: Dolnja Košana; minimumElevationInMeters: 420; maximumElevationInMeters: 420; decimalLatitude: 45.6587; decimalLongitude: 14.1397; **Event:** eventDate: 2012-07-21; habitat: grassland

#### Hypsosinga sanguinea

(C. L. Koch, 1844)

##### Materials

- **Type status:** Other material. **Occurrence:** recordedBy: Kuntner, Gregorič, Čandek; sex: 1 female; **Location:** locationID: CH11; country: Switzerland; locality: Bernese Alps, Lake Brienz; minimumElevationInMeters: 600; maximumElevationInMeters: 600; decimalLatitude: 46.7569; decimalLongitude: 8.0107; **Event:** eventDate: 2011-07-10; habitat: meadows and forest
- **Type status:** Other material. **Occurrence:** recordedBy: Gregorič, Čandek; sex: 2 females, 2 males; **Location:** locationID: SI57; country: Slovenia; locality: Dinaric Karst, Novelo; minimumElevationInMeters: 325; maximumElevationInMeters: 325; decimalLatitude: 45.8482; decimalLongitude: 13.6584; **Event:** eventDate: 2011-05-10; habitat: grassland

#### Larinioides sclopetarius

(Clerck, 1757)

##### Materials

- **Type status:** Other material. **Occurrence:** recordedBy: Kuntner, Gregorič, Čandek; sex: 1 female; **Location:** locationID: CH15; country: Switzerland; locality: Glarus Alps, near Affeier; minimumElevationInMeters: 817; maximumElevationInMeters: 817; decimalLatitude: 46.7606; decimalLongitude: 9.0933; **Event:** eventDate: 2011-07-10; habitat: meadow and forest
- **Type status:** Other material. **Occurrence:** recordedBy: Čandek; sex: 2 males; **Location:** locationID: SI49; country: Slovenia; locality: Ljubljana, center; minimumElevationInMeters: 287; maximumElevationInMeters: 287; decimalLatitude: 46.0516; decimalLongitude: 14.5107; **Event:** eventDate: 2011-08-25; habitat: house
- **Type status:** Other material. **Occurrence:** recordedBy: Gregorič; sex: 2 females; **Location:** locationID: SI66; country: Slovenia; locality: Kremenica, Ig; minimumElevationInMeters: 309; maximumElevationInMeters: 309; decimalLatitude: 45.9415; decimalLongitude: 14.5480; **Event:** eventDate: 2010-09-10; habitat: lake edge

#### Leviellus thorelli

(Ausserer, 1871)

##### Materials

- **Type status:** Other material. **Occurrence:** recordedBy: Kostanjšek, RTŠB 2011; sex: 1 female; **Location:** locationID: SI16; country: Slovenia; locality: Apače; minimumElevationInMeters: 215; maximumElevationInMeters: 215; decimalLatitude: 46.6954; decimalLongitude: 15.9087; **Event:** eventDate: 2011-07-26; habitat: house and surroundings
- **Type status:** Other material. **Occurrence:** recordedBy: Kuntner, Čandek; sex: 2 females; **Location:** locationID: SI50; country: Slovenia; locality: Sp. Prapreče; minimumElevationInMeters: 351; maximumElevationInMeters: 351; decimalLatitude: 46.1620; decimalLongitude: 14.6933; **Event:** eventDate: 2010-08-03/2012-05-28; habitat: house and surroundings
- **Type status:** Other material. **Occurrence:** recordedBy: Čandek; sex: 1 female; **Location:** locationID: SI65; country: Slovenia; locality: Dramlje; minimumElevationInMeters: 409; maximumElevationInMeters: 409; decimalLatitude: 46.2799; decimalLongitude: 15.4044; **Event:** eventDate: 2011-08-27; habitat: house and surroundings

#### Mangora acalypha

(Walckenaer, 1802)

##### Materials

- **Type status:** Other material. **Occurrence:** recordedBy: Čandek; sex: 1 female, 2 males; **Location:** locationID: SI38; country: Slovenia; locality: Poreče; minimumElevationInMeters: 135; maximumElevationInMeters: 135; decimalLatitude: 45.8188; decimalLongitude: 13.9692; **Event:** eventDate: 2011-05-08; habitat: grassland
- **Type status:** Other material. **Occurrence:** recordedBy: Kuntner, Gregorič, Čandek, Kralj-Fišer, Cheng; sex: 1 female; **Location:** locationID: SI41; country: Slovenia; locality: Socerb, Osp; minimumElevationInMeters: 116; maximumElevationInMeters: 116; decimalLatitude: 45.5819; decimalLongitude: 13.8558; **Event:** eventDate: 2012-06-07; habitat: trail from Socerb to Osp
- **Type status:** Other material. **Occurrence:** recordedBy: Čandek; sex: 5 females, 1 male; **Location:** locationID: SI43; country: Slovenia; locality: Vipava; minimumElevationInMeters: 114; maximumElevationInMeters: 114; decimalLatitude: 45.8282; decimalLongitude: 13.9594; **Event:** eventDate: 2011-05-08; habitat: grassland
- **Type status:** Other material. **Occurrence:** recordedBy: Kuntner, Čandek; sex: 1 female; **Location:** locationID: SI50; country: Slovenia; locality: Sp. Prapreče; minimumElevationInMeters: 351; maximumElevationInMeters: 351; decimalLatitude: 46.1620; decimalLongitude: 14.6933; **Event:** eventDate: 2010-08-03/2012-05-28; habitat: house and surroundings
- **Type status:** Other material. **Occurrence:** recordedBy: Gregorič, Čandek, Kralj-Fišer; sex: 1 female; **Location:** locationID: SI52; country: Slovenia; locality: Dinaric Karst, Griže; minimumElevationInMeters: 484; maximumElevationInMeters: 484; decimalLatitude: 45.7506; decimalLongitude: 13.9509; **Event:** eventDate: 2011-04-04/05-10; habitat: overgrowth
- **Type status:** Other material. **Occurrence:** recordedBy: Gregorič, Čandek; sex: 1 female; **Location:** locationID: SI53; country: Slovenia; locality: Dinaric Karst, Griže; minimumElevationInMeters: 434; maximumElevationInMeters: 434; decimalLatitude: 45.7548; decimalLongitude: 13.9495; **Event:** eventDate: 2011-05-10/2011-06-21; habitat: grassland
- **Type status:** Other material. **Occurrence:** recordedBy: Gregorič, Čandek, Kralj-Fišer; sex: 4 females, 1 male; **Location:** locationID: SI55; country: Slovenia; locality: Dinaric Karst, Lokvice; minimumElevationInMeters: 273; maximumElevationInMeters: 275; decimalLatitude: 45.8659; decimalLongitude: 13.6102; **Event:** eventDate: 2011-04-04/05-10; habitat: overgrowth
- **Type status:** Other material. **Occurrence:** recordedBy: Gregorič, Čandek, Kralj-Fišer; sex: 3 males; **Location:** locationID: SI56; country: Slovenia; locality: Dinaric Karst, Novelo; minimumElevationInMeters: 358; maximumElevationInMeters: 359; decimalLatitude: 45.8533; decimalLongitude: 13.6552; **Event:** eventDate: 2011-04-04/05-10; habitat: overgrowth
- **Type status:** Other material. **Occurrence:** recordedBy: Čandek; sex: 3 males; **Location:** locationID: SI60; country: Slovenia; locality: Budanje; minimumElevationInMeters: 295; maximumElevationInMeters: 295; decimalLatitude: 45.8799; decimalLongitude: 13.9459; **Event:** eventDate: 2011-05-07; habitat: forest clearing
- **Type status:** Other material. **Occurrence:** recordedBy: Kostanjšek, RTŠB 2011; sex: 1 female; **Location:** locationID: SI67; country: Slovenia; locality: Sv. Jurij ob Ščavnici; minimumElevationInMeters: 190; maximumElevationInMeters: 190; decimalLatitude: 46.5578; decimalLongitude: 16.0395; **Event:** eventDate: 2011-07-25; habitat: river edge

#### Neoscona adianta

(Walckenaer, 1802)

##### Materials

- **Type status:** Other material. **Occurrence:** recordedBy: Gregorič, Čandek; sex: 1 female; **Location:** locationID: SI53; country: Slovenia; locality: Dinaric Karst, Griže; minimumElevationInMeters: 434; maximumElevationInMeters: 434; decimalLatitude: 45.7548; decimalLongitude: 13.9495; **Event:** eventDate: 2011-05-10/2011-06-21; habitat: grassland

#### Nuctenea umbratica

(Clerck, 1757)

##### Materials

- **Type status:** Other material. **Occurrence:** recordedBy: Kuntner, Gregorič, Čandek; sex: 1 female; **Location:** locationID: CH10; country: Switzerland; locality: Bernese Alps, Kleine Scheidegg; minimumElevationInMeters: 2061; maximumElevationInMeters: 2061; decimalLatitude: 46.5853; decimalLongitude: 7.9606; **Event:** eventDate: 2011-07-09; habitat: grassland
- **Type status:** Other material. **Occurrence:** recordedBy: Kuntner, Gregorič, Čandek; sex: 1 female; **Location:** locationID: CH12; country: Switzerland; locality: Bernese Alps, Nessental; minimumElevationInMeters: 930; maximumElevationInMeters: 930; decimalLatitude: 46.7213; decimalLongitude: 8.3039; **Event:** eventDate: 2011-07-10; habitat: grassland and loan trees
- **Type status:** Other material. **Occurrence:** recordedBy: Kuntner, Čandek; sex: 2 females, 1 male; **Location:** locationID: SI50; country: Slovenia; locality: Sp. Prapreče; minimumElevationInMeters: 351; maximumElevationInMeters: 351; decimalLatitude: 46.1620; decimalLongitude: 14.6933; **Event:** eventDate: 2010-08-03/2012-05-28; habitat: house and surroundings
- **Type status:** Other material. **Occurrence:** recordedBy: Kostanjšek, RTŠB 2011; sex: 1 male; **Location:** locationID: SI68; country: Slovenia; locality: Sv. Jurij ob Ščavnici; minimumElevationInMeters: 235; maximumElevationInMeters: 235; decimalLatitude: 46.5687; decimalLongitude: 16.0223; **Event:** eventDate: 2011-07-22; habitat: school

#### Parazygiella montana

(C. L. Koch, 1834)

##### Materials

- **Type status:** Other material. **Occurrence:** recordedBy: Kuntner, Gregorič, Čandek; sex: 1 female; **Location:** locationID: CH01; country: Switzerland; locality: Bernese Alps, Gasteretal; minimumElevationInMeters: 1662; maximumElevationInMeters: 1662; decimalLatitude: 46.4457; decimalLongitude: 7.7413; **Event:** eventDate: 2011-07-07; habitat: alpine meadow
- **Type status:** Other material. **Occurrence:** recordedBy: Kuntner, Gregorič, Čandek; sex: 2 females; **Location:** locationID: CH06; country: Switzerland; locality: Bernese Alps, Kandersteg; minimumElevationInMeters: 1677; maximumElevationInMeters: 1677; decimalLatitude: 46.5020; decimalLongitude: 7.6992; **Event:** eventDate: 2011-07-07; habitat: alpine meadow
- **Type status:** Other material. **Occurrence:** recordedBy: Kuntner, Gregorič, Čandek; sex: 1 female, 1 male; **Location:** locationID: CH10; country: Switzerland; locality: Bernese Alps, Kleine Scheidegg; minimumElevationInMeters: 2061; maximumElevationInMeters: 2061; decimalLatitude: 46.5853; decimalLongitude: 7.9606; **Event:** eventDate: 2011-07-09; habitat: grassland
- **Type status:** Other material. **Occurrence:** recordedBy: Kuntner, Gregorič, Čandek; sex: 1 female; **Location:** locationID: CH23; country: Switzerland; locality: Grison Alps, Alp Flix, Salategnas; minimumElevationInMeters: 1900; maximumElevationInMeters: 1900; decimalLatitude: 46.5141; decimalLongitude: 9.6448; **Event:** eventDate: 2011-07-12; habitat: forest opening, grass and shrubs
- **Type status:** Other material. **Occurrence:** recordedBy: Kuntner; sex: 2 females; **Location:** locationID: SI42; country: Slovenia; locality: Srednja vas, Bohinj; minimumElevationInMeters: 1348; maximumElevationInMeters: 1348; decimalLatitude: 46.3458; decimalLongitude: 13.9232; **Event:** eventDate: 2010-09-28; habitat: house and surroundings

#### Singa nitidula

C. L. Koch, 1844

##### Materials

- **Type status:** Other material. **Occurrence:** recordedBy: Čandek; sex: 1 male; **Location:** locationID: SI33; country: Slovenia; locality: Ljubljana, Biotechnical faculty; minimumElevationInMeters: 297; maximumElevationInMeters: 297; decimalLatitude: 46.0513; decimalLongitude: 14.4700; **Event:** eventDate: 2012-05-09; habitat: house and surroundings

#### Stroemiellus stroemi

(Thorell, 1870)

##### Materials

- **Type status:** Other material. **Occurrence:** recordedBy: Gregorič, Kuntner, Čandek; sex: 2 females; **Location:** locationID: SI48; country: Slovenia; locality: Ljubljana, center; minimumElevationInMeters: 291; maximumElevationInMeters: 291; decimalLatitude: 46.0434; decimalLongitude: 14.5041; **Event:** eventDate: 2011-05-24/2012-06-19; habitat: house

#### Zilla diodia

(Walckenaer, 1802)

##### Materials

- **Type status:** Other material. **Occurrence:** recordedBy: Kuntner, Gregorič, Čandek, Kralj-Fišer, Cheng; sex: 2 females; **Location:** locationID: SI41; country: Slovenia; locality: Socerb, Osp; minimumElevationInMeters: 116; maximumElevationInMeters: 116; decimalLatitude: 45.5819; decimalLongitude: 13.8558; **Event:** eventDate: 2012-06-07; habitat: trail from Socerb to Osp
- **Type status:** Other material. **Occurrence:** recordedBy: Čandek; sex: 4 females; **Location:** locationID: SI58; country: Slovenia; locality: Budanje; minimumElevationInMeters: 243; maximumElevationInMeters: 243; decimalLatitude: 45.8743; decimalLongitude: 13.9497; **Event:** eventDate: 2011-05-07; habitat: school and surroundings
- **Type status:** Other material. **Occurrence:** recordedBy: Čandek; sex: 1 female, 1 male; **Location:** locationID: SI60; country: Slovenia; locality: Budanje; minimumElevationInMeters: 295; maximumElevationInMeters: 295; decimalLatitude: 45.8799; decimalLongitude: 13.9459; **Event:** eventDate: 2011-05-07; habitat: forest clearing

#### Zygiella x-notata

(Clerck, 1757)

##### Materials

- **Type status:** Other material. **Occurrence:** recordedBy: Kuntner, Gregorič, Čandek; sex: 1 female; **Location:** locationID: CH33; country: Switzerland; locality: Interlaken; minimumElevationInMeters: 1662; maximumElevationInMeters: 1662; decimalLatitude: 46.4457; decimalLongitude: 7.7413; **Event:** eventDate: 2011-07-10; habitat: house

#### Atypidae

Thorell, 1870

#### Atypus piceus

(Sulzer, 1776)

##### Materials

- **Type status:** Other material. **Occurrence:** recordedBy: Kuntner, Lokovšek; sex: 2 females; **Location:** locationID: SI40; country: Slovenia; locality: Slavnik; minimumElevationInMeters: 816; maximumElevationInMeters: 816; decimalLatitude: 45.5499; decimalLongitude: 13.9619; **Event:** eventDate: 2010-08-26; habitat: grassland and forest
- **Type status:** Other material. **Occurrence:** recordedBy: Kuntner; sex: 1 female; **Location:** locationID: SI69; country: Slovenia; locality: Lipalca; minimumElevationInMeters: 359; maximumElevationInMeters: 359; decimalLatitude: 46.0102; decimalLongitude: 14.3116; **Event:** eventDate: 2012-01-26; habitat: inside tree log

#### Clubionidae

Wagner, 1887

#### Clubiona germanica

Thorell, 1871

##### Materials

- **Type status:** Other material. **Occurrence:** recordedBy: Kostanjšek, RTŠB 2011; sex: 1 female; **Location:** locationID: SI18; country: Slovenia; locality: Podgorje; minimumElevationInMeters: 220; maximumElevationInMeters: 220; decimalLatitude: 46.7183; decimalLongitude: 15.8243; **Event:** eventDate: 2011-07-26; habitat: vegetation at trail

#### Clubiona kulczynskii

Lessert, 1905

##### Materials

- **Type status:** Other material. **Occurrence:** recordedBy: Kuntner, Gregorič, Čandek; sex: 1 female; **Location:** locationID: CH05; country: Switzerland; locality: Bernese Alps, Gasteretal; minimumElevationInMeters: 1380; maximumElevationInMeters: 1380; decimalLatitude: 46.4674; decimalLongitude: 7.6640; **Event:** eventDate: 2011-07-07; habitat: river vegetation

#### Clubiona neglecta

O. P.-Cambridge, 1862

##### Materials

- **Type status:** Other material. **Occurrence:** recordedBy: Čandek; sex: 1 female; **Location:** locationID: SI61; country: Slovenia; locality: Sekirišče; minimumElevationInMeters: 750; maximumElevationInMeters: 750; decimalLatitude: 45.8631; decimalLongitude: 14.5367; **Event:** eventDate: 2011-06-23/2012-06-21; habitat: house, grassland, overgrowth

#### Clubiona pseudoneglecta

Wunderlich, 1994

##### Materials

- **Type status:** Other material. **Occurrence:** recordedBy: Kostanjšek, RTŠB 2012; sex: 1 female, 1 male; **Location:** locationID: SI25; country: Slovenia; locality: Dolnja Košana; minimumElevationInMeters: 435; maximumElevationInMeters: 435; decimalLatitude: 45.6646; decimalLongitude: 14.1350; **Event:** eventDate: 2012-07-27; habitat: grassland

#### Clubiona reclusa

O. P.-Cambridge, 1863

##### Materials

- **Type status:** Other material. **Occurrence:** recordedBy: Kuntner, Gregorič, Čandek; sex: 1 male; **Location:** locationID: CH17; country: Switzerland; locality: Engadin, Bivio; minimumElevationInMeters: 1780; maximumElevationInMeters: 1780; decimalLatitude: 46.4753; decimalLongitude: 9.6469; **Event:** eventDate: 2011-07-11; habitat: forest and river edge
- **Type status:** Other material. **Occurrence:** recordedBy: Kuntner, Gregorič, Čandek; sex: 1 male; **Location:** locationID: CH23; country: Switzerland; locality: Grison Alps, Alp Flix, Salategnas; minimumElevationInMeters: 1900; maximumElevationInMeters: 1900; decimalLatitude: 46.5141; decimalLongitude: 9.6448; **Event:** eventDate: 2011-07-12; habitat: forest opening, grass and shrubs
- **Type status:** Other material. **Occurrence:** recordedBy: Kuntner, Gregorič, Čandek; sex: 2 males; **Location:** locationID: CH30; country: Switzerland; locality: Grison Alps, Alp Flix - Lai Flix; minimumElevationInMeters: 1967; maximumElevationInMeters: 1967; decimalLatitude: 46.5358; decimalLongitude: 9.6409; **Event:** eventDate: 2011-07-16; habitat: next to alpine lake

#### Clubiona terrestris

Westring, 1851

##### Materials

- **Type status:** Other material. **Occurrence:** recordedBy: Kostanjšek, RTŠB 2011; sex: 1 male; **Location:** locationID: SI02; country: Slovenia; locality: Hrastovec; minimumElevationInMeters: 270; maximumElevationInMeters: 270; decimalLatitude: 46.5613; decimalLongitude: 15.7953; **Event:** eventDate: 2011-07-22; habitat: forest
- **Type status:** Other material. **Occurrence:** recordedBy: Čandek; sex: 1 male; **Location:** locationID: SI58; country: Slovenia; locality: Budanje; minimumElevationInMeters: 243; maximumElevationInMeters: 243; decimalLatitude: 45.8743; decimalLongitude: 13.9497; **Event:** eventDate: 2011-05-07; habitat: school and surroundings
- **Type status:** Other material. **Occurrence:** recordedBy: Čandek; sex: 1 female; **Location:** locationID: SI61; country: Slovenia; locality: Sekirišče; minimumElevationInMeters: 750; maximumElevationInMeters: 750; decimalLatitude: 45.8631; decimalLongitude: 14.5367; **Event:** eventDate: 2011-06-23/2012-06-21; habitat: house, grassland, overgrowth

#### Corinnidae

Karsch, 1880

#### Phrurolithus festivus

(C. L. Koch, 1835)

##### Materials

- **Type status:** Other material. **Occurrence:** recordedBy: Kostanjšek, RTŠB 2012; sex: 1 female; **Location:** locationID: SI06; country: Slovenia; locality: Čelje; minimumElevationInMeters: 630; maximumElevationInMeters: 630; decimalLatitude: 45.5981; decimalLongitude: 14.1465; **Event:** eventDate: 2012-07-21; habitat: grassland

#### Phrurolithus minimus

C. L. Koch, 1839

##### Materials

- **Type status:** Other material. **Occurrence:** recordedBy: Kostanjšek, RTŠB 2011; sex: 1 female; **Location:** locationID: SI15; country: Slovenia; locality: Apače; minimumElevationInMeters: 220; maximumElevationInMeters: 220; decimalLatitude: 46.6804; decimalLongitude: 15.8988; **Event:** eventDate: 2011-07-26; habitat: forest

#### Dictynidae

O. P.-Cambridge

#### Argenna subnigra

(O. P.-Cambridge, 1861)

##### Materials

- **Type status:** Other material. **Occurrence:** recordedBy: Kostanjšek, RTŠB 2012; sex: 1 female; **Location:** locationID: SI24; country: Slovenia; locality: Zagorje; minimumElevationInMeters: 550; maximumElevationInMeters: 550; decimalLatitude: 45.6413; decimalLongitude: 14.2384; **Event:** eventDate: 2012-07-26; habitat: grassland at river basin

#### Cicurina cicur

(Fabricius, 1793)

##### Materials

- **Type status:** Other material. **Occurrence:** recordedBy: Kostanjšek, RTŠB 2011; sex: 1 female; **Location:** locationID: SI15; country: Slovenia; locality: Apače; minimumElevationInMeters: 220; maximumElevationInMeters: 220; decimalLatitude: 46.6804; decimalLongitude: 15.8988; **Event:** eventDate: 2011-07-26; habitat: forest

#### Dictyna arundinacea

(Linnaeus, 1758)

##### Materials

- **Type status:** Other material. **Occurrence:** recordedBy: Kuntner, Gregorič, Čandek; sex: 1 female; **Location:** locationID: CH20; country: Switzerland; locality: Grison Alps, Alp Flix, Salategnas; minimumElevationInMeters: 1900; maximumElevationInMeters: 1900; decimalLatitude: 46.5181; decimalLongitude: 9.6480; **Event:** eventDate: 2011-07-12; habitat: grazed meadow
- **Type status:** Other material. **Occurrence:** recordedBy: Kuntner, Gregorič, Čandek; sex: 1 female; **Location:** locationID: CH21; country: Switzerland; locality: Grison Alps, Alp Flix, Salategnas; minimumElevationInMeters: 1970; maximumElevationInMeters: 1970; decimalLatitude: 46.5194; decimalLongitude: 9.6490; **Event:** eventDate: 2011-07-12; habitat: swamp grazed vegetation
- **Type status:** Other material. **Occurrence:** recordedBy: Kuntner, Gregorič, Čandek; sex: 1 female; **Location:** locationID: CH23; country: Switzerland; locality: Grison Alps, Alp Flix, Salategnas; minimumElevationInMeters: 1900; maximumElevationInMeters: 1900; decimalLatitude: 46.5141; decimalLongitude: 9.6448; **Event:** eventDate: 2011-07-12; habitat: forest opening, grass and shrubs

#### Dictyna civica

(Lucas, 1850)

##### Materials

- **Type status:** Other material. **Occurrence:** recordedBy: Kostanjšek, RTŠB 2011; sex: 1 female; **Location:** locationID: SI16; country: Slovenia; locality: Apače; minimumElevationInMeters: 215; maximumElevationInMeters: 215; decimalLatitude: 46.6954; decimalLongitude: 15.9087; **Event:** eventDate: 2011-07-26; habitat: house and surroundings

#### Dictyna uncinata

Thorell, 1856

##### Materials

- **Type status:** Other material. **Occurrence:** recordedBy: Čandek; sex: 1 female, 1 male; **Location:** locationID: SI37; country: Slovenia; locality: Podpeč, Brezovica; minimumElevationInMeters: 284; maximumElevationInMeters: 284; decimalLatitude: 45.9749; decimalLongitude: 14.4192; **Event:** eventDate: 2011-05-17; habitat: bridge

#### Lathys humilis

(Blackwall, 1855)

##### Materials

- **Type status:** Other material. **Occurrence:** recordedBy: Gregorič, Čandek, Kralj-Fišer; sex: 1 female; **Location:** locationID: SI56; country: Slovenia; locality: Dinaric Karst, Novelo; minimumElevationInMeters: 358; maximumElevationInMeters: 359; decimalLatitude: 45.8533; decimalLongitude: 13.6552; **Event:** eventDate: 2011-04-04/05-10; habitat: overgrowth

#### Lathys nielseni

(Schenkel, 1932)

##### Materials

- **Type status:** Other material. **Occurrence:** recordedBy: Kuntner, Gregorič, Čandek, Kralj-Fišer, Cheng; sex: 1 female; **Location:** locationID: SI41; country: Slovenia; locality: Socerb, Osp; minimumElevationInMeters: 116; maximumElevationInMeters: 116; decimalLatitude: 45.5819; decimalLongitude: 13.8558; **Event:** eventDate: 2012-06-07; habitat: trail from Socerb to Osp

#### Dysderidae

C. L. Koch, 1837

#### Dasumia canestrinii

(L. Koch, 1876)

##### Materials

- **Type status:** Other material. **Occurrence:** recordedBy: Kuntner, Lokovšek; sex: 1 male; **Location:** locationID: SI40; country: Slovenia; locality: Slavnik; minimumElevationInMeters: 816; maximumElevationInMeters: 816; decimalLatitude: 45.5499; decimalLongitude: 13.9619; **Event:** eventDate: 2010-08-26; habitat: grassland and forest
- **Type status:** Other material. **Occurrence:** recordedBy: Gregorič, Čandek, Kralj-Fišer; sex: 1 female, 2 males; **Location:** locationID: SI56; country: Slovenia; locality: Dinaric Karst, Novelo; minimumElevationInMeters: 358; maximumElevationInMeters: 359; decimalLatitude: 45.8533; decimalLongitude: 13.6552; **Event:** eventDate: 2011-04-04/05-10; habitat: overgrowth
- **Type status:** Other material. **Occurrence:** recordedBy: Čandek; sex: 1 male; **Location:** locationID: SI59; country: Slovenia; locality: Budanje; minimumElevationInMeters: 305; maximumElevationInMeters: 305; decimalLatitude: 45.8797; decimalLongitude: 13.9468; **Event:** eventDate: 2011-05-07; habitat: forest
- **Type status:** Other material. **Occurrence:** recordedBy: Gregorič, Čandek; sex: 1 female, 1 male; **Location:** locationID: SI70; country: Slovenia; locality: Dinaric Karst, Lokvice; minimumElevationInMeters: 200; maximumElevationInMeters: 200; decimalLatitude: 45.8614; decimalLongitude: 13.5903; **Event:** eventDate: 2011-05-10; habitat: forest

#### Dysdera adriatica

Kulczyn'ski, 1897

##### Materials

- **Type status:** Other material. **Occurrence:** recordedBy: Kostanjšek, RTŠB 2012; sex: 1 female, 1 male; **Location:** locationID: SI12; country: Slovenia; locality: Divača; minimumElevationInMeters: 660; maximumElevationInMeters: 660; decimalLatitude: 45.6788; decimalLongitude: 14.0437; **Event:** eventDate: 2012-07-22; habitat: forest

#### Dysdera erythrina

(Walckenaer, 1802)

##### Materials

- **Type status:** Other material. **Occurrence:** recordedBy: Kostanjšek, RTŠB 2012; sex: 1 female; **Location:** locationID: SI08; country: Slovenia; locality: Ribnica, Pivka; minimumElevationInMeters: 400; maximumElevationInMeters: 400; decimalLatitude: 45.6333; decimalLongitude: 14.1392; **Event:** eventDate: 2012-07-21; habitat: forest

#### Dysdera ninnii

Canestrini, 1868

##### Materials

- **Type status:** Other material. **Occurrence:** recordedBy: Gregorič, Čandek, Kralj-Fišer; sex: 4 females, 1 male; **Location:** locationID: SI56; country: Slovenia; locality: Dinaric Karst, Novelo; minimumElevationInMeters: 358; maximumElevationInMeters: 359; decimalLatitude: 45.8533; decimalLongitude: 13.6552; **Event:** eventDate: 2011-04-04/05-10; habitat: overgrowth

#### Filistatidae

Ausserer, 1867

#### Filistata insidiatrix

(Forsskĺl, 1775)

##### Materials

- **Type status:** Other material. **Occurrence:** recordedBy: Kuntner, Gregorič, Čandek, Kralj-Fišer, Cheng; sex: 2 females; **Location:** locationID: SI41; country: Slovenia; locality: Socerb, Osp; minimumElevationInMeters: 116; maximumElevationInMeters: 116; decimalLatitude: 45.5819; decimalLongitude: 13.8558; **Event:** eventDate: 2012-06-07; habitat: trail from Socerb to Osp

#### Gnaphosidae

Pocock, 1898

#### Aphantaulax cincta

(L. Koch, 1866)

##### Materials

- **Type status:** Other material. **Occurrence:** recordedBy: Gregorič, Čandek, Kralj-Fišer; sex: 1 male; **Location:** locationID: SI52; country: Slovenia; locality: Dinaric Karst, Griže; minimumElevationInMeters: 484; maximumElevationInMeters: 484; decimalLatitude: 45.7506; decimalLongitude: 13.9509; **Event:** eventDate: 2011-04-04/05-10; habitat: overgrowth

#### Callilepis schuszteri

(Herman, 1879)

##### Materials

- **Type status:** Other material. **Occurrence:** recordedBy: Kuntner, Gregorič, Čandek, Kralj-Fišer, Cheng; sex: 3 females, 2 males; **Location:** locationID: SI41; country: Slovenia; locality: Socerb, Osp; minimumElevationInMeters: 116; maximumElevationInMeters: 116; decimalLatitude: 45.5819; decimalLongitude: 13.8558; **Event:** eventDate: 2012-06-07; habitat: trail from Socerb to Osp

#### Drassodes lapidosus

(Walckenaer, 1802)

##### Materials

- **Type status:** Other material. **Occurrence:** recordedBy: Čandek; sex: 1 female; **Location:** locationID: SI58; country: Slovenia; locality: Budanje; minimumElevationInMeters: 243; maximumElevationInMeters: 243; decimalLatitude: 45.8743; decimalLongitude: 13.9497; **Event:** eventDate: 2011-05-07; habitat: school and surroundings
- **Type status:** Other material. **Occurrence:** recordedBy: Čandek; sex: 1 female; **Location:** locationID: SI61; country: Slovenia; locality: Sekirišče; minimumElevationInMeters: 750; maximumElevationInMeters: 750; decimalLatitude: 45.8631; decimalLongitude: 14.5367; **Event:** eventDate: 2011-06-23/2012-06-21; habitat: house, grassland, overgrowth

#### Drassodes pubescens

(Thorell, 1856)

##### Materials

- **Type status:** Other material. **Occurrence:** recordedBy: Kostanjšek, RTŠB 2012; sex: 1 female; **Location:** locationID: SI21; country: Slovenia; locality: Jurišče; minimumElevationInMeters: 730; maximumElevationInMeters: 730; decimalLatitude: 45.6735; decimalLongitude: 14.3093; **Event:** eventDate: 2012-07-23; habitat: overgrown grassland

#### Drassyllus villicus

(Thorell, 1875)

##### Materials

- **Type status:** Other material. **Occurrence:** recordedBy: Kuntner, Gregorič, Čandek, Kralj-Fišer, Cheng; sex: 2 females, 1 male; **Location:** locationID: SI41; country: Slovenia; locality: Socerb, Osp; minimumElevationInMeters: 116; maximumElevationInMeters: 116; decimalLatitude: 45.5819; decimalLongitude: 13.8558; **Event:** eventDate: 2012-06-07; habitat: trail from Socerb to Osp

#### Gnaphosa bicolor

(Hahn, 1833)

##### Materials

- **Type status:** Other material. **Occurrence:** recordedBy: Kostanjšek, RTŠB 2012; sex: 2 females; **Location:** locationID: SI12; country: Slovenia; locality: Divača; minimumElevationInMeters: 660; maximumElevationInMeters: 660; decimalLatitude: 45.6788; decimalLongitude: 14.0437; **Event:** eventDate: 2012-07-22; habitat: forest

#### Haplodrassus silvestris

(Blackwall, 1833)

##### Materials

- **Type status:** Other material. **Occurrence:** recordedBy: Gregorič, Čandek, Kralj-Fišer; sex: 1 female; **Location:** locationID: SI55; country: Slovenia; locality: Dinaric Karst, Lokvice; minimumElevationInMeters: 273; maximumElevationInMeters: 275; decimalLatitude: 45.8659; decimalLongitude: 13.6102; **Event:** eventDate: 2011-04-04/05-10; habitat: overgrowth

#### Micaria pulicaria

(Sundevall, 1831)

##### Materials

- **Type status:** Other material. **Occurrence:** recordedBy: Kostanjšek, RTŠB 2012; sex: 1 female; **Location:** locationID: SI24; country: Slovenia; locality: Zagorje; minimumElevationInMeters: 550; maximumElevationInMeters: 550; decimalLatitude: 45.6413; decimalLongitude: 14.2384; **Event:** eventDate: 2012-07-26; habitat: grassland at river basin

#### Micaria aenea

Thorell, 1871

##### Materials

- **Type status:** Other material. **Occurrence:** recordedBy: Kuntner, Gregorič, Čandek; sex: 1 female; **Location:** locationID: CH17; country: Switzerland; locality: Engadin, Bivio; minimumElevationInMeters: 1780; maximumElevationInMeters: 1780; decimalLatitude: 46.4753; decimalLongitude: 9.6469; **Event:** eventDate: 2011-07-11; habitat: forest and river edge

#### Nomisia extornata

(C. L. Koch, 1839)

##### Materials

- **Type status:** Other material. **Occurrence:** recordedBy: Kuntner, Gregorič, Čandek, Kralj-Fišer, Cheng; sex: 1 female; **Location:** locationID: SI41; country: Slovenia; locality: Socerb, Osp; minimumElevationInMeters: 116; maximumElevationInMeters: 116; decimalLatitude: 45.5819; decimalLongitude: 13.8558; **Event:** eventDate: 2012-06-07; habitat: trail from Socerb to Osp

#### Phaeocedus braccatus

(L. Koch, 1866)

##### Materials

- **Type status:** Other material. **Occurrence:** recordedBy: Čandek; sex: 1 male; **Location:** locationID: SI61; country: Slovenia; locality: Sekirišče; minimumElevationInMeters: 750; maximumElevationInMeters: 750; decimalLatitude: 45.8631; decimalLongitude: 14.5367; **Event:** eventDate: 2011-06-23/2012-06-21; habitat: house, grassland, overgrowth

#### Scotophaeus scutulatus

(L. Koch, 1866)

##### Materials

- **Type status:** Other material. **Occurrence:** recordedBy: Kuntner, Čandek; sex: 1 female, 1 male; **Location:** locationID: SI50; country: Slovenia; locality: Sp. Prapreče; minimumElevationInMeters: 351; maximumElevationInMeters: 351; decimalLatitude: 46.1620; decimalLongitude: 14.6933; **Event:** eventDate: 2010-08-03/2012-05-28; habitat: house and surroundings

#### Trachyzelotes pedestris

(C. L. Koch, 1837)

##### Materials

- **Type status:** Other material. **Occurrence:** recordedBy: Čandek; sex: 1 female; **Location:** locationID: SI46; country: Slovenia; locality: Šešče pri Preboldu; minimumElevationInMeters: 284; maximumElevationInMeters: 285; decimalLatitude: 46.2356; decimalLongitude: 15.1228; **Event:** eventDate: 2011-06-13/2012-06-22; habitat: house and surroundings

#### Zelotes apricorum

(L. Koch, 1876)

##### Materials

- **Type status:** Other material. **Occurrence:** recordedBy: Kostanjšek, RTŠB 2012; sex: 1 female; **Location:** locationID: SI09; country: Slovenia; locality: Divača; minimumElevationInMeters: 445; maximumElevationInMeters: 445; decimalLatitude: 45.6784; decimalLongitude: 13.9952; **Event:** eventDate: 2012-07-22; habitat: grassland

#### Zelotes latreillei

(Simon, 1878)

##### Materials

- **Type status:** Other material. **Occurrence:** recordedBy: Kostanjšek, RTŠB 2011; sex: 1 male; **Location:** locationID: SI05; country: Slovenia; locality: Sv. Jurij ob Ščavnici; minimumElevationInMeters: 190; maximumElevationInMeters: 190; decimalLatitude: 46.5509; decimalLongitude: 16.0451; **Event:** eventDate: 2011-07-22; habitat: overgrown river channel
- **Type status:** Other material. **Occurrence:** recordedBy: Čandek; sex: 1 female; **Location:** locationID: SI33; country: Slovenia; locality: Ljubljana, Biotechnical faculty; minimumElevationInMeters: 297; maximumElevationInMeters: 297; decimalLatitude: 46.0513; decimalLongitude: 14.4700; **Event:** eventDate: 2012-05-09; habitat: house and surroundings

#### Zelotes subterraneus

(C. L. Koch, 1833)

##### Materials

- **Type status:** Other material. **Occurrence:** recordedBy: Kuntner, Gregorič, Čandek; sex: 2 females; **Location:** locationID: CH27; country: Switzerland; locality: Grison Alps, road to Davos; minimumElevationInMeters: 1180; maximumElevationInMeters: 1180; decimalLatitude: 46.6808; decimalLongitude: 9.6557; **Event:** eventDate: 2011-07-15; habitat: roadside vegetation and forest edge

#### Hahniidae

Bertkau, 1878

#### Antistea elegans

(Blackwall, 1841)

##### Materials

- **Type status:** Other material. **Occurrence:** recordedBy: Kuntner, Gregorič, Čandek; sex: 3 females; **Location:** locationID: CH05; country: Switzerland; locality: Bernese Alps, Gasteretal; minimumElevationInMeters: 1380; maximumElevationInMeters: 1380; decimalLatitude: 46.4674; decimalLongitude: 7.6640; **Event:** eventDate: 2011-07-07; habitat: river vegetation

#### Hahnia difficilis

Harm, 1966

##### Materials

- **Type status:** Other material. **Occurrence:** recordedBy: Kuntner, Gregorič, Čandek; sex: 2 females; **Location:** locationID: CH03; country: Switzerland; locality: Bernese Alps, Gasteretal; minimumElevationInMeters: 1520; maximumElevationInMeters: 1520; decimalLatitude: 46.4498; decimalLongitude: 7.7135; **Event:** eventDate: 2011-07-07; habitat: spruce forest

#### Linyphiidae

Blackwall, 1859

#### Agnyphantes expunctus

(O. P.-Cambridge, 1875)

##### Materials

- **Type status:** Other material. **Occurrence:** recordedBy: Kuntner, Gregorič, Čandek; sex: 3 males; **Location:** locationID: CH16; country: Switzerland; locality: Engadin, Silvaplana; minimumElevationInMeters: 1930; maximumElevationInMeters: 1930; decimalLatitude: 46.4667; decimalLongitude: 9.7946; **Event:** eventDate: 2011-07-11; habitat: Larix and Pinus forest
- **Type status:** Other material. **Occurrence:** recordedBy: Kuntner, Gregorič, Čandek; sex: 6 females, 9 males; **Location:** locationID: CH23; country: Switzerland; locality: Grison Alps, Alp Flix, Salategnas; minimumElevationInMeters: 1900; maximumElevationInMeters: 1900; decimalLatitude: 46.5141; decimalLongitude: 9.6448; **Event:** eventDate: 2011-07-12; habitat: forest opening, grass and shrubs
- **Type status:** Other material. **Occurrence:** recordedBy: Kuntner, Gregorič, Čandek; sex: 5 females, 5 males; **Location:** locationID: CH32; country: Switzerland; locality: Grison Alps, Alp Flix, Salategnas; minimumElevationInMeters: 1955; maximumElevationInMeters: 1955; decimalLatitude: 46.5203; decimalLongitude: 9.6458; **Event:** eventDate: 2011-07-16; habitat: timberline forest, moss

#### Agyneta cauta

(O. P.-Cambridge, 1902)

##### Materials

- **Type status:** Other material. **Occurrence:** recordedBy: Kuntner, Gregorič, Čandek; sex: 1 female; **Location:** locationID: CH02; country: Switzerland; locality: Bernese Alps, Gasteretal; minimumElevationInMeters: 1698; maximumElevationInMeters: 1698; decimalLatitude: 46.4486; decimalLongitude: 7.7438; **Event:** eventDate: 2011-07-07; habitat: spruce thicket and grass
- **Type status:** Other material. **Occurrence:** recordedBy: Kuntner, Gregorič, Čandek; sex: 4 females; **Location:** locationID: CH09; country: Switzerland; locality: Pennine Alps, Mattertal; minimumElevationInMeters: 1447; maximumElevationInMeters: 1447; decimalLatitude: 46.0976; decimalLongitude: 7.7789; **Event:** eventDate: 2011-07-08; habitat: forest and meadow near river
- **Type status:** Other material. **Occurrence:** recordedBy: Kuntner, Gregorič, Čandek; sex: 1 female; **Location:** locationID: CH25; country: Switzerland; locality: Grison Alps, Alp Flix, Salategnas; minimumElevationInMeters: 1950; maximumElevationInMeters: 1950; decimalLatitude: 46.5159; decimalLongitude: 9.6496; **Event:** eventDate: 2011-07-12/16; habitat: meadow and shrubs at stream

#### Agyneta conigera

(O. P.-Cambridge, 1863)

##### Materials

- **Type status:** Other material. **Occurrence:** recordedBy: Kuntner, Gregorič, Čandek; sex: 1 male; **Location:** locationID: CH02; country: Switzerland; locality: Bernese Alps, Gasteretal; minimumElevationInMeters: 1698; maximumElevationInMeters: 1698; decimalLatitude: 46.4486; decimalLongitude: 7.7438; **Event:** eventDate: 2011-07-07; habitat: spruce thicket and grass

#### Bolyphantes alticeps

(Sundevall, 1833)

##### Materials

- **Type status:** Other material. **Occurrence:** recordedBy: Kuntner, Gregorič, Čandek; sex: 1 female; **Location:** locationID: CH31; country: Switzerland; locality: Grison Alps, Alp Flix - Lai Neir; minimumElevationInMeters: 1910; maximumElevationInMeters: 1910; decimalLatitude: 46.5343; decimalLongitude: 9.6375; **Event:** eventDate: 2011-07-16; habitat: lake and swamp around forest

#### Bolyphantes luteolus

(Blackwall, 1833)

##### Materials

- **Type status:** Other material. **Occurrence:** recordedBy: Kuntner, Gregorič, Čandek; sex: 1 female; **Location:** locationID: CH13; country: Switzerland; locality: Bernese Alps, Sustenpass; minimumElevationInMeters: 2040; maximumElevationInMeters: 2040; decimalLatitude: 46.7330; decimalLongitude: 8.4324; **Event:** eventDate: 2011-07-10; habitat: alpine grassland and shrubs
- **Type status:** Other material. **Occurrence:** recordedBy: Kuntner, Gregorič, Čandek; sex: 1 female; **Location:** locationID: CH31; country: Switzerland; locality: Grison Alps, Alp Flix - Lai Neir; minimumElevationInMeters: 1910; maximumElevationInMeters: 1910; decimalLatitude: 46.5343; decimalLongitude: 9.6375; **Event:** eventDate: 2011-07-16; habitat: lake and swamp around forest

#### Caracladus avicula

(L. Koch, 1869)

##### Materials

- **Type status:** Other material. **Occurrence:** recordedBy: Kuntner, Gregorič, Čandek; sex: 1 female; **Location:** locationID: CH24; country: Switzerland; locality: Grison Alps, Alp Flix, Salategnas; minimumElevationInMeters: 1830; maximumElevationInMeters: 1830; decimalLatitude: 46.5131; decimalLongitude: 9.6430; **Event:** eventDate: 2011-07-12; habitat: meadow and forest
- **Type status:** Other material. **Occurrence:** recordedBy: Kuntner, Gregorič, Čandek; sex: 4 females, 1 male; **Location:** locationID: CH32; country: Switzerland; locality: Grison Alps, Alp Flix, Salategnas; minimumElevationInMeters: 1955; maximumElevationInMeters: 1955; decimalLatitude: 46.5203; decimalLongitude: 9.6458; **Event:** eventDate: 2011-07-16; habitat: timberline forest, moss

#### Caracladus zamoniensis

Frick & Muff, 2009

##### Materials

- **Type status:** Other material. **Occurrence:** recordedBy: Kuntner, Gregorič, Čandek; sex: 1 female; **Location:** locationID: CH03; country: Switzerland; locality: Bernese Alps, Gasteretal; minimumElevationInMeters: 1520; maximumElevationInMeters: 1520; decimalLatitude: 46.4498; decimalLongitude: 7.7135; **Event:** eventDate: 2011-07-07; habitat: spruce forest
- **Type status:** Other material. **Occurrence:** recordedBy: Kuntner, Gregorič, Čandek; sex: 3 females; **Location:** locationID: CH18; country: Switzerland; locality: Grison Alps, Alp Flix, Salategnas; minimumElevationInMeters: 1900; maximumElevationInMeters: 1900; decimalLatitude: 46.5166; decimalLongitude: 9.6523; **Event:** eventDate: 2011-07-12/19; habitat: around house

#### Centromerus pabulator

(O. P.-Cambridge, 1875)

##### Materials

- **Type status:** Other material. **Occurrence:** recordedBy: Kuntner, Gregorič, Čandek; sex: 1 female; **Location:** locationID: CH12; country: Switzerland; locality: Bernese Alps, Nessental; minimumElevationInMeters: 930; maximumElevationInMeters: 930; decimalLatitude: 46.7213; decimalLongitude: 8.3039; **Event:** eventDate: 2011-07-10; habitat: grassland and loan trees
- **Type status:** Other material. **Occurrence:** recordedBy: Kuntner, Gregorič, Čandek; sex: 1 female; **Location:** locationID: CH21; country: Switzerland; locality: Grison Alps, Alp Flix, Salategnas; minimumElevationInMeters: 1970; maximumElevationInMeters: 1970; decimalLatitude: 46.5194; decimalLongitude: 9.6490; **Event:** eventDate: 2011-07-12; habitat: swamp grazed vegetation
- **Type status:** Other material. **Occurrence:** recordedBy: Kuntner, Gregorič, Čandek; sex: 3 females; **Location:** locationID: CH26; country: Switzerland; locality: Grison Alps, Alp Flix, Salategnas; minimumElevationInMeters: 1987; maximumElevationInMeters: 1987; decimalLatitude: 46.5166; decimalLongitude: 9.6516; **Event:** eventDate: 2011-07-14; habitat: grassland

#### Centromerus subalpinus

Lessert, 1907

##### Materials

- **Type status:** Other material. **Occurrence:** recordedBy: Kuntner, Gregorič, Čandek; sex: 1 female; **Location:** locationID: CH21; country: Switzerland; locality: Grison Alps, Alp Flix, Salategnas; minimumElevationInMeters: 1970; maximumElevationInMeters: 1970; decimalLatitude: 46.5194; decimalLongitude: 9.6490; **Event:** eventDate: 2011-07-12; habitat: swamp grazed vegetation
- **Type status:** Other material. **Occurrence:** recordedBy: Kuntner, Gregorič, Čandek; sex: 1 female; **Location:** locationID: CH31; country: Switzerland; locality: Grison Alps, Alp Flix - Lai Neir; minimumElevationInMeters: 1910; maximumElevationInMeters: 1910; decimalLatitude: 46.5343; decimalLongitude: 9.6375; **Event:** eventDate: 2011-07-16; habitat: lake and swamp around forest

#### Ceratinella brevipes

(Westring, 1851)

##### Materials

- **Type status:** Other material. **Occurrence:** recordedBy: Kuntner, Gregorič, Čandek; sex: 1 male; **Location:** locationID: CH32; country: Switzerland; locality: Grison Alps, Alp Flix, Salategnas; minimumElevationInMeters: 1955; maximumElevationInMeters: 1955; decimalLatitude: 46.5203; decimalLongitude: 9.6458; **Event:** eventDate: 2011-07-16; habitat: timberline forest, moss

#### Diplocephalus crassilobus

(Simon, 1884)

##### Materials

- **Type status:** Other material. **Occurrence:** recordedBy: Kostanjšek, RTŠB 2012; sex: 1 male; **Location:** locationID: SI17; country: Slovenia; locality: Novokračine; minimumElevationInMeters: 502; maximumElevationInMeters: 502; decimalLatitude: 45.4910; decimalLongitude: 14.3015; **Event:** eventDate: 2012-07-22; habitat: Novokrajska cave

#### Diplocephalus latifrons

(O. P.-Cambridge, 1863)

##### Materials

- **Type status:** Other material. **Occurrence:** recordedBy: Kuntner, Gregorič, Čandek; sex: 1 male; **Location:** locationID: CH03; country: Switzerland; locality: Bernese Alps, Gasteretal; minimumElevationInMeters: 1520; maximumElevationInMeters: 1520; decimalLatitude: 46.4498; decimalLongitude: 7.7135; **Event:** eventDate: 2011-07-07; habitat: spruce forest

#### Diplostyla concolor

(Wider, 1834)

##### Materials

- **Type status:** Other material. **Occurrence:** recordedBy: Kostanjšek, RTŠB 2011; sex: 1 female; **Location:** locationID: SI02; country: Slovenia; locality: Hrastovec; minimumElevationInMeters: 270; maximumElevationInMeters: 270; decimalLatitude: 46.5613; decimalLongitude: 15.7953; **Event:** eventDate: 2011-07-22; habitat: forest

#### Drapetisca socialis

(Sundevall, 1833)

##### Materials

- **Type status:** Other material. **Occurrence:** recordedBy: Kostanjšek, RTŠB 2011; sex: 1 female; **Location:** locationID: SI02; country: Slovenia; locality: Hrastovec; minimumElevationInMeters: 270; maximumElevationInMeters: 270; decimalLatitude: 46.5613; decimalLongitude: 15.7953; **Event:** eventDate: 2011-07-22; habitat: forest
- **Type status:** Other material. **Occurrence:** recordedBy: Kuntner, Gregorič, Lokovšek; sex: 2 females, 1 male; **Location:** locationID: SI39; country: Slovenia; locality: Primostek; minimumElevationInMeters: 157; maximumElevationInMeters: 157; decimalLatitude: 45.6299; decimalLongitude: 15.2997; **Event:** eventDate: 2010-08-24; habitat: grassland

#### Entelecara acuminata

(Wider, 1834)

##### Materials

- **Type status:** Other material. **Occurrence:** recordedBy: Kuntner, Gregorič, Čandek; sex: 1 female; **Location:** locationID: CH09; country: Switzerland; locality: Pennine Alps, Mattertal; minimumElevationInMeters: 1447; maximumElevationInMeters: 1447; decimalLatitude: 46.0976; decimalLongitude: 7.7789; **Event:** eventDate: 2011-07-08; habitat: forest and meadow near river
- **Type status:** Other material. **Occurrence:** recordedBy: Čandek; sex: 1 male; **Location:** locationID: SI61; country: Slovenia; locality: Sekirišče; minimumElevationInMeters: 750; maximumElevationInMeters: 750; decimalLatitude: 45.8631; decimalLongitude: 14.5367; **Event:** eventDate: 2011-06-23/2012-06-21; habitat: house, grassland, overgrowth

#### Erigone atra

Blackwall, 1833

##### Materials

- **Type status:** Other material. **Occurrence:** recordedBy: Kuntner, Gregorič, Čandek; sex: 1 male; **Location:** locationID: CH02; country: Switzerland; locality: Bernese Alps, Gasteretal; minimumElevationInMeters: 1698; maximumElevationInMeters: 1698; decimalLatitude: 46.4486; decimalLongitude: 7.7438; **Event:** eventDate: 2011-07-07; habitat: spruce thicket and grass
- **Type status:** Other material. **Occurrence:** recordedBy: Kuntner, Gregorič, Čandek; sex: 1 male; **Location:** locationID: CH19; country: Switzerland; locality: Grison Alps, Alp Flix, Salategnas; minimumElevationInMeters: 1910; maximumElevationInMeters: 1910; decimalLatitude: 46.5172; decimalLongitude: 9.6533; **Event:** eventDate: 2011-07-12; habitat: flat uncut grassland
- **Type status:** Other material. **Occurrence:** recordedBy: Kuntner, Gregorič, Čandek; sex: 1 male; **Location:** locationID: CH30; country: Switzerland; locality: Grison Alps, Alp Flix - Lai Flix; minimumElevationInMeters: 1967; maximumElevationInMeters: 1967; decimalLatitude: 46.5358; decimalLongitude: 9.6409; **Event:** eventDate: 2011-07-16; habitat: next to alpine lake

#### Erigone dentigera

O. P.-Cambridge, 1874

##### Materials

- **Type status:** Other material. **Occurrence:** recordedBy: Kuntner, Gregorič, Čandek; sex: 1 female; **Location:** locationID: CH25; country: Switzerland; locality: Grison Alps, Alp Flix, Salategnas; minimumElevationInMeters: 1950; maximumElevationInMeters: 1950; decimalLatitude: 46.5159; decimalLongitude: 9.6496; **Event:** eventDate: 2011-07-12/16; habitat: meadow and shrubs at stream

#### Erigone dentipalpis

(Wider, 1834)

##### Materials

- **Type status:** Other material. **Occurrence:** recordedBy: Kuntner, Gregorič, Čandek; sex: 1 male; **Location:** locationID: CH12; country: Switzerland; locality: Bernese Alps, Nessental; minimumElevationInMeters: 930; maximumElevationInMeters: 930; decimalLatitude: 46.7213; decimalLongitude: 8.3039; **Event:** eventDate: 2011-07-10; habitat: grassland and loan trees
- **Type status:** Other material. **Occurrence:** recordedBy: Kuntner, Gregorič, Čandek; sex: 2 males; **Location:** locationID: CH30; country: Switzerland; locality: Grison Alps, Alp Flix - Lai Flix; minimumElevationInMeters: 1967; maximumElevationInMeters: 1967; decimalLatitude: 46.5358; decimalLongitude: 9.6409; **Event:** eventDate: 2011-07-16; habitat: next to alpine lake

#### Erigone remota

L. Koch, 1869

##### Materials

- **Type status:** Other material. **Occurrence:** recordedBy: Kuntner, Gregorič, Čandek; sex: 1 male; **Location:** locationID: CH19; country: Switzerland; locality: Grison Alps, Alp Flix, Salategnas; minimumElevationInMeters: 1910; maximumElevationInMeters: 1910; decimalLatitude: 46.5172; decimalLongitude: 9.6533; **Event:** eventDate: 2011-07-12; habitat: flat uncut grassland

#### Erigone svenssoni

Holm, 1975

##### Materials

- **Type status:** Other material. **Occurrence:** recordedBy: Kuntner, Gregorič, Čandek; sex: 1 female; **Location:** locationID: CH05; country: Switzerland; locality: Bernese Alps, Gasteretal; minimumElevationInMeters: 1380; maximumElevationInMeters: 1380; decimalLatitude: 46.4674; decimalLongitude: 7.6640; **Event:** eventDate: 2011-07-07; habitat: river vegetation

#### Erigonella ignobilis

(O. P.-Cambridge, 1871)

##### Materials

- **Type status:** Other material. **Occurrence:** recordedBy: Kuntner, Gregorič, Čandek; sex: 2 females; **Location:** locationID: CH05; country: Switzerland; locality: Bernese Alps, Gasteretal; minimumElevationInMeters: 1380; maximumElevationInMeters: 1380; decimalLatitude: 46.4674; decimalLongitude: 7.6640; **Event:** eventDate: 2011-07-07; habitat: river vegetation

#### Floronia bucculenta

(Clerck, 1757)

##### Materials

- **Type status:** Other material. **Occurrence:** recordedBy: Kostanjšek, RTŠB 2011; sex: 1 female; **Location:** locationID: SI05; country: Slovenia; locality: Sv. Jurij ob Ščavnici; minimumElevationInMeters: 190; maximumElevationInMeters: 190; decimalLatitude: 46.5509; decimalLongitude: 16.0451; **Event:** eventDate: 2011-07-22; habitat: overgrown river channel

#### Frontinellina frutetorum

(C. L. Koch, 1834)

##### Materials

- **Type status:** Other material. **Occurrence:** recordedBy: Kuntner, Gregorič, Čandek, Kralj-Fišer, Cheng; sex: 3 females; **Location:** locationID: SI41; country: Slovenia; locality: Socerb, Osp; minimumElevationInMeters: 116; maximumElevationInMeters: 116; decimalLatitude: 45.5819; decimalLongitude: 13.8558; **Event:** eventDate: 2012-06-07; habitat: trail from Socerb to Osp
- **Type status:** Other material. **Occurrence:** recordedBy: Gregorič, Čandek, Kralj-Fišer; sex: 2 females; **Location:** locationID: SI52; country: Slovenia; locality: Dinaric Karst, Griže; minimumElevationInMeters: 484; maximumElevationInMeters: 484; decimalLatitude: 45.7506; decimalLongitude: 13.9509; **Event:** eventDate: 2011-04-04/05-10; habitat: overgrowth
- **Type status:** Other material. **Occurrence:** recordedBy: Gregorič, Čandek, Kralj-Fišer; sex: 5 females, 4 males; **Location:** locationID: SI55; country: Slovenia; locality: Dinaric Karst, Lokvice; minimumElevationInMeters: 273; maximumElevationInMeters: 275; decimalLatitude: 45.8659; decimalLongitude: 13.6102; **Event:** eventDate: 2011-04-04/05-10; habitat: overgrowth
- **Type status:** Other material. **Occurrence:** recordedBy: Gregorič, Čandek, Kralj-Fišer; sex: 2 females, 1 male; **Location:** locationID: SI56; country: Slovenia; locality: Dinaric Karst, Novelo; minimumElevationInMeters: 358; maximumElevationInMeters: 359; decimalLatitude: 45.8533; decimalLongitude: 13.6552; **Event:** eventDate: 2011-04-04/05-10; habitat: overgrowth

#### Gonatium hilare

(Thorell, 1875)

##### Materials

- **Type status:** Other material. **Occurrence:** recordedBy: Kuntner, Gregorič, Čandek, Kralj-Fišer, Cheng; sex: 1 male; **Location:** locationID: SI41; country: Slovenia; locality: Socerb, Osp; minimumElevationInMeters: 116; maximumElevationInMeters: 116; decimalLatitude: 45.5819; decimalLongitude: 13.8558; **Event:** eventDate: 2012-06-07; habitat: trail from Socerb to Osp

#### Gonatium rubellum

(Blackwall, 1841)

##### Materials

- **Type status:** Other material. **Occurrence:** recordedBy: Kuntner, Gregorič, Čandek; sex: 2 females; **Location:** locationID: CH23; country: Switzerland; locality: Grison Alps, Alp Flix, Salategnas; minimumElevationInMeters: 1900; maximumElevationInMeters: 1900; decimalLatitude: 46.5141; decimalLongitude: 9.6448; **Event:** eventDate: 2011-07-12; habitat: forest opening, grass and shrubs

#### Gonatium rubens

(Blackwall, 1833)

##### Materials

- **Type status:** Other material. **Occurrence:** recordedBy: Kuntner, Gregorič, Čandek; sex: 1 male; **Location:** locationID: CH13; country: Switzerland; locality: Bernese Alps, Sustenpass; minimumElevationInMeters: 2040; maximumElevationInMeters: 2040; decimalLatitude: 46.7330; decimalLongitude: 8.4324; **Event:** eventDate: 2011-07-10; habitat: alpine grassland and shrubs
- **Type status:** Other material. **Occurrence:** recordedBy: Kuntner, Gregorič, Čandek; sex: 1 female; **Location:** locationID: CH25; country: Switzerland; locality: Grison Alps, Alp Flix, Salategnas; minimumElevationInMeters: 1950; maximumElevationInMeters: 1950; decimalLatitude: 46.5159; decimalLongitude: 9.6496; **Event:** eventDate: 2011-07-12/16; habitat: meadow and shrubs at stream
- **Type status:** Other material. **Occurrence:** recordedBy: Kuntner, Gregorič, Čandek; sex: 2 females; **Location:** locationID: CH31; country: Switzerland; locality: Grison Alps, Alp Flix - Lai Neir; minimumElevationInMeters: 1910; maximumElevationInMeters: 1910; decimalLatitude: 46.5343; decimalLongitude: 9.6375; **Event:** eventDate: 2011-07-16; habitat: lake and swamp around forest

#### Improphantes nitidus

(Thorell, 1875)

##### Materials

- **Type status:** Other material. **Occurrence:** recordedBy: Kuntner, Gregorič, Čandek; sex: 1 female; **Location:** locationID: CH28; country: Switzerland; locality: Grison Alps, Alp Flix, Salategnas; minimumElevationInMeters: 1713; maximumElevationInMeters: 1713; decimalLatitude: 46.5165; decimalLongitude: 9.6387; **Event:** eventDate: 2011-07-15; habitat: forest edge

#### Incestophantes frigidus

(Simon, 1884)

##### Materials

- **Type status:** Other material. **Occurrence:** recordedBy: Kuntner, Gregorič, Čandek; sex: 1 female; **Location:** locationID: CH06; country: Switzerland; locality: Bernese Alps, Kandersteg; minimumElevationInMeters: 1677; maximumElevationInMeters: 1677; decimalLatitude: 46.5020; decimalLongitude: 7.6992; **Event:** eventDate: 2011-07-07; habitat: alpine meadow
- **Type status:** Other material. **Occurrence:** recordedBy: Kuntner, Gregorič, Čandek; sex: 2 females; **Location:** locationID: CH08; country: Switzerland; locality: Bernese Alps, Rothorn; minimumElevationInMeters: 2250; maximumElevationInMeters: 2250; decimalLatitude: 46.0183; decimalLongitude: 7.7687; **Event:** eventDate: 2011-07-08; habitat: grass, shrubs, spruce

#### Kaestneria dorsalis

(Wider, 1834)

##### Materials

- **Type status:** Other material. **Occurrence:** recordedBy: Kuntner, Gregorič, Lokovšek; sex: 1 female; **Location:** locationID: SI39; country: Slovenia; locality: Primostek; minimumElevationInMeters: 157; maximumElevationInMeters: 157; decimalLatitude: 45.6299; decimalLongitude: 15.2997; **Event:** eventDate: 2010-08-24; habitat: grassland

#### Lepthyphantes leprosus

(Ohlert, 1865)

##### Materials

- **Type status:** Other material. **Occurrence:** recordedBy: Kuntner, Gregorič, Čandek; sex: 1 female; **Location:** locationID: CH18; country: Switzerland; locality: Grison Alps, Alp Flix, Salategnas; minimumElevationInMeters: 1900; maximumElevationInMeters: 1900; decimalLatitude: 46.5166; decimalLongitude: 9.6523; **Event:** eventDate: 2011-07-12/19; habitat: around house
- **Type status:** Other material. **Occurrence:** recordedBy: Čandek; sex: 1 female; **Location:** locationID: SI34; country: Slovenia; locality: Ljubljana, castle; minimumElevationInMeters: 322; maximumElevationInMeters: 322; decimalLatitude: 46.0494; decimalLongitude: 14.5098; **Event:** eventDate: 2011-09-08; habitat: bark

#### Lepthyphantes minutus

(Blackwall, 1833)

##### Materials

- **Type status:** Other material. **Occurrence:** recordedBy: Kostanjšek, RTŠB 2011; sex: 1 female; **Location:** locationID: SI07; country: Slovenia; locality: Ljutomer; minimumElevationInMeters: 175; maximumElevationInMeters: 175; decimalLatitude: 46.5272; decimalLongitude: 16.2050; **Event:** eventDate: 2011-07-22; habitat: forest

#### Lepthyphantes nodifer

Simon, 1884

##### Materials

- **Type status:** Other material. **Occurrence:** recordedBy: Kuntner, Gregorič, Čandek; sex: 8 females, 1 male; **Location:** locationID: CH03; country: Switzerland; locality: Bernese Alps, Gasteretal; minimumElevationInMeters: 1520; maximumElevationInMeters: 1520; decimalLatitude: 46.4498; decimalLongitude: 7.7135; **Event:** eventDate: 2011-07-07; habitat: spruce forest
- **Type status:** Other material. **Occurrence:** recordedBy: Kuntner, Gregorič, Čandek; sex: 5 females; **Location:** locationID: CH17; country: Switzerland; locality: Engadin, Bivio; minimumElevationInMeters: 1780; maximumElevationInMeters: 1780; decimalLatitude: 46.4753; decimalLongitude: 9.6469; **Event:** eventDate: 2011-07-11; habitat: forest and river edge
- **Type status:** Other material. **Occurrence:** recordedBy: Kuntner, Gregorič, Čandek; sex: 2 females; **Location:** locationID: CH28; country: Switzerland; locality: Grison Alps, Alp Flix, Salategnas; minimumElevationInMeters: 1713; maximumElevationInMeters: 1713; decimalLatitude: 46.5165; decimalLongitude: 9.6387; **Event:** eventDate: 2011-07-15; habitat: forest edge

#### Leptorhoptrum robustum

(Westring, 1851)

##### Materials

- **Type status:** Other material. **Occurrence:** recordedBy: Kuntner, Gregorič, Čandek; sex: 1 male; **Location:** locationID: CH14; country: Switzerland; locality: Glarus Alps, Oberalppass; minimumElevationInMeters: 2040; maximumElevationInMeters: 2040; decimalLatitude: 46.6617; decimalLongitude: 8.6719; **Event:** eventDate: 2011-07-10; habitat: grassland and shrubs

#### Linyphia hortensis

Sundevall, 1830

##### Materials

- **Type status:** Other material. **Occurrence:** recordedBy: Kuntner, Gregorič, Čandek; sex: 1 female; **Location:** locationID: CH09; country: Switzerland; locality: Pennine Alps, Mattertal; minimumElevationInMeters: 1447; maximumElevationInMeters: 1447; decimalLatitude: 46.0976; decimalLongitude: 7.7789; **Event:** eventDate: 2011-07-08; habitat: forest and meadow near river
- **Type status:** Other material. **Occurrence:** recordedBy: Čandek; sex: 1 female; **Location:** locationID: SI59; country: Slovenia; locality: Budanje; minimumElevationInMeters: 305; maximumElevationInMeters: 305; decimalLatitude: 45.8797; decimalLongitude: 13.9468; **Event:** eventDate: 2011-05-07; habitat: forest

#### Linyphia triangularis

(Clerck, 1757)

##### Materials

- **Type status:** Other material. **Occurrence:** recordedBy: Kostanjšek, RTŠB 2011; sex: 1 male; **Location:** locationID: SI01; country: Slovenia; locality: Biš; minimumElevationInMeters: 225; maximumElevationInMeters: 225; decimalLatitude: 46.5374; decimalLongitude: 15.8963; **Event:** eventDate: 2011-07-22; habitat: forest
- **Type status:** Other material. **Occurrence:** recordedBy: Čandek; sex: 1 male; **Location:** locationID: SI30; country: Slovenia; locality: Ig, Iški Vintgar; minimumElevationInMeters: 371; maximumElevationInMeters: 371; decimalLatitude: 45.9090; decimalLongitude: 14.4955; **Event:** eventDate: 2011-07-31; habitat: forest
- **Type status:** Other material. **Occurrence:** recordedBy: Kuntner, Gregorič, Lokovšek; sex: 5 females; **Location:** locationID: SI39; country: Slovenia; locality: Primostek; minimumElevationInMeters: 157; maximumElevationInMeters: 157; decimalLatitude: 45.6299; decimalLongitude: 15.2997; **Event:** eventDate: 2010-08-24; habitat: grassland
- **Type status:** Other material. **Occurrence:** recordedBy: Čandek; sex: 1 male; **Location:** locationID: SI61; country: Slovenia; locality: Sekirišče; minimumElevationInMeters: 750; maximumElevationInMeters: 750; decimalLatitude: 45.8631; decimalLongitude: 14.5367; **Event:** eventDate: 2011-06-23/2012-06-21; habitat: house, grassland, overgrowth
- **Type status:** Other material. **Occurrence:** recordedBy: Kuntner; sex: 2 males; **Location:** locationID: SI69; country: Slovenia; locality: Lipalca; minimumElevationInMeters: 359; maximumElevationInMeters: 359; decimalLatitude: 46.0102; decimalLongitude: 14.3116; **Event:** eventDate: 2012-01-26; habitat: inside tree log

#### Macrargus rufus

(Wider, 1834)

##### Materials

- **Type status:** Other material. **Occurrence:** recordedBy: Kuntner, Gregorič, Čandek; sex: 2 females; **Location:** locationID: CH03; country: Switzerland; locality: Bernese Alps, Gasteretal; minimumElevationInMeters: 1520; maximumElevationInMeters: 1520; decimalLatitude: 46.4498; decimalLongitude: 7.7135; **Event:** eventDate: 2011-07-07; habitat: spruce forest

#### Mansuphantes fragilis

(Thorell, 1875)

##### Materials

- **Type status:** Other material. **Occurrence:** recordedBy: Kuntner, Gregorič, Čandek; sex: 1 female; **Location:** locationID: CH16; country: Switzerland; locality: Engadin, Silvaplana; minimumElevationInMeters: 1930; maximumElevationInMeters: 1930; decimalLatitude: 46.4667; decimalLongitude: 9.7946; **Event:** eventDate: 2011-07-11; habitat: Larix and Pinus forest
- **Type status:** Other material. **Occurrence:** recordedBy: Kuntner, Gregorič, Čandek; sex: 1 female; **Location:** locationID: CH28; country: Switzerland; locality: Grison Alps, Alp Flix, Salategnas; minimumElevationInMeters: 1713; maximumElevationInMeters: 1713; decimalLatitude: 46.5165; decimalLongitude: 9.6387; **Event:** eventDate: 2011-07-15; habitat: forest edge

#### Maso sundevalli

(Westring, 1851)

##### Materials

- **Type status:** Other material. **Occurrence:** recordedBy: Kuntner, Gregorič, Čandek; sex: 1 female, 1 male; **Location:** locationID: CH05; country: Switzerland; locality: Bernese Alps, Gasteretal; minimumElevationInMeters: 1380; maximumElevationInMeters: 1380; decimalLatitude: 46.4674; decimalLongitude: 7.6640; **Event:** eventDate: 2011-07-07; habitat: river vegetation
- **Type status:** Other material. **Occurrence:** recordedBy: Kuntner, Gregorič, Čandek; sex: 1 female, 2 males; **Location:** locationID: CH09; country: Switzerland; locality: Pennine Alps, Mattertal; minimumElevationInMeters: 1447; maximumElevationInMeters: 1447; decimalLatitude: 46.0976; decimalLongitude: 7.7789; **Event:** eventDate: 2011-07-08; habitat: forest and meadow near river
- **Type status:** Other material. **Occurrence:** recordedBy: Kuntner, Gregorič, Čandek; sex: 1 male; **Location:** locationID: CH17; country: Switzerland; locality: Engadin, Bivio; minimumElevationInMeters: 1780; maximumElevationInMeters: 1780; decimalLatitude: 46.4753; decimalLongitude: 9.6469; **Event:** eventDate: 2011-07-11; habitat: forest and river edge
- **Type status:** Other material. **Occurrence:** recordedBy: Kuntner, Gregorič, Čandek; sex: 1 female, 2 males; **Location:** locationID: CH28; country: Switzerland; locality: Grison Alps, Alp Flix, Salategnas; minimumElevationInMeters: 1713; maximumElevationInMeters: 1713; decimalLatitude: 46.5165; decimalLongitude: 9.6387; **Event:** eventDate: 2011-07-15; habitat: forest edge
- **Type status:** Other material. **Occurrence:** recordedBy: Kuntner, Gregorič, Čandek; sex: 1 female; **Location:** locationID: CH30; country: Switzerland; locality: Grison Alps, Alp Flix - Lai Flix; minimumElevationInMeters: 1967; maximumElevationInMeters: 1967; decimalLatitude: 46.5358; decimalLongitude: 9.6409; **Event:** eventDate: 2011-07-16; habitat: next to alpine lake
- **Type status:** Other material. **Occurrence:** recordedBy: Kuntner, Gregorič, Čandek; sex: 1 female, 2 males; **Location:** locationID: CH31; country: Switzerland; locality: Grison Alps, Alp Flix - Lai Neir; minimumElevationInMeters: 1910; maximumElevationInMeters: 1910; decimalLatitude: 46.5343; decimalLongitude: 9.6375; **Event:** eventDate: 2011-07-16; habitat: lake and swamp around forest
- **Type status:** Other material. **Occurrence:** recordedBy: Čandek; sex: 2 females, 3 males; **Location:** locationID: SI61; country: Slovenia; locality: Sekirišče; minimumElevationInMeters: 750; maximumElevationInMeters: 750; decimalLatitude: 45.8631; decimalLongitude: 14.5367; **Event:** eventDate: 2011-06-23/2012-06-21; habitat: house, grassland, overgrowth

#### Mecynargus foveatus

(Dahl, 1912)

##### Materials

- **Type status:** Other material. **Occurrence:** recordedBy: Kostanjšek, RTŠB 2012; sex: 1 female; **Location:** locationID: SI26; country: Slovenia; locality: Dolnja Košana; minimumElevationInMeters: 420; maximumElevationInMeters: 420; decimalLatitude: 45.6587; decimalLongitude: 14.1397; **Event:** eventDate: 2012-07-21; habitat: grassland

#### Megalepthyphantes collinus

(L. Koch, 1872)

##### Materials

- **Type status:** Other material. **Occurrence:** recordedBy: Kuntner, Lokovšek; sex: 1 female; **Location:** locationID: SI40; country: Slovenia; locality: Slavnik; minimumElevationInMeters: 816; maximumElevationInMeters: 816; decimalLatitude: 45.5499; decimalLongitude: 13.9619; **Event:** eventDate: 2010-08-26; habitat: grassland and forest

#### Megalepthyphantes nebulosus

(Sundevall, 1830)

##### Materials

- **Type status:** Other material. **Occurrence:** recordedBy: Kuntner, Gregorič, Čandek; sex: 1 female; **Location:** locationID: CH17; country: Switzerland; locality: Engadin, Bivio; minimumElevationInMeters: 1780; maximumElevationInMeters: 1780; decimalLatitude: 46.4753; decimalLongitude: 9.6469; **Event:** eventDate: 2011-07-11; habitat: forest and river edge
- **Type status:** Other material. **Occurrence:** recordedBy: Kuntner, Gregorič, Čandek; sex: 4 females; **Location:** locationID: CH23; country: Switzerland; locality: Grison Alps, Alp Flix, Salategnas; minimumElevationInMeters: 1900; maximumElevationInMeters: 1900; decimalLatitude: 46.5141; decimalLongitude: 9.6448; **Event:** eventDate: 2011-07-12; habitat: forest opening, grass and shrubs

#### Meioneta affinis

(Kulczyn'ski, 1898)

##### Materials

- **Type status:** Other material. **Occurrence:** recordedBy: Kuntner, Gregorič, Čandek; sex: 1 female, 1 male; **Location:** locationID: CH15; country: Switzerland; locality: Glarus Alps, near Affeier; minimumElevationInMeters: 817; maximumElevationInMeters: 817; decimalLatitude: 46.7606; decimalLongitude: 9.0933; **Event:** eventDate: 2011-07-10; habitat: meadow and forest

#### Meioneta alpica

(Tanasevitch, 2000)

##### Materials

- **Type status:** Other material. **Occurrence:** recordedBy: Kuntner, Gregorič, Čandek; sex: 1 male; **Location:** locationID: CH19; country: Switzerland; locality: Grison Alps, Alp Flix, Salategnas; minimumElevationInMeters: 1910; maximumElevationInMeters: 1910; decimalLatitude: 46.5172; decimalLongitude: 9.6533; **Event:** eventDate: 2011-07-12; habitat: flat uncut grassland

#### Meioneta fuscipalpa

(C. L. Koch, 1836)

##### Materials

- **Type status:** Other material. **Occurrence:** recordedBy: Kuntner, Gregorič, Čandek; sex: 1 female; **Location:** locationID: CH25; country: Switzerland; locality: Grison Alps, Alp Flix, Salategnas; minimumElevationInMeters: 1950; maximumElevationInMeters: 1950; decimalLatitude: 46.5159; decimalLongitude: 9.6496; **Event:** eventDate: 2011-07-12/16; habitat: meadow and shrubs at stream
- **Type status:** Other material. **Occurrence:** recordedBy: Kuntner, Gregorič, Čandek; sex: 1 female; **Location:** locationID: CH31; country: Switzerland; locality: Grison Alps, Alp Flix - Lai Neir; minimumElevationInMeters: 1910; maximumElevationInMeters: 1910; decimalLatitude: 46.5343; decimalLongitude: 9.6375; **Event:** eventDate: 2011-07-16; habitat: lake and swamp around forest
- **Type status:** Other material. **Occurrence:** recordedBy: Kostanjšek, RTŠB 2012; sex: 1 female; **Location:** locationID: SI26; country: Slovenia; locality: Dolnja Košana; minimumElevationInMeters: 420; maximumElevationInMeters: 420; decimalLatitude: 45.6587; decimalLongitude: 14.1397; **Event:** eventDate: 2012-07-21; habitat: grassland

#### Meioneta gulosa

(L. Koch, 1869)

##### Materials

- **Type status:** Other material. **Occurrence:** recordedBy: Kuntner, Gregorič, Čandek; sex: 1 male; **Location:** locationID: CH27; country: Switzerland; locality: Grison Alps, road to Davos; minimumElevationInMeters: 1180; maximumElevationInMeters: 1180; decimalLatitude: 46.6808; decimalLongitude: 9.6557; **Event:** eventDate: 2011-07-15; habitat: roadside vegetation and forest edge

#### Meioneta innotabilis

(O. P.-Cambridge, 1863)

##### Materials

- **Type status:** Other material. **Occurrence:** recordedBy: Kuntner, Gregorič, Čandek; sex: 1 male; **Location:** locationID: CH03; country: Switzerland; locality: Bernese Alps, Gasteretal; minimumElevationInMeters: 1520; maximumElevationInMeters: 1520; decimalLatitude: 46.4498; decimalLongitude: 7.7135; **Event:** eventDate: 2011-07-07; habitat: spruce forest

#### Meioneta orites

(Thorell, 1875)

##### Materials

- **Type status:** Other material. **Occurrence:** recordedBy: Kuntner, Gregorič, Čandek; sex: 1 female; **Location:** locationID: CH09; country: Switzerland; locality: Pennine Alps, Mattertal; minimumElevationInMeters: 1447; maximumElevationInMeters: 1447; decimalLatitude: 46.0976; decimalLongitude: 7.7789; **Event:** eventDate: 2011-07-08; habitat: forest and meadow near river
- **Type status:** Other material. **Occurrence:** recordedBy: Kuntner, Gregorič, Čandek; sex: 2 females; **Location:** locationID: CH25; country: Switzerland; locality: Grison Alps, Alp Flix, Salategnas; minimumElevationInMeters: 1950; maximumElevationInMeters: 1950; decimalLatitude: 46.5159; decimalLongitude: 9.6496; **Event:** eventDate: 2011-07-12/16; habitat: meadow and shrubs at stream

#### Meioneta rurestris

(C. L. Koch, 1836)

##### Materials

- **Type status:** Other material. **Occurrence:** recordedBy: Kuntner, Gregorič, Čandek; sex: 2 females, 1 male; **Location:** locationID: CH06; country: Switzerland; locality: Bernese Alps, Kandersteg; minimumElevationInMeters: 1677; maximumElevationInMeters: 1677; decimalLatitude: 46.5020; decimalLongitude: 7.6992; **Event:** eventDate: 2011-07-07; habitat: alpine meadow
- **Type status:** Other material. **Occurrence:** recordedBy: Kuntner, Gregorič, Čandek; sex: 1 female; **Location:** locationID: CH09; country: Switzerland; locality: Pennine Alps, Mattertal; minimumElevationInMeters: 1447; maximumElevationInMeters: 1447; decimalLatitude: 46.0976; decimalLongitude: 7.7789; **Event:** eventDate: 2011-07-08; habitat: forest and meadow near river
- **Type status:** Other material. **Occurrence:** recordedBy: Kuntner, Gregorič, Čandek; sex: 2 females; **Location:** locationID: CH25; country: Switzerland; locality: Grison Alps, Alp Flix, Salategnas; minimumElevationInMeters: 1950; maximumElevationInMeters: 1950; decimalLatitude: 46.5159; decimalLongitude: 9.6496; **Event:** eventDate: 2011-07-12/16; habitat: meadow and shrubs at stream
- **Type status:** Other material. **Occurrence:** recordedBy: Gregorič, Kuntner, Čandek; sex: 1 male; **Location:** locationID: SI48; country: Slovenia; locality: Ljubljana, center; minimumElevationInMeters: 291; maximumElevationInMeters: 291; decimalLatitude: 46.0434; decimalLongitude: 14.5041; **Event:** eventDate: 2011-05-24/2012-06-19; habitat: house
- **Type status:** Other material. **Occurrence:** recordedBy: Čandek; sex: 1 male; **Location:** locationID: SI61; country: Slovenia; locality: Sekirišče; minimumElevationInMeters: 750; maximumElevationInMeters: 750; decimalLatitude: 45.8631; decimalLongitude: 14.5367; **Event:** eventDate: 2011-06-23/2012-06-21; habitat: house, grassland, overgrowth

#### Meioneta saxatilis

(Blackwall, 1844)

##### Materials

- **Type status:** Other material. **Occurrence:** recordedBy: Kostanjšek, RTŠB 2012; sex: 1 female; **Location:** locationID: SI23; country: Slovenia; locality: Šembije; minimumElevationInMeters: 615; maximumElevationInMeters: 615; decimalLatitude: 45.6067; decimalLongitude: 14.2410; **Event:** eventDate: 2012-07-26; habitat: forest edge

#### Meioneta similis

(Kulczyn'ski, 1926)

##### Materials

- **Type status:** Other material. **Occurrence:** recordedBy: Kuntner, Gregorič, Čandek; sex: 1 female; **Location:** locationID: CH19; country: Switzerland; locality: Grison Alps, Alp Flix, Salategnas; minimumElevationInMeters: 1910; maximumElevationInMeters: 1910; decimalLatitude: 46.5172; decimalLongitude: 9.6533; **Event:** eventDate: 2011-07-12; habitat: flat uncut grassland

#### Meioneta simplicitarsis

(Simon, 1884)

##### Materials

- **Type status:** Other material. **Occurrence:** recordedBy: Kostanjšek, RTŠB 2012; sex: 5 females; **Location:** locationID: SI26; country: Slovenia; locality: Dolnja Košana; minimumElevationInMeters: 420; maximumElevationInMeters: 420; decimalLatitude: 45.6587; decimalLongitude: 14.1397; **Event:** eventDate: 2012-07-21; habitat: grassland

#### Mermessus trilobatus

(Emerton, 1882)

##### Materials

- **Type status:** Other material. **Occurrence:** recordedBy: Gregorič, Kuntner, Čandek; sex: 1 male; **Location:** locationID: SI48; country: Slovenia; locality: Ljubljana, center; minimumElevationInMeters: 291; maximumElevationInMeters: 291; decimalLatitude: 46.0434; decimalLongitude: 14.5041; **Event:** eventDate: 2011-05-24/2012-06-19; habitat: house
- **Type status:** Other material. **Occurrence:** recordedBy: Čandek; sex: 1 female; **Location:** locationID: SI61; country: Slovenia; locality: Sekirišče; minimumElevationInMeters: 750; maximumElevationInMeters: 750; decimalLatitude: 45.8631; decimalLongitude: 14.5367; **Event:** eventDate: 2011-06-23/2012-06-21; habitat: house, grassland, overgrowth

#### Metopobactrus prominulus

(O. P.-Cambridge, 1872)

##### Materials

- **Type status:** Other material. **Occurrence:** recordedBy: Kuntner, Gregorič, Čandek; sex: 2 females; **Location:** locationID: CH23; country: Switzerland; locality: Grison Alps, Alp Flix, Salategnas; minimumElevationInMeters: 1900; maximumElevationInMeters: 1900; decimalLatitude: 46.5141; decimalLongitude: 9.6448; **Event:** eventDate: 2011-07-12; habitat: forest opening, grass and shrubs

#### Micrargus alpinus

(O. P.-Cambridge, 1871)

##### Materials

- **Type status:** Other material. **Occurrence:** recordedBy: Kuntner, Gregorič, Čandek; sex: 1 female; **Location:** locationID: CH03; country: Switzerland; locality: Bernese Alps, Gasteretal; minimumElevationInMeters: 1520; maximumElevationInMeters: 1520; decimalLatitude: 46.4498; decimalLongitude: 7.7135; **Event:** eventDate: 2011-07-07; habitat: spruce forest
- **Type status:** Other material. **Occurrence:** recordedBy: Kuntner, Gregorič, Čandek; sex: 1 female; **Location:** locationID: CH28; country: Switzerland; locality: Grison Alps, Alp Flix, Salategnas; minimumElevationInMeters: 1713; maximumElevationInMeters: 1713; decimalLatitude: 46.5165; decimalLongitude: 9.6387; **Event:** eventDate: 2011-07-15; habitat: forest edge
- **Type status:** Other material. **Occurrence:** recordedBy: Kuntner, Gregorič, Čandek; sex: 1 female; **Location:** locationID: CH30; country: Switzerland; locality: Grison Alps, Alp Flix - Lai Flix; minimumElevationInMeters: 1967; maximumElevationInMeters: 1967; decimalLatitude: 46.5358; decimalLongitude: 9.6409; **Event:** eventDate: 2011-07-16; habitat: next to alpine lake
- **Type status:** Other material. **Occurrence:** recordedBy: Kuntner, Gregorič, Čandek; sex: 1 female; **Location:** locationID: CH31; country: Switzerland; locality: Grison Alps, Alp Flix - Lai Neir; minimumElevationInMeters: 1910; maximumElevationInMeters: 1910; decimalLatitude: 46.5343; decimalLongitude: 9.6375; **Event:** eventDate: 2011-07-16; habitat: lake and swamp around forest

#### Micrargus herbigradus

(Blackwall, 1854)

##### Materials

- **Type status:** Other material. **Occurrence:** recordedBy: Kostanjšek, RTŠB 2011; sex: 1 female; **Location:** locationID: SI15; country: Slovenia; locality: Apače; minimumElevationInMeters: 220; maximumElevationInMeters: 220; decimalLatitude: 46.6804; decimalLongitude: 15.8988; **Event:** eventDate: 2011-07-26; habitat: forest

#### Microctenonyx subitaneus

(O. P.-Cambridge, 1875)

##### Materials

- **Type status:** Other material. **Occurrence:** recordedBy: Kuntner, Gregorič, Čandek; sex: 1 male; **Location:** locationID: CH25; country: Switzerland; locality: Grison Alps, Alp Flix, Salategnas; minimumElevationInMeters: 1950; maximumElevationInMeters: 1950; decimalLatitude: 46.5159; decimalLongitude: 9.6496; **Event:** eventDate: 2011-07-12/16; habitat: meadow and shrubs at stream

#### Microlinyphia pusilla

(Sundevall, 1830)

##### Materials

- **Type status:** Other material. **Occurrence:** recordedBy: Kuntner, Gregorič, Čandek; sex: 1 female, 1 male; **Location:** locationID: CH05; country: Switzerland; locality: Bernese Alps, Gasteretal; minimumElevationInMeters: 1380; maximumElevationInMeters: 1380; decimalLatitude: 46.4674; decimalLongitude: 7.6640; **Event:** eventDate: 2011-07-07; habitat: river vegetation
- **Type status:** Other material. **Occurrence:** recordedBy: Kuntner, Gregorič, Čandek; sex: 1 female; **Location:** locationID: CH06; country: Switzerland; locality: Bernese Alps, Kandersteg; minimumElevationInMeters: 1677; maximumElevationInMeters: 1677; decimalLatitude: 46.5020; decimalLongitude: 7.6992; **Event:** eventDate: 2011-07-07; habitat: alpine meadow
- **Type status:** Other material. **Occurrence:** recordedBy: Kuntner, Gregorič, Čandek; sex: 1 female; **Location:** locationID: CH12; country: Switzerland; locality: Bernese Alps, Nessental; minimumElevationInMeters: 930; maximumElevationInMeters: 930; decimalLatitude: 46.7213; decimalLongitude: 8.3039; **Event:** eventDate: 2011-07-10; habitat: grassland and loan trees
- **Type status:** Other material. **Occurrence:** recordedBy: Kuntner, Gregorič, Čandek; sex: 1 female; **Location:** locationID: CH20; country: Switzerland; locality: Grison Alps, Alp Flix, Salategnas; minimumElevationInMeters: 1900; maximumElevationInMeters: 1900; decimalLatitude: 46.5181; decimalLongitude: 9.6480; **Event:** eventDate: 2011-07-12; habitat: grazed meadow
- **Type status:** Other material. **Occurrence:** recordedBy: Kuntner, Gregorič, Čandek; sex: 2 females; **Location:** locationID: CH23; country: Switzerland; locality: Grison Alps, Alp Flix, Salategnas; minimumElevationInMeters: 1900; maximumElevationInMeters: 1900; decimalLatitude: 46.5141; decimalLongitude: 9.6448; **Event:** eventDate: 2011-07-12; habitat: forest opening, grass and shrubs
- **Type status:** Other material. **Occurrence:** recordedBy: Kuntner, Gregorič, Čandek; sex: 1 female; **Location:** locationID: CH30; country: Switzerland; locality: Grison Alps, Alp Flix - Lai Flix; minimumElevationInMeters: 1967; maximumElevationInMeters: 1967; decimalLatitude: 46.5358; decimalLongitude: 9.6409; **Event:** eventDate: 2011-07-16; habitat: next to alpine lake

#### Minicia marginella

(Wider, 1834)

##### Materials

- **Type status:** Other material. **Occurrence:** recordedBy: Kuntner, Gregorič, Čandek; sex: 1 female, 1 male; **Location:** locationID: CH02; country: Switzerland; locality: Bernese Alps, Gasteretal; minimumElevationInMeters: 1698; maximumElevationInMeters: 1698; decimalLatitude: 46.4486; decimalLongitude: 7.7438; **Event:** eventDate: 2011-07-07; habitat: spruce thicket and grass
- **Type status:** Other material. **Occurrence:** recordedBy: Kuntner, Gregorič, Čandek; sex: 1 female; **Location:** locationID: CH13; country: Switzerland; locality: Bernese Alps, Sustenpass; minimumElevationInMeters: 2040; maximumElevationInMeters: 2040; decimalLatitude: 46.7330; decimalLongitude: 8.4324; **Event:** eventDate: 2011-07-10; habitat: alpine grassland and shrubs
- **Type status:** Other material. **Occurrence:** recordedBy: Gregorič, Čandek, Kralj-Fišer; sex: 4 females, 4 males; **Location:** locationID: SI52; country: Slovenia; locality: Dinaric Karst, Griže; minimumElevationInMeters: 484; maximumElevationInMeters: 484; decimalLatitude: 45.7506; decimalLongitude: 13.9509; **Event:** eventDate: 2011-04-04/05-10; habitat: overgrowth
- **Type status:** Other material. **Occurrence:** recordedBy: Gregorič, Čandek, Kralj-Fišer; sex: 1 female, 1 male; **Location:** locationID: SI56; country: Slovenia; locality: Dinaric Karst, Novelo; minimumElevationInMeters: 358; maximumElevationInMeters: 359; decimalLatitude: 45.8533; decimalLongitude: 13.6552; **Event:** eventDate: 2011-04-04/05-10; habitat: overgrowth
- **Type status:** Other material. **Occurrence:** recordedBy: Čandek; sex: 1 male; **Location:** locationID: SI61; country: Slovenia; locality: Sekirišče; minimumElevationInMeters: 750; maximumElevationInMeters: 750; decimalLatitude: 45.8631; decimalLongitude: 14.5367; **Event:** eventDate: 2011-06-23/2012-06-21; habitat: house, grassland, overgrowth

#### Minyriolus pusillus

(Wider, 1834)

##### Materials

- **Type status:** Other material. **Occurrence:** recordedBy: Kuntner, Gregorič, Čandek; sex: 7 females, 1 male; **Location:** locationID: CH03; country: Switzerland; locality: Bernese Alps, Gasteretal; minimumElevationInMeters: 1520; maximumElevationInMeters: 1520; decimalLatitude: 46.4498; decimalLongitude: 7.7135; **Event:** eventDate: 2011-07-07; habitat: spruce forest
- **Type status:** Other material. **Occurrence:** recordedBy: Kuntner, Gregorič, Čandek; sex: 3 females; **Location:** locationID: CH21; country: Switzerland; locality: Grison Alps, Alp Flix, Salategnas; minimumElevationInMeters: 1970; maximumElevationInMeters: 1970; decimalLatitude: 46.5194; decimalLongitude: 9.6490; **Event:** eventDate: 2011-07-12; habitat: swamp grazed vegetation
- **Type status:** Other material. **Occurrence:** recordedBy: Kuntner, Gregorič, Čandek; sex: 1 female; **Location:** locationID: CH26; country: Switzerland; locality: Grison Alps, Alp Flix, Salategnas; minimumElevationInMeters: 1987; maximumElevationInMeters: 1987; decimalLatitude: 46.5166; decimalLongitude: 9.6516; **Event:** eventDate: 2011-07-14; habitat: grassland
- **Type status:** Other material. **Occurrence:** recordedBy: Kuntner, Gregorič, Čandek; sex: 4 females; **Location:** locationID: CH31; country: Switzerland; locality: Grison Alps, Alp Flix - Lai Neir; minimumElevationInMeters: 1910; maximumElevationInMeters: 1910; decimalLatitude: 46.5343; decimalLongitude: 9.6375; **Event:** eventDate: 2011-07-16; habitat: lake and swamp around forest

#### Moebelia penicillata

(Westring, 1851)

##### Materials

- **Type status:** Other material. **Occurrence:** recordedBy: Čandek; sex: 4 males; **Location:** locationID: SI61; country: Slovenia; locality: Sekirišče; minimumElevationInMeters: 750; maximumElevationInMeters: 750; decimalLatitude: 45.8631; decimalLongitude: 14.5367; **Event:** eventDate: 2011-06-23/2012-06-21; habitat: house, grassland, overgrowth

#### Mughiphantes cornutus

(Schenkel, 1927)

##### Materials

- **Type status:** Other material. **Occurrence:** recordedBy: Kuntner, Gregorič, Čandek; sex: 1 female; **Location:** locationID: CH16; country: Switzerland; locality: Engadin, Silvaplana; minimumElevationInMeters: 1930; maximumElevationInMeters: 1930; decimalLatitude: 46.4667; decimalLongitude: 9.7946; **Event:** eventDate: 2011-07-11; habitat: Larix and Pinus forest
- **Type status:** Other material. **Occurrence:** recordedBy: Kuntner, Gregorič, Čandek; sex: 1 female, 1 male; **Location:** locationID: CH22; country: Switzerland; locality: Grison Alps, Alp Flix, Salategnas; minimumElevationInMeters: 1900; maximumElevationInMeters: 1900; decimalLatitude: 46.5152; decimalLongitude: 9.6466; **Event:** eventDate: 2011-07-12; habitat: forest ground
- **Type status:** Other material. **Occurrence:** recordedBy: Kuntner, Gregorič, Čandek; sex: 1 male; **Location:** locationID: CH32; country: Switzerland; locality: Grison Alps, Alp Flix, Salategnas; minimumElevationInMeters: 1955; maximumElevationInMeters: 1955; decimalLatitude: 46.5203; decimalLongitude: 9.6458; **Event:** eventDate: 2011-07-16; habitat: timberline forest, moss

#### Mughiphantes mughi

(Fickert, 1875)

##### Materials

- **Type status:** Other material. **Occurrence:** recordedBy: Kuntner, Gregorič, Čandek; sex: 1 male; **Location:** locationID: CH21; country: Switzerland; locality: Grison Alps, Alp Flix, Salategnas; minimumElevationInMeters: 1970; maximumElevationInMeters: 1970; decimalLatitude: 46.5194; decimalLongitude: 9.6490; **Event:** eventDate: 2011-07-12; habitat: swamp grazed vegetation
- **Type status:** Other material. **Occurrence:** recordedBy: Kuntner, Gregorič, Čandek; sex: 2 females; **Location:** locationID: CH23; country: Switzerland; locality: Grison Alps, Alp Flix, Salategnas; minimumElevationInMeters: 1900; maximumElevationInMeters: 1900; decimalLatitude: 46.5141; decimalLongitude: 9.6448; **Event:** eventDate: 2011-07-12; habitat: forest opening, grass and shrubs
- **Type status:** Other material. **Occurrence:** recordedBy: Kuntner, Gregorič, Čandek; sex: 1 male; **Location:** locationID: CH24; country: Switzerland; locality: Grison Alps, Alp Flix, Salategnas; minimumElevationInMeters: 1830; maximumElevationInMeters: 1830; decimalLatitude: 46.5131; decimalLongitude: 9.6430; **Event:** eventDate: 2011-07-12; habitat: meadow and forest
- **Type status:** Other material. **Occurrence:** recordedBy: Kuntner, Gregorič, Čandek; sex: 5 females, 2 males; **Location:** locationID: CH31; country: Switzerland; locality: Grison Alps, Alp Flix - Lai Neir; minimumElevationInMeters: 1910; maximumElevationInMeters: 1910; decimalLatitude: 46.5343; decimalLongitude: 9.6375; **Event:** eventDate: 2011-07-16; habitat: lake and swamp around forest
- **Type status:** Other material. **Occurrence:** recordedBy: Kuntner, Gregorič, Čandek; sex: 3 females, 1 male; **Location:** locationID: CH32; country: Switzerland; locality: Grison Alps, Alp Flix, Salategnas; minimumElevationInMeters: 1955; maximumElevationInMeters: 1955; decimalLatitude: 46.5203; decimalLongitude: 9.6458; **Event:** eventDate: 2011-07-16; habitat: timberline forest, moss

#### Nematogmus sanguinolentus

(Walckenaer, 1841)

##### Materials

- **Type status:** Other material. **Occurrence:** recordedBy: Kuntner, Gregorič, Čandek; sex: 1 female; **Location:** locationID: CH11; country: Switzerland; locality: Bernese Alps, Lake Brienz; minimumElevationInMeters: 600; maximumElevationInMeters: 600; decimalLatitude: 46.7569; decimalLongitude: 8.0107; **Event:** eventDate: 2011-07-10; habitat: meadows and forest
- **Type status:** Other material. **Occurrence:** recordedBy: Čandek; sex: 2 females, 1 male; **Location:** locationID: SI38; country: Slovenia; locality: Poreče; minimumElevationInMeters: 135; maximumElevationInMeters: 135; decimalLatitude: 45.8188; decimalLongitude: 13.9692; **Event:** eventDate: 2011-05-08; habitat: grassland
- **Type status:** Other material. **Occurrence:** recordedBy: Kuntner, Gregorič, Čandek, Kralj-Fišer, Cheng; sex: 1 female; **Location:** locationID: SI41; country: Slovenia; locality: Socerb, Osp; minimumElevationInMeters: 116; maximumElevationInMeters: 116; decimalLatitude: 45.5819; decimalLongitude: 13.8558; **Event:** eventDate: 2012-06-07; habitat: trail from Socerb to Osp
- **Type status:** Other material. **Occurrence:** recordedBy: Čandek; sex: 1 female; **Location:** locationID: SI43; country: Slovenia; locality: Vipava; minimumElevationInMeters: 114; maximumElevationInMeters: 114; decimalLatitude: 45.8282; decimalLongitude: 13.9594; **Event:** eventDate: 2011-05-08; habitat: grassland
- **Type status:** Other material. **Occurrence:** recordedBy: Gregorič, Čandek, Kralj-Fišer; sex: 2 females; **Location:** locationID: SI55; country: Slovenia; locality: Dinaric Karst, Lokvice; minimumElevationInMeters: 273; maximumElevationInMeters: 275; decimalLatitude: 45.8659; decimalLongitude: 13.6102; **Event:** eventDate: 2011-04-04/05-10; habitat: overgrowth
- **Type status:** Other material. **Occurrence:** recordedBy: Gregorič, Čandek, Kralj-Fišer; sex: 1 female; **Location:** locationID: SI56; country: Slovenia; locality: Dinaric Karst, Novelo; minimumElevationInMeters: 358; maximumElevationInMeters: 359; decimalLatitude: 45.8533; decimalLongitude: 13.6552; **Event:** eventDate: 2011-04-04/05-10; habitat: overgrowth
- **Type status:** Other material. **Occurrence:** recordedBy: Gregorič, Čandek; sex: 9 females, 4 males; **Location:** locationID: SI57; country: Slovenia; locality: Dinaric Karst, Novelo; minimumElevationInMeters: 325; maximumElevationInMeters: 325; decimalLatitude: 45.8482; decimalLongitude: 13.6584; **Event:** eventDate: 2011-05-10; habitat: grassland
- **Type status:** Other material. **Occurrence:** recordedBy: Čandek; sex: 1 male; **Location:** locationID: SI60; country: Slovenia; locality: Budanje; minimumElevationInMeters: 295; maximumElevationInMeters: 295; decimalLatitude: 45.8799; decimalLongitude: 13.9459; **Event:** eventDate: 2011-05-07; habitat: forest clearing
- **Type status:** Other material. **Occurrence:** recordedBy: Čandek; sex: 1 male; **Location:** locationID: SI61; country: Slovenia; locality: Sekirišče; minimumElevationInMeters: 750; maximumElevationInMeters: 750; decimalLatitude: 45.8631; decimalLongitude: 14.5367; **Event:** eventDate: 2011-06-23/2012-06-21; habitat: house, grassland, overgrowth

#### Neriene clathrata

(Sundevall, 1830)

##### Materials

- **Type status:** Other material. **Occurrence:** recordedBy: Kuntner, Gregorič, Čandek; sex: 1 female; **Location:** locationID: CH09; country: Switzerland; locality: Pennine Alps, Mattertal; minimumElevationInMeters: 1447; maximumElevationInMeters: 1447; decimalLatitude: 46.0976; decimalLongitude: 7.7789; **Event:** eventDate: 2011-07-08; habitat: forest and meadow near river
- **Type status:** Other material. **Occurrence:** recordedBy: Kuntner, Gregorič, Čandek; sex: 1 female; **Location:** locationID: CH15; country: Switzerland; locality: Glarus Alps, near Affeier; minimumElevationInMeters: 817; maximumElevationInMeters: 817; decimalLatitude: 46.7606; decimalLongitude: 9.0933; **Event:** eventDate: 2011-07-10; habitat: meadow and forest
- **Type status:** Other material. **Occurrence:** recordedBy: Kuntner, Gregorič, Čandek; sex: 1 male; **Location:** locationID: CH23; country: Switzerland; locality: Grison Alps, Alp Flix, Salategnas; minimumElevationInMeters: 1900; maximumElevationInMeters: 1900; decimalLatitude: 46.5141; decimalLongitude: 9.6448; **Event:** eventDate: 2011-07-12; habitat: forest opening, grass and shrubs
- **Type status:** Other material. **Occurrence:** recordedBy: Kostanjšek, RTŠB 2011; sex: 1 female; **Location:** locationID: SI14; country: Slovenia; locality: Spodnji Velovlek; minimumElevationInMeters: 225; maximumElevationInMeters: 225; decimalLatitude: 46.4768; decimalLongitude: 15.9316; **Event:** eventDate: 2011-07-25; habitat: forest
- **Type status:** Other material. **Occurrence:** recordedBy: Gregorič, Čandek, Kralj-Fišer; sex: 1 female, 1 male; **Location:** locationID: SI56; country: Slovenia; locality: Dinaric Karst, Novelo; minimumElevationInMeters: 358; maximumElevationInMeters: 359; decimalLatitude: 45.8533; decimalLongitude: 13.6552; **Event:** eventDate: 2011-04-04/05-10; habitat: overgrowth

#### Neriene furtiva

(O. P.-Cambridge, 1871)

##### Materials

- **Type status:** Other material. **Occurrence:** recordedBy: Kuntner, Gregorič, Čandek, Kralj-Fišer, Cheng; sex: 1 female; **Location:** locationID: SI41; country: Slovenia; locality: Socerb, Osp; minimumElevationInMeters: 116; maximumElevationInMeters: 116; decimalLatitude: 45.5819; decimalLongitude: 13.8558; **Event:** eventDate: 2012-06-07; habitat: trail from Socerb to Osp
- **Type status:** Other material. **Occurrence:** recordedBy: Gregorič, Čandek, Kralj-Fišer; sex: 1 female; **Location:** locationID: SI52; country: Slovenia; locality: Dinaric Karst, Griže; minimumElevationInMeters: 484; maximumElevationInMeters: 484; decimalLatitude: 45.7506; decimalLongitude: 13.9509; **Event:** eventDate: 2011-04-04/05-10; habitat: overgrowth
- **Type status:** Other material. **Occurrence:** recordedBy: Gregorič, Čandek; sex: 7 females, 4 males; **Location:** locationID: SI53; country: Slovenia; locality: Dinaric Karst, Griže; minimumElevationInMeters: 434; maximumElevationInMeters: 434; decimalLatitude: 45.7548; decimalLongitude: 13.9495; **Event:** eventDate: 2011-05-10/2011-06-21; habitat: grassland
- **Type status:** Other material. **Occurrence:** recordedBy: Gregorič, Čandek, Kralj-Fišer; sex: 4 females, 1 male; **Location:** locationID: SI55; country: Slovenia; locality: Dinaric Karst, Lokvice; minimumElevationInMeters: 273; maximumElevationInMeters: 275; decimalLatitude: 45.8659; decimalLongitude: 13.6102; **Event:** eventDate: 2011-04-04/05-10; habitat: overgrowth
- **Type status:** Other material. **Occurrence:** recordedBy: Gregorič, Čandek, Kralj-Fišer; sex: 1 male; **Location:** locationID: SI56; country: Slovenia; locality: Dinaric Karst, Novelo; minimumElevationInMeters: 358; maximumElevationInMeters: 359; decimalLatitude: 45.8533; decimalLongitude: 13.6552; **Event:** eventDate: 2011-04-04/05-10; habitat: overgrowth
- **Type status:** Other material. **Occurrence:** recordedBy: Gregorič, Čandek; sex: 1 female, 1 male; **Location:** locationID: SI57; country: Slovenia; locality: Dinaric Karst, Novelo; minimumElevationInMeters: 325; maximumElevationInMeters: 325; decimalLatitude: 45.8482; decimalLongitude: 13.6584; **Event:** eventDate: 2011-05-10; habitat: grassland

#### Neriene peltata

(Wider, 1834)

##### Materials

- **Type status:** Other material. **Occurrence:** recordedBy: Kuntner, Gregorič, Čandek; sex: 2 females; **Location:** locationID: CH02; country: Switzerland; locality: Bernese Alps, Gasteretal; minimumElevationInMeters: 1698; maximumElevationInMeters: 1698; decimalLatitude: 46.4486; decimalLongitude: 7.7438; **Event:** eventDate: 2011-07-07; habitat: spruce thicket and grass

#### Neriene radiata

(Walckenaer, 1841)

##### Materials

- **Type status:** Other material. **Occurrence:** recordedBy: Kuntner, Gregorič, Čandek; sex: 1 female; **Location:** locationID: CH27; country: Switzerland; locality: Grison Alps, road to Davos; minimumElevationInMeters: 1180; maximumElevationInMeters: 1180; decimalLatitude: 46.6808; decimalLongitude: 9.6557; **Event:** eventDate: 2011-07-15; habitat: roadside vegetation and forest edge
- **Type status:** Other material. **Occurrence:** recordedBy: Kuntner, Gregorič, Čandek, Kralj-Fišer, Cheng; sex: 1 female, 1 male; **Location:** locationID: SI41; country: Slovenia; locality: Socerb, Osp; minimumElevationInMeters: 116; maximumElevationInMeters: 116; decimalLatitude: 45.5819; decimalLongitude: 13.8558; **Event:** eventDate: 2012-06-07; habitat: trail from Socerb to Osp

#### Obscuriphantes obscurus

(Blackwall, 1841)

##### Materials

- **Type status:** Other material. **Occurrence:** recordedBy: Kuntner, Gregorič, Čandek; sex: 1 female; **Location:** locationID: CH03; country: Switzerland; locality: Bernese Alps, Gasteretal; minimumElevationInMeters: 1520; maximumElevationInMeters: 1520; decimalLatitude: 46.4498; decimalLongitude: 7.7135; **Event:** eventDate: 2011-07-07; habitat: spruce forest
- **Type status:** Other material. **Occurrence:** recordedBy: Kuntner, Gregorič, Čandek; sex: 1 female; **Location:** locationID: CH23; country: Switzerland; locality: Grison Alps, Alp Flix, Salategnas; minimumElevationInMeters: 1900; maximumElevationInMeters: 1900; decimalLatitude: 46.5141; decimalLongitude: 9.6448; **Event:** eventDate: 2011-07-12; habitat: forest opening, grass and shrubs

#### Oedothorax gibbifer

(Kulczyn'ski, 1882)

##### Materials

- **Type status:** Other material. **Occurrence:** recordedBy: Kuntner, Gregorič, Čandek; sex: 1 female; **Location:** locationID: CH25; country: Switzerland; locality: Grison Alps, Alp Flix, Salategnas; minimumElevationInMeters: 1950; maximumElevationInMeters: 1950; decimalLatitude: 46.5159; decimalLongitude: 9.6496; **Event:** eventDate: 2011-07-12/16; habitat: meadow and shrubs at stream

#### Ostearius melanopygius

(O. P.-Cambridge, 1879)

##### Materials

- **Type status:** Other material. **Occurrence:** recordedBy: Čandek; sex: 1 female; **Location:** locationID: SI46; country: Slovenia; locality: Šešče pri Preboldu; minimumElevationInMeters: 284; maximumElevationInMeters: 285; decimalLatitude: 46.2356; decimalLongitude: 15.1228; **Event:** eventDate: 2011-06-13/2012-06-22; habitat: house and surroundings

#### Palliduphantes pallidus

(O. P.-Cambridge, 1871)

##### Materials

- **Type status:** Other material. **Occurrence:** recordedBy: Kuntner, Gregorič, Čandek; sex: 1 male; **Location:** locationID: CH03; country: Switzerland; locality: Bernese Alps, Gasteretal; minimumElevationInMeters: 1520; maximumElevationInMeters: 1520; decimalLatitude: 46.4498; decimalLongitude: 7.7135; **Event:** eventDate: 2011-07-07; habitat: spruce forest
- **Type status:** Other material. **Occurrence:** recordedBy: Kuntner, Gregorič, Čandek; sex: 1 female; **Location:** locationID: CH28; country: Switzerland; locality: Grison Alps, Alp Flix, Salategnas; minimumElevationInMeters: 1713; maximumElevationInMeters: 1713; decimalLatitude: 46.5165; decimalLongitude: 9.6387; **Event:** eventDate: 2011-07-15; habitat: forest edge

#### Panamomops tauricornis

(Simon, 1881)

##### Materials

- **Type status:** Other material. **Occurrence:** recordedBy: Kuntner, Gregorič, Čandek; sex: 11 females, 8 males; **Location:** locationID: CH26; country: Switzerland; locality: Grison Alps, Alp Flix, Salategnas; minimumElevationInMeters: 1987; maximumElevationInMeters: 1987; decimalLatitude: 46.5166; decimalLongitude: 9.6516; **Event:** eventDate: 2011-07-14; habitat: grassland

#### Pelecopsis elongata

(Wider, 1834)

##### Materials

- **Type status:** Other material. **Occurrence:** recordedBy: Čandek; sex: 1 female; **Location:** locationID: SI61; country: Slovenia; locality: Sekirišče; minimumElevationInMeters: 750; maximumElevationInMeters: 750; decimalLatitude: 45.8631; decimalLongitude: 14.5367; **Event:** eventDate: 2011-06-23/2012-06-21; habitat: house, grassland, overgrowth

#### Pelecopsis mengei

(Simon, 1884)

##### Materials

- **Type status:** Other material. **Occurrence:** recordedBy: Kuntner, Gregorič, Čandek; sex: 1 female; **Location:** locationID: CH22; country: Switzerland; locality: Grison Alps, Alp Flix, Salategnas; minimumElevationInMeters: 1900; maximumElevationInMeters: 1900; decimalLatitude: 46.5152; decimalLongitude: 9.6466; **Event:** eventDate: 2011-07-12; habitat: forest ground
- **Type status:** Other material. **Occurrence:** recordedBy: Kuntner, Gregorič, Čandek; sex: 1 female; **Location:** locationID: CH24; country: Switzerland; locality: Grison Alps, Alp Flix, Salategnas; minimumElevationInMeters: 1830; maximumElevationInMeters: 1830; decimalLatitude: 46.5131; decimalLongitude: 9.6430; **Event:** eventDate: 2011-07-12; habitat: meadow and forest

#### Pelecopsis radicicola

(L. Koch, 1872)

##### Materials

- **Type status:** Other material. **Occurrence:** recordedBy: Kuntner, Gregorič, Čandek; sex: 1 female; **Location:** locationID: CH12; country: Switzerland; locality: Bernese Alps, Nessental; minimumElevationInMeters: 930; maximumElevationInMeters: 930; decimalLatitude: 46.7213; decimalLongitude: 8.3039; **Event:** eventDate: 2011-07-10; habitat: grassland and loan trees
- **Type status:** Other material. **Occurrence:** recordedBy: Kuntner, Gregorič, Čandek; sex: 4 females, 2 males; **Location:** locationID: CH16; country: Switzerland; locality: Engadin, Silvaplana; minimumElevationInMeters: 1930; maximumElevationInMeters: 1930; decimalLatitude: 46.4667; decimalLongitude: 9.7946; **Event:** eventDate: 2011-07-11; habitat: Larix and Pinus forest
- **Type status:** Other material. **Occurrence:** recordedBy: Kuntner, Gregorič, Čandek; sex: 1 male; **Location:** locationID: CH21; country: Switzerland; locality: Grison Alps, Alp Flix, Salategnas; minimumElevationInMeters: 1970; maximumElevationInMeters: 1970; decimalLatitude: 46.5194; decimalLongitude: 9.6490; **Event:** eventDate: 2011-07-12; habitat: swamp grazed vegetation
- **Type status:** Other material. **Occurrence:** recordedBy: Kuntner, Gregorič, Čandek; sex: 1 female, 3 males; **Location:** locationID: CH22; country: Switzerland; locality: Grison Alps, Alp Flix, Salategnas; minimumElevationInMeters: 1900; maximumElevationInMeters: 1900; decimalLatitude: 46.5152; decimalLongitude: 9.6466; **Event:** eventDate: 2011-07-12; habitat: forest ground
- **Type status:** Other material. **Occurrence:** recordedBy: Kuntner, Gregorič, Čandek; sex: 9 females; **Location:** locationID: CH23; country: Switzerland; locality: Grison Alps, Alp Flix, Salategnas; minimumElevationInMeters: 1900; maximumElevationInMeters: 1900; decimalLatitude: 46.5141; decimalLongitude: 9.6448; **Event:** eventDate: 2011-07-12; habitat: forest opening, grass and shrubs
- **Type status:** Other material. **Occurrence:** recordedBy: Kuntner, Gregorič, Čandek; sex: 1 female; **Location:** locationID: CH24; country: Switzerland; locality: Grison Alps, Alp Flix, Salategnas; minimumElevationInMeters: 1830; maximumElevationInMeters: 1830; decimalLatitude: 46.5131; decimalLongitude: 9.6430; **Event:** eventDate: 2011-07-12; habitat: meadow and forest
- **Type status:** Other material. **Occurrence:** recordedBy: Kuntner, Gregorič, Čandek; sex: 5 females, 1 male; **Location:** locationID: CH28; country: Switzerland; locality: Grison Alps, Alp Flix, Salategnas; minimumElevationInMeters: 1713; maximumElevationInMeters: 1713; decimalLatitude: 46.5165; decimalLongitude: 9.6387; **Event:** eventDate: 2011-07-15; habitat: forest edge
- **Type status:** Other material. **Occurrence:** recordedBy: Kuntner, Gregorič, Čandek; sex: 5 females, 1 male; **Location:** locationID: CH30; country: Switzerland; locality: Grison Alps, Alp Flix - Lai Flix; minimumElevationInMeters: 1967; maximumElevationInMeters: 1967; decimalLatitude: 46.5358; decimalLongitude: 9.6409; **Event:** eventDate: 2011-07-16; habitat: next to alpine lake
- **Type status:** Other material. **Occurrence:** recordedBy: Kuntner, Gregorič, Čandek; sex: 6 females, 1 male; **Location:** locationID: CH31; country: Switzerland; locality: Grison Alps, Alp Flix - Lai Neir; minimumElevationInMeters: 1910; maximumElevationInMeters: 1910; decimalLatitude: 46.5343; decimalLongitude: 9.6375; **Event:** eventDate: 2011-07-16; habitat: lake and swamp around forest
- **Type status:** Other material. **Occurrence:** recordedBy: Kuntner, Gregorič, Čandek; sex: 1 female; **Location:** locationID: CH32; country: Switzerland; locality: Grison Alps, Alp Flix, Salategnas; minimumElevationInMeters: 1955; maximumElevationInMeters: 1955; decimalLatitude: 46.5203; decimalLongitude: 9.6458; **Event:** eventDate: 2011-07-16; habitat: timberline forest, moss

#### Pityohyphantes phrygianus

(C. L. Koch, 1836)

##### Materials

- **Type status:** Other material. **Occurrence:** recordedBy: Kuntner, Gregorič, Čandek; sex: 1 female, 1 male; **Location:** locationID: CH20; country: Switzerland; locality: Grison Alps, Alp Flix, Salategnas; minimumElevationInMeters: 1900; maximumElevationInMeters: 1900; decimalLatitude: 46.5181; decimalLongitude: 9.6480; **Event:** eventDate: 2011-07-12; habitat: grazed meadow
- **Type status:** Other material. **Occurrence:** recordedBy: Kuntner, Gregorič, Čandek; sex: 5 females, 2 males; **Location:** locationID: CH23; country: Switzerland; locality: Grison Alps, Alp Flix, Salategnas; minimumElevationInMeters: 1900; maximumElevationInMeters: 1900; decimalLatitude: 46.5141; decimalLongitude: 9.6448; **Event:** eventDate: 2011-07-12; habitat: forest opening, grass and shrubs

#### Pocadicnemis juncea

Locket & Millidge, 1953

##### Materials

- **Type status:** Other material. **Occurrence:** recordedBy: Kuntner, Gregorič, Čandek; sex: 1 female; **Location:** locationID: CH06; country: Switzerland; locality: Bernese Alps, Kandersteg; minimumElevationInMeters: 1677; maximumElevationInMeters: 1677; decimalLatitude: 46.5020; decimalLongitude: 7.6992; **Event:** eventDate: 2011-07-07; habitat: alpine meadow
- **Type status:** Other material. **Occurrence:** recordedBy: Kuntner, Gregorič, Čandek; sex: 1 female; **Location:** locationID: CH15; country: Switzerland; locality: Glarus Alps, near Affeier; minimumElevationInMeters: 817; maximumElevationInMeters: 817; decimalLatitude: 46.7606; decimalLongitude: 9.0933; **Event:** eventDate: 2011-07-10; habitat: meadow and forest

#### Pocadicnemis pumila

(Blackwall, 1841)

##### Materials

- **Type status:** Other material. **Occurrence:** recordedBy: Kuntner, Gregorič, Čandek; sex: 8 females, 1 male; **Location:** locationID: CH02; country: Switzerland; locality: Bernese Alps, Gasteretal; minimumElevationInMeters: 1698; maximumElevationInMeters: 1698; decimalLatitude: 46.4486; decimalLongitude: 7.7438; **Event:** eventDate: 2011-07-07; habitat: spruce thicket and grass
- **Type status:** Other material. **Occurrence:** recordedBy: Kuntner, Gregorič, Čandek; sex: 3 females; **Location:** locationID: CH05; country: Switzerland; locality: Bernese Alps, Gasteretal; minimumElevationInMeters: 1380; maximumElevationInMeters: 1380; decimalLatitude: 46.4674; decimalLongitude: 7.6640; **Event:** eventDate: 2011-07-07; habitat: river vegetation
- **Type status:** Other material. **Occurrence:** recordedBy: Kuntner, Gregorič, Čandek; sex: 4 females; **Location:** locationID: CH11; country: Switzerland; locality: Bernese Alps, Lake Brienz; minimumElevationInMeters: 600; maximumElevationInMeters: 600; decimalLatitude: 46.7569; decimalLongitude: 8.0107; **Event:** eventDate: 2011-07-10; habitat: meadows and forest
- **Type status:** Other material. **Occurrence:** recordedBy: Kuntner, Gregorič, Čandek; sex: 1 female; **Location:** locationID: CH12; country: Switzerland; locality: Bernese Alps, Nessental; minimumElevationInMeters: 930; maximumElevationInMeters: 930; decimalLatitude: 46.7213; decimalLongitude: 8.3039; **Event:** eventDate: 2011-07-10; habitat: grassland and loan trees
- **Type status:** Other material. **Occurrence:** recordedBy: Kuntner, Gregorič, Čandek; sex: 2 females; **Location:** locationID: CH25; country: Switzerland; locality: Grison Alps, Alp Flix, Salategnas; minimumElevationInMeters: 1950; maximumElevationInMeters: 1950; decimalLatitude: 46.5159; decimalLongitude: 9.6496; **Event:** eventDate: 2011-07-12/16; habitat: meadow and shrubs at stream
- **Type status:** Other material. **Occurrence:** recordedBy: Kuntner, Gregorič, Čandek; sex: 1 female; **Location:** locationID: CH27; country: Switzerland; locality: Grison Alps, road to Davos; minimumElevationInMeters: 1180; maximumElevationInMeters: 1180; decimalLatitude: 46.6808; decimalLongitude: 9.6557; **Event:** eventDate: 2011-07-15; habitat: roadside vegetation and forest edge
- **Type status:** Other material. **Occurrence:** recordedBy: Čandek; sex: 1 male; **Location:** locationID: SI61; country: Slovenia; locality: Sekirišče; minimumElevationInMeters: 750; maximumElevationInMeters: 750; decimalLatitude: 45.8631; decimalLongitude: 14.5367; **Event:** eventDate: 2011-06-23/2012-06-21; habitat: house, grassland, overgrowth

#### Porrhomma pallidum

Jackson, 1913

##### Materials

- **Type status:** Other material. **Occurrence:** recordedBy: Kuntner, Gregorič, Čandek; sex: 1 female; **Location:** locationID: CH12; country: Switzerland; locality: Bernese Alps, Nessental; minimumElevationInMeters: 930; maximumElevationInMeters: 930; decimalLatitude: 46.7213; decimalLongitude: 8.3039; **Event:** eventDate: 2011-07-10; habitat: grassland and loan trees

#### Porrhomma pygmaeum

(Blackwall, 1834)

##### Materials

- **Type status:** Other material. **Occurrence:** recordedBy: Kostanjšek, RTŠB 2012; sex: 1 male; **Location:** locationID: SI17; country: Slovenia; locality: Novokračine; minimumElevationInMeters: 502; maximumElevationInMeters: 502; decimalLatitude: 45.4910; decimalLongitude: 14.3015; **Event:** eventDate: 2012-07-22; habitat: Novokrajska cave

#### Scotinotylus alpigena

(L. Koch, 1869)

##### Materials

- **Type status:** Other material. **Occurrence:** recordedBy: Kuntner, Gregorič, Čandek; sex: 4 females; **Location:** locationID: CH26; country: Switzerland; locality: Grison Alps, Alp Flix, Salategnas; minimumElevationInMeters: 1987; maximumElevationInMeters: 1987; decimalLatitude: 46.5166; decimalLongitude: 9.6516; **Event:** eventDate: 2011-07-14; habitat: grassland
- **Type status:** Other material. **Occurrence:** recordedBy: Kuntner, Gregorič, Čandek; sex: 2 females; **Location:** locationID: CH32; country: Switzerland; locality: Grison Alps, Alp Flix, Salategnas; minimumElevationInMeters: 1955; maximumElevationInMeters: 1955; decimalLatitude: 46.5203; decimalLongitude: 9.6458; **Event:** eventDate: 2011-07-16; habitat: timberline forest, moss

#### Scotinotylus clavatus

(Schenkel, 1927)

##### Materials

- **Type status:** Other material. **Occurrence:** recordedBy: Kuntner, Gregorič, Čandek; sex: 1 male; **Location:** locationID: CH26; country: Switzerland; locality: Grison Alps, Alp Flix, Salategnas; minimumElevationInMeters: 1987; maximumElevationInMeters: 1987; decimalLatitude: 46.5166; decimalLongitude: 9.6516; **Event:** eventDate: 2011-07-14; habitat: grassland

#### Silometopus elegans

(O. P.-Cambridge, 1872)

##### Materials

- **Type status:** Other material. **Occurrence:** recordedBy: Kuntner, Gregorič, Čandek; sex: 1 female; **Location:** locationID: CH20; country: Switzerland; locality: Grison Alps, Alp Flix, Salategnas; minimumElevationInMeters: 1900; maximumElevationInMeters: 1900; decimalLatitude: 46.5181; decimalLongitude: 9.6480; **Event:** eventDate: 2011-07-12; habitat: grazed meadow

#### Tapinocyba affinis

Lessert, 1907

##### Materials

- **Type status:** Other material. **Occurrence:** recordedBy: Kuntner, Gregorič, Čandek; sex: 3 females, 1 male; **Location:** locationID: CH26; country: Switzerland; locality: Grison Alps, Alp Flix, Salategnas; minimumElevationInMeters: 1987; maximumElevationInMeters: 1987; decimalLatitude: 46.5166; decimalLongitude: 9.6516; **Event:** eventDate: 2011-07-14; habitat: grassland

#### Tenuiphantes alacris

(Blackwall, 1853)

##### Materials

- **Type status:** Other material. **Occurrence:** recordedBy: Kuntner, Gregorič, Čandek; sex: 3 females, 3 males; **Location:** locationID: CH03; country: Switzerland; locality: Bernese Alps, Gasteretal; minimumElevationInMeters: 1520; maximumElevationInMeters: 1520; decimalLatitude: 46.4498; decimalLongitude: 7.7135; **Event:** eventDate: 2011-07-07; habitat: spruce forest
- **Type status:** Other material. **Occurrence:** recordedBy: Kuntner, Gregorič, Čandek; sex: 1 male; **Location:** locationID: CH23; country: Switzerland; locality: Grison Alps, Alp Flix, Salategnas; minimumElevationInMeters: 1900; maximumElevationInMeters: 1900; decimalLatitude: 46.5141; decimalLongitude: 9.6448; **Event:** eventDate: 2011-07-12; habitat: forest opening, grass and shrubs
- **Type status:** Other material. **Occurrence:** recordedBy: Kuntner, Gregorič, Čandek; sex: 1 female; **Location:** locationID: CH31; country: Switzerland; locality: Grison Alps, Alp Flix - Lai Neir; minimumElevationInMeters: 1910; maximumElevationInMeters: 1910; decimalLatitude: 46.5343; decimalLongitude: 9.6375; **Event:** eventDate: 2011-07-16; habitat: lake and swamp around forest

#### Tenuiphantes cristatus

(Menge, 1866)

##### Materials

- **Type status:** Other material. **Occurrence:** recordedBy: Kuntner, Gregorič, Čandek; sex: 3 females; **Location:** locationID: CH03; country: Switzerland; locality: Bernese Alps, Gasteretal; minimumElevationInMeters: 1520; maximumElevationInMeters: 1520; decimalLatitude: 46.4498; decimalLongitude: 7.7135; **Event:** eventDate: 2011-07-07; habitat: spruce forest
- **Type status:** Other material. **Occurrence:** recordedBy: Kuntner, Gregorič, Čandek; sex: 1 female, 1 male; **Location:** locationID: CH16; country: Switzerland; locality: Engadin, Silvaplana; minimumElevationInMeters: 1930; maximumElevationInMeters: 1930; decimalLatitude: 46.4667; decimalLongitude: 9.7946; **Event:** eventDate: 2011-07-11; habitat: Larix and Pinus forest

#### Tenuiphantes flavipes

(Blackwall, 1854)

##### Materials

- **Type status:** Other material. **Occurrence:** recordedBy: Kuntner, Gregorič, Čandek; sex: 3 males; **Location:** locationID: CH16; country: Switzerland; locality: Engadin, Silvaplana; minimumElevationInMeters: 1930; maximumElevationInMeters: 1930; decimalLatitude: 46.4667; decimalLongitude: 9.7946; **Event:** eventDate: 2011-07-11; habitat: Larix and Pinus forest
- **Type status:** Other material. **Occurrence:** recordedBy: Kuntner, Gregorič, Čandek; sex: 1 female, 2 males; **Location:** locationID: CH27; country: Switzerland; locality: Grison Alps, road to Davos; minimumElevationInMeters: 1180; maximumElevationInMeters: 1180; decimalLatitude: 46.6808; decimalLongitude: 9.6557; **Event:** eventDate: 2011-07-15; habitat: roadside vegetation and forest edge
- **Type status:** Other material. **Occurrence:** recordedBy: Kostanjšek, RTŠB 2011; sex: 1 female; **Location:** locationID: SI01; country: Slovenia; locality: Biš; minimumElevationInMeters: 225; maximumElevationInMeters: 225; decimalLatitude: 46.5374; decimalLongitude: 15.8963; **Event:** eventDate: 2011-07-22; habitat: forest
- **Type status:** Other material. **Occurrence:** recordedBy: Kuntner, Gregorič, Lokovšek; sex: 1 male; **Location:** locationID: SI39; country: Slovenia; locality: Primostek; minimumElevationInMeters: 157; maximumElevationInMeters: 157; decimalLatitude: 45.6299; decimalLongitude: 15.2997; **Event:** eventDate: 2010-08-24; habitat: grassland

#### Tenuiphantes jacksoni

(Schenkel, 1925)

##### Materials

- **Type status:** Other material. **Occurrence:** recordedBy: Kuntner, Gregorič, Čandek; sex: 3 females, 1 male; **Location:** locationID: CH21; country: Switzerland; locality: Grison Alps, Alp Flix, Salategnas; minimumElevationInMeters: 1970; maximumElevationInMeters: 1970; decimalLatitude: 46.5194; decimalLongitude: 9.6490; **Event:** eventDate: 2011-07-12; habitat: swamp grazed vegetation

#### Tenuiphantes jacksonoides

(van Helsdingen, 1977)

##### Materials

- **Type status:** Other material. **Occurrence:** recordedBy: Kuntner, Gregorič, Čandek; sex: 1 male; **Location:** locationID: CH12; country: Switzerland; locality: Bernese Alps, Nessental; minimumElevationInMeters: 930; maximumElevationInMeters: 930; decimalLatitude: 46.7213; decimalLongitude: 8.3039; **Event:** eventDate: 2011-07-10; habitat: grassland and loan trees
- **Type status:** Other material. **Occurrence:** recordedBy: Kuntner, Gregorič, Čandek; sex: 1 female, 4 males; **Location:** locationID: CH22; country: Switzerland; locality: Grison Alps, Alp Flix, Salategnas; minimumElevationInMeters: 1900; maximumElevationInMeters: 1900; decimalLatitude: 46.5152; decimalLongitude: 9.6466; **Event:** eventDate: 2011-07-12; habitat: forest ground
- **Type status:** Other material. **Occurrence:** recordedBy: Kuntner, Gregorič, Čandek; sex: 4 females, 3 males; **Location:** locationID: CH23; country: Switzerland; locality: Grison Alps, Alp Flix, Salategnas; minimumElevationInMeters: 1900; maximumElevationInMeters: 1900; decimalLatitude: 46.5141; decimalLongitude: 9.6448; **Event:** eventDate: 2011-07-12; habitat: forest opening, grass and shrubs
- **Type status:** Other material. **Occurrence:** recordedBy: Kuntner, Gregorič, Čandek; sex: 7 females; **Location:** locationID: CH28; country: Switzerland; locality: Grison Alps, Alp Flix, Salategnas; minimumElevationInMeters: 1713; maximumElevationInMeters: 1713; decimalLatitude: 46.5165; decimalLongitude: 9.6387; **Event:** eventDate: 2011-07-15; habitat: forest edge

#### Tenuiphantes mengei

(Kulczyn'ski, 1887)

##### Materials

- **Type status:** Other material. **Occurrence:** recordedBy: Kuntner, Gregorič, Čandek; sex: 4 females; **Location:** locationID: CH01; country: Switzerland; locality: Bernese Alps, Gasteretal; minimumElevationInMeters: 1662; maximumElevationInMeters: 1662; decimalLatitude: 46.4457; decimalLongitude: 7.7413; **Event:** eventDate: 2011-07-07; habitat: alpine meadow
- **Type status:** Other material. **Occurrence:** recordedBy: Kuntner, Gregorič, Čandek; sex: 14 females, 7 males; **Location:** locationID: CH02; country: Switzerland; locality: Bernese Alps, Gasteretal; minimumElevationInMeters: 1698; maximumElevationInMeters: 1698; decimalLatitude: 46.4486; decimalLongitude: 7.7438; **Event:** eventDate: 2011-07-07; habitat: spruce thicket and grass
- **Type status:** Other material. **Occurrence:** recordedBy: Kuntner, Gregorič, Čandek; sex: 1 female; **Location:** locationID: CH05; country: Switzerland; locality: Bernese Alps, Gasteretal; minimumElevationInMeters: 1380; maximumElevationInMeters: 1380; decimalLatitude: 46.4674; decimalLongitude: 7.6640; **Event:** eventDate: 2011-07-07; habitat: river vegetation
- **Type status:** Other material. **Occurrence:** recordedBy: Kuntner, Gregorič, Čandek; sex: 3 females, 2 males; **Location:** locationID: CH06; country: Switzerland; locality: Bernese Alps, Kandersteg; minimumElevationInMeters: 1677; maximumElevationInMeters: 1677; decimalLatitude: 46.5020; decimalLongitude: 7.6992; **Event:** eventDate: 2011-07-07; habitat: alpine meadow
- **Type status:** Other material. **Occurrence:** recordedBy: Kuntner, Gregorič, Čandek; sex: 5 females, 1 male; **Location:** locationID: CH09; country: Switzerland; locality: Pennine Alps, Mattertal; minimumElevationInMeters: 1447; maximumElevationInMeters: 1447; decimalLatitude: 46.0976; decimalLongitude: 7.7789; **Event:** eventDate: 2011-07-08; habitat: forest and meadow near river
- **Type status:** Other material. **Occurrence:** recordedBy: Kuntner, Gregorič, Čandek; sex: 1 female, 1 male; **Location:** locationID: CH12; country: Switzerland; locality: Bernese Alps, Nessental; minimumElevationInMeters: 930; maximumElevationInMeters: 930; decimalLatitude: 46.7213; decimalLongitude: 8.3039; **Event:** eventDate: 2011-07-10; habitat: grassland and loan trees
- **Type status:** Other material. **Occurrence:** recordedBy: Kuntner, Gregorič, Čandek; sex: 4 females; **Location:** locationID: CH13; country: Switzerland; locality: Bernese Alps, Sustenpass; minimumElevationInMeters: 2040; maximumElevationInMeters: 2040; decimalLatitude: 46.7330; decimalLongitude: 8.4324; **Event:** eventDate: 2011-07-10; habitat: alpine grassland and shrubs
- **Type status:** Other material. **Occurrence:** recordedBy: Kuntner, Gregorič, Čandek; sex: 1 female; **Location:** locationID: CH16; country: Switzerland; locality: Engadin, Silvaplana; minimumElevationInMeters: 1930; maximumElevationInMeters: 1930; decimalLatitude: 46.4667; decimalLongitude: 9.7946; **Event:** eventDate: 2011-07-11; habitat: Larix and Pinus forest
- **Type status:** Other material. **Occurrence:** recordedBy: Kuntner, Gregorič, Čandek; sex: 1 male; **Location:** locationID: CH17; country: Switzerland; locality: Engadin, Bivio; minimumElevationInMeters: 1780; maximumElevationInMeters: 1780; decimalLatitude: 46.4753; decimalLongitude: 9.6469; **Event:** eventDate: 2011-07-11; habitat: forest and river edge
- **Type status:** Other material. **Occurrence:** recordedBy: Kuntner, Gregorič, Čandek; sex: 2 females; **Location:** locationID: CH23; country: Switzerland; locality: Grison Alps, Alp Flix, Salategnas; minimumElevationInMeters: 1900; maximumElevationInMeters: 1900; decimalLatitude: 46.5141; decimalLongitude: 9.6448; **Event:** eventDate: 2011-07-12; habitat: forest opening, grass and shrubs
- **Type status:** Other material. **Occurrence:** recordedBy: Kuntner, Gregorič, Čandek; sex: 1 female, 3 males; **Location:** locationID: CH24; country: Switzerland; locality: Grison Alps, Alp Flix, Salategnas; minimumElevationInMeters: 1830; maximumElevationInMeters: 1830; decimalLatitude: 46.5131; decimalLongitude: 9.6430; **Event:** eventDate: 2011-07-12; habitat: meadow and forest
- **Type status:** Other material. **Occurrence:** recordedBy: Kuntner, Gregorič, Čandek; sex: 1 female, 1 male; **Location:** locationID: CH25; country: Switzerland; locality: Grison Alps, Alp Flix, Salategnas; minimumElevationInMeters: 1950; maximumElevationInMeters: 1950; decimalLatitude: 46.5159; decimalLongitude: 9.6496; **Event:** eventDate: 2011-07-12/16; habitat: meadow and shrubs at stream
- **Type status:** Other material. **Occurrence:** recordedBy: Kuntner, Gregorič, Čandek; sex: 3 females, 3 males; **Location:** locationID: CH28; country: Switzerland; locality: Grison Alps, Alp Flix, Salategnas; minimumElevationInMeters: 1713; maximumElevationInMeters: 1713; decimalLatitude: 46.5165; decimalLongitude: 9.6387; **Event:** eventDate: 2011-07-15; habitat: forest edge
- **Type status:** Other material. **Occurrence:** recordedBy: Kuntner, Gregorič, Čandek; sex: 4 females, 2 males; **Location:** locationID: CH30; country: Switzerland; locality: Grison Alps, Alp Flix - Lai Flix; minimumElevationInMeters: 1967; maximumElevationInMeters: 1967; decimalLatitude: 46.5358; decimalLongitude: 9.6409; **Event:** eventDate: 2011-07-16; habitat: next to alpine lake
- **Type status:** Other material. **Occurrence:** recordedBy: Kuntner, Gregorič, Čandek; sex: 2 females, 1 male; **Location:** locationID: CH31; country: Switzerland; locality: Grison Alps, Alp Flix - Lai Neir; minimumElevationInMeters: 1910; maximumElevationInMeters: 1910; decimalLatitude: 46.5343; decimalLongitude: 9.6375; **Event:** eventDate: 2011-07-16; habitat: lake and swamp around forest

#### Tenuiphantes tenebricola

(Wider, 1834)

##### Materials

- **Type status:** Other material. **Occurrence:** recordedBy: Kuntner, Gregorič, Čandek; sex: 16 females, 6 males; **Location:** locationID: CH03; country: Switzerland; locality: Bernese Alps, Gasteretal; minimumElevationInMeters: 1520; maximumElevationInMeters: 1520; decimalLatitude: 46.4498; decimalLongitude: 7.7135; **Event:** eventDate: 2011-07-07; habitat: spruce forest
- **Type status:** Other material. **Occurrence:** recordedBy: Kuntner, Gregorič, Čandek; sex: 1 female, 2 males; **Location:** locationID: CH16; country: Switzerland; locality: Engadin, Silvaplana; minimumElevationInMeters: 1930; maximumElevationInMeters: 1930; decimalLatitude: 46.4667; decimalLongitude: 9.7946; **Event:** eventDate: 2011-07-11; habitat: Larix and Pinus forest
- **Type status:** Other material. **Occurrence:** recordedBy: Kuntner, Gregorič, Čandek; sex: 1 female; **Location:** locationID: CH17; country: Switzerland; locality: Engadin, Bivio; minimumElevationInMeters: 1780; maximumElevationInMeters: 1780; decimalLatitude: 46.4753; decimalLongitude: 9.6469; **Event:** eventDate: 2011-07-11; habitat: forest and river edge
- **Type status:** Other material. **Occurrence:** recordedBy: Kuntner, Gregorič, Čandek; sex: 8 females, 1 male; **Location:** locationID: CH24; country: Switzerland; locality: Grison Alps, Alp Flix, Salategnas; minimumElevationInMeters: 1830; maximumElevationInMeters: 1830; decimalLatitude: 46.5131; decimalLongitude: 9.6430; **Event:** eventDate: 2011-07-12; habitat: meadow and forest
- **Type status:** Other material. **Occurrence:** recordedBy: Kuntner, Gregorič, Čandek; sex: 1 female; **Location:** locationID: CH28; country: Switzerland; locality: Grison Alps, Alp Flix, Salategnas; minimumElevationInMeters: 1713; maximumElevationInMeters: 1713; decimalLatitude: 46.5165; decimalLongitude: 9.6387; **Event:** eventDate: 2011-07-15; habitat: forest edge
- **Type status:** Other material. **Occurrence:** recordedBy: Kuntner, Gregorič, Čandek; sex: 3 females, 6 males; **Location:** locationID: CH31; country: Switzerland; locality: Grison Alps, Alp Flix - Lai Neir; minimumElevationInMeters: 1910; maximumElevationInMeters: 1910; decimalLatitude: 46.5343; decimalLongitude: 9.6375; **Event:** eventDate: 2011-07-16; habitat: lake and swamp around forest

#### Tenuiphantes tenuis

(Blackwall, 1852)

##### Materials

- **Type status:** Other material. **Occurrence:** recordedBy: Gregorič, Kuntner, Čandek; sex: 1 male; **Location:** locationID: SI48; country: Slovenia; locality: Ljubljana, center; minimumElevationInMeters: 291; maximumElevationInMeters: 291; decimalLatitude: 46.0434; decimalLongitude: 14.5041; **Event:** eventDate: 2011-05-24/2012-06-19; habitat: house
- **Type status:** Other material. **Occurrence:** recordedBy: Čandek; sex: 1 female; **Location:** locationID: SI61; country: Slovenia; locality: Sekirišče; minimumElevationInMeters: 750; maximumElevationInMeters: 750; decimalLatitude: 45.8631; decimalLongitude: 14.5367; **Event:** eventDate: 2011-06-23/2012-06-21; habitat: house, grassland, overgrowth

#### Theonina cornix

(Simon, 1881)

##### Materials

- **Type status:** Other material. **Occurrence:** recordedBy: Kostanjšek, RTŠB 2012; sex: 1 male; **Location:** locationID: SI23; country: Slovenia; locality: Šembije; minimumElevationInMeters: 615; maximumElevationInMeters: 615; decimalLatitude: 45.6067; decimalLongitude: 14.2410; **Event:** eventDate: 2012-07-26; habitat: forest edge

#### Tiso aestivus

(L. Koch, 1872)

##### Materials

- **Type status:** Other material. **Occurrence:** recordedBy: Kuntner, Gregorič, Čandek; sex: 2 males; **Location:** locationID: CH07; country: Switzerland; locality: Bernese Alps, Rothorn; minimumElevationInMeters: 3100; maximumElevationInMeters: 3100; decimalLatitude: 46.0207; decimalLongitude: 7.7989; **Event:** eventDate: 2011-07-08; habitat: alpine rocks and grass tussocks

#### Tiso vagans

(Blackwall, 1834)

##### Materials

- **Type status:** Other material. **Occurrence:** recordedBy: Kuntner, Gregorič, Čandek; sex: 4 males; **Location:** locationID: CH06; country: Switzerland; locality: Bernese Alps, Kandersteg; minimumElevationInMeters: 1677; maximumElevationInMeters: 1677; decimalLatitude: 46.5020; decimalLongitude: 7.6992; **Event:** eventDate: 2011-07-07; habitat: alpine meadow
- **Type status:** Other material. **Occurrence:** recordedBy: Kuntner, Gregorič, Čandek; sex: 2 males; **Location:** locationID: CH09; country: Switzerland; locality: Pennine Alps, Mattertal; minimumElevationInMeters: 1447; maximumElevationInMeters: 1447; decimalLatitude: 46.0976; decimalLongitude: 7.7789; **Event:** eventDate: 2011-07-08; habitat: forest and meadow near river
- **Type status:** Other material. **Occurrence:** recordedBy: Kuntner, Gregorič, Čandek; sex: 1 male; **Location:** locationID: CH11; country: Switzerland; locality: Bernese Alps, Lake Brienz; minimumElevationInMeters: 600; maximumElevationInMeters: 600; decimalLatitude: 46.7569; decimalLongitude: 8.0107; **Event:** eventDate: 2011-07-10; habitat: meadows and forest
- **Type status:** Other material. **Occurrence:** recordedBy: Kuntner, Gregorič, Čandek; sex: 2 males; **Location:** locationID: CH13; country: Switzerland; locality: Bernese Alps, Sustenpass; minimumElevationInMeters: 2040; maximumElevationInMeters: 2040; decimalLatitude: 46.7330; decimalLongitude: 8.4324; **Event:** eventDate: 2011-07-10; habitat: alpine grassland and shrubs
- **Type status:** Other material. **Occurrence:** recordedBy: Kuntner, Gregorič, Čandek; sex: 1 female; **Location:** locationID: CH22; country: Switzerland; locality: Grison Alps, Alp Flix, Salategnas; minimumElevationInMeters: 1900; maximumElevationInMeters: 1900; decimalLatitude: 46.5152; decimalLongitude: 9.6466; **Event:** eventDate: 2011-07-12; habitat: forest ground
- **Type status:** Other material. **Occurrence:** recordedBy: Kuntner, Gregorič, Čandek; sex: 1 male; **Location:** locationID: CH23; country: Switzerland; locality: Grison Alps, Alp Flix, Salategnas; minimumElevationInMeters: 1900; maximumElevationInMeters: 1900; decimalLatitude: 46.5141; decimalLongitude: 9.6448; **Event:** eventDate: 2011-07-12; habitat: forest opening, grass and shrubs
- **Type status:** Other material. **Occurrence:** recordedBy: Kuntner, Gregorič, Čandek; sex: 1 male; **Location:** locationID: CH24; country: Switzerland; locality: Grison Alps, Alp Flix, Salategnas; minimumElevationInMeters: 1830; maximumElevationInMeters: 1830; decimalLatitude: 46.5131; decimalLongitude: 9.6430; **Event:** eventDate: 2011-07-12; habitat: meadow and forest

#### Troglohyphantes sp.

##### Materials

- **Type status:** Other material. **Occurrence:** recordedBy: Kuntner, Gregorič, Čandek; sex: 3 females; **Location:** locationID: CH18; country: Switzerland; locality: Grison Alps, Alp Flix, Salategnas; minimumElevationInMeters: 1900; maximumElevationInMeters: 1900; decimalLatitude: 46.5166; decimalLongitude: 9.6523; **Event:** eventDate: 2011-07-12/19; habitat: around house

#### Walckenaeria antica

(Wider, 1834)

##### Materials

- **Type status:** Other material. **Occurrence:** recordedBy: Kuntner, Gregorič, Čandek; sex: 1 female; **Location:** locationID: CH30; country: Switzerland; locality: Grison Alps, Alp Flix - Lai Flix; minimumElevationInMeters: 1967; maximumElevationInMeters: 1967; decimalLatitude: 46.5358; decimalLongitude: 9.6409; **Event:** eventDate: 2011-07-16; habitat: next to alpine lake

#### Walckenaeria furcillata

(Menge, 1869)

##### Materials

- **Type status:** Other material. **Occurrence:** recordedBy: Kuntner, Gregorič, Čandek; sex: 1 female; **Location:** locationID: CH11; country: Switzerland; locality: Bernese Alps, Lake Brienz; minimumElevationInMeters: 600; maximumElevationInMeters: 600; decimalLatitude: 46.7569; decimalLongitude: 8.0107; **Event:** eventDate: 2011-07-10; habitat: meadows and forest

#### Liocranidae

Simon, 1897

#### Agroeca brunnea

(Blackwall, 1833)

##### Materials

- **Type status:** Other material. **Occurrence:** recordedBy: Čandek; sex: 1 female, 1 male; **Location:** locationID: SI61; country: Slovenia; locality: Sekirišče; minimumElevationInMeters: 750; maximumElevationInMeters: 750; decimalLatitude: 45.8631; decimalLongitude: 14.5367; **Event:** eventDate: 2011-06-23/2012-06-21; habitat: house, grassland, overgrowth

#### Liocranum rupicola

(Walckenaer, 1830)

##### Materials

- **Type status:** Other material. **Occurrence:** recordedBy: Kuntner, Čandek; sex: 1 female; **Location:** locationID: SI50; country: Slovenia; locality: Sp. Prapreče; minimumElevationInMeters: 351; maximumElevationInMeters: 351; decimalLatitude: 46.1620; decimalLongitude: 14.6933; **Event:** eventDate: 2010-08-03/2012-05-28; habitat: house and surroundings

#### Lycosidae

Sundevall, 1833

#### Alopecosa accentuata

(Latreille, 1817)

##### Materials

- **Type status:** Other material. **Occurrence:** recordedBy: Kuntner, Gregorič, Čandek; sex: 1 female; **Location:** locationID: CH25; country: Switzerland; locality: Grison Alps, Alp Flix, Salategnas; minimumElevationInMeters: 1950; maximumElevationInMeters: 1950; decimalLatitude: 46.5159; decimalLongitude: 9.6496; **Event:** eventDate: 2011-07-12/16; habitat: meadow and shrubs at stream

#### Alopecosa aculeata

(Clerck, 1757)

##### Materials

- **Type status:** Other material. **Occurrence:** recordedBy: Kuntner, Gregorič, Čandek; sex: 1 female; **Location:** locationID: CH23; country: Switzerland; locality: Grison Alps, Alp Flix, Salategnas; minimumElevationInMeters: 1900; maximumElevationInMeters: 1900; decimalLatitude: 46.5141; decimalLongitude: 9.6448; **Event:** eventDate: 2011-07-12; habitat: forest opening, grass and shrubs

#### Alopecosa pulverulenta

(Clerck, 1757)

##### Materials

- **Type status:** Other material. **Occurrence:** recordedBy: Kuntner, Gregorič, Čandek; sex: 1 female; **Location:** locationID: CH30; country: Switzerland; locality: Grison Alps, Alp Flix - Lai Flix; minimumElevationInMeters: 1967; maximumElevationInMeters: 1967; decimalLatitude: 46.5358; decimalLongitude: 9.6409; **Event:** eventDate: 2011-07-16; habitat: next to alpine lake
- **Type status:** Other material. **Occurrence:** recordedBy: Kuntner, Gregorič, Čandek; sex: 1 male; **Location:** locationID: CH31; country: Switzerland; locality: Grison Alps, Alp Flix - Lai Neir; minimumElevationInMeters: 1910; maximumElevationInMeters: 1910; decimalLatitude: 46.5343; decimalLongitude: 9.6375; **Event:** eventDate: 2011-07-16; habitat: lake and swamp around forest
- **Type status:** Other material. **Occurrence:** recordedBy: Kuntner, Gregorič, Čandek; sex: 1 male; **Location:** locationID: CH32; country: Switzerland; locality: Grison Alps, Alp Flix, Salategnas; minimumElevationInMeters: 1955; maximumElevationInMeters: 1955; decimalLatitude: 46.5203; decimalLongitude: 9.6458; **Event:** eventDate: 2011-07-16; habitat: timberline forest, moss
- **Type status:** Other material. **Occurrence:** recordedBy: Čandek; sex: 1 female; **Location:** locationID: SI38; country: Slovenia; locality: Poreče; minimumElevationInMeters: 135; maximumElevationInMeters: 135; decimalLatitude: 45.8188; decimalLongitude: 13.9692; **Event:** eventDate: 2011-05-08; habitat: grassland
- **Type status:** Other material. **Occurrence:** recordedBy: Čandek; sex: 1 male; **Location:** locationID: SI43; country: Slovenia; locality: Vipava; minimumElevationInMeters: 114; maximumElevationInMeters: 114; decimalLatitude: 45.8282; decimalLongitude: 13.9594; **Event:** eventDate: 2011-05-08; habitat: grassland
- **Type status:** Other material. **Occurrence:** recordedBy: Gregorič, Čandek, Kralj-Fišer; sex: 1 female, 4 males; **Location:** locationID: SI52; country: Slovenia; locality: Dinaric Karst, Griže; minimumElevationInMeters: 484; maximumElevationInMeters: 484; decimalLatitude: 45.7506; decimalLongitude: 13.9509; **Event:** eventDate: 2011-04-04/05-10; habitat: overgrowth
- **Type status:** Other material. **Occurrence:** recordedBy: Čandek; sex: 2 females, 3 males; **Location:** locationID: SI60; country: Slovenia; locality: Budanje; minimumElevationInMeters: 295; maximumElevationInMeters: 295; decimalLatitude: 45.8799; decimalLongitude: 13.9459; **Event:** eventDate: 2011-05-07; habitat: forest clearing

#### Alopecosa sulzeri

(Pavesi, 1873)

##### Materials

- **Type status:** Other material. **Occurrence:** recordedBy: Čandek; sex: 1 female; **Location:** locationID: SI58; country: Slovenia; locality: Budanje; minimumElevationInMeters: 243; maximumElevationInMeters: 243; decimalLatitude: 45.8743; decimalLongitude: 13.9497; **Event:** eventDate: 2011-05-07; habitat: school and surroundings

#### Alopecosa trabalis

(Clerck, 1757)

##### Materials

- **Type status:** Other material. **Occurrence:** recordedBy: Gregorič, Čandek; sex: 1 female; **Location:** locationID: SI53; country: Slovenia; locality: Dinaric Karst, Griže; minimumElevationInMeters: 434; maximumElevationInMeters: 434; decimalLatitude: 45.7548; decimalLongitude: 13.9495; **Event:** eventDate: 2011-05-10/2011-06-21; habitat: grassland
- **Type status:** Other material. **Occurrence:** recordedBy: Čandek; sex: 2 females; **Location:** locationID: SI61; country: Slovenia; locality: Sekirišče; minimumElevationInMeters: 750; maximumElevationInMeters: 750; decimalLatitude: 45.8631; decimalLongitude: 14.5367; **Event:** eventDate: 2011-06-23/2012-06-21; habitat: house, grassland, overgrowth

#### Arctosa fulvolineata

(Lucas, 1846)

##### Materials

- **Type status:** Other material. **Occurrence:** recordedBy: Čandek; sex: 1 male; **Location:** locationID: SI30; country: Slovenia; locality: Ig, Iški Vintgar; minimumElevationInMeters: 371; maximumElevationInMeters: 371; decimalLatitude: 45.9090; decimalLongitude: 14.4955; **Event:** eventDate: 2011-07-31; habitat: forest

#### Arctosa lutetiana

(Simon, 1876)

##### Materials

- **Type status:** Other material. **Occurrence:** recordedBy: Kostanjšek, RTŠB 2011; sex: 1 female; **Location:** locationID: SI01; country: Slovenia; locality: Biš; minimumElevationInMeters: 225; maximumElevationInMeters: 225; decimalLatitude: 46.5374; decimalLongitude: 15.8963; **Event:** eventDate: 2011-07-22; habitat: forest

#### Arctosa maculata

(Hahn, 1822)

##### Materials

- **Type status:** Other material. **Occurrence:** recordedBy: Kostanjšek, RTŠB 2012; sex: 1 female; **Location:** locationID: SI27; country: Slovenia; locality: Buje; minimumElevationInMeters: 370; maximumElevationInMeters: 370; decimalLatitude: 45.6400; decimalLongitude: 14.0875; **Event:** eventDate: 2012-07-20; habitat: dry river basin

#### Aulonia albimana

(Walckenaer, 1805)

##### Materials

- **Type status:** Other material. **Occurrence:** recordedBy: Čandek; sex: 1 female, 6 males; **Location:** locationID: SI61; country: Slovenia; locality: Sekirišče; minimumElevationInMeters: 750; maximumElevationInMeters: 750; decimalLatitude: 45.8631; decimalLongitude: 14.5367; **Event:** eventDate: 2011-06-23/2012-06-21; habitat: house, grassland, overgrowth

#### Hogna radiata

(Latreille, 1817)

##### Materials

- **Type status:** Other material. **Occurrence:** recordedBy: Kuntner, Čandek; sex: 1 male; **Location:** locationID: SI50; country: Slovenia; locality: Sp. Prapreče; minimumElevationInMeters: 351; maximumElevationInMeters: 351; decimalLatitude: 46.1620; decimalLongitude: 14.6933; **Event:** eventDate: 2010-08-03/2012-05-28; habitat: house and surroundings
- **Type status:** Other material. **Occurrence:** recordedBy: Kostanjšek, RTŠB 2011; sex: 1 female; **Location:** locationID: SI68; country: Slovenia; locality: Sv. Jurij ob Ščavnici; minimumElevationInMeters: 235; maximumElevationInMeters: 235; decimalLatitude: 46.5687; decimalLongitude: 16.0223; **Event:** eventDate: 2011-07-22; habitat: school

#### Pardosa agrestis

(Westring, 1861)

##### Materials

- **Type status:** Other material. **Occurrence:** recordedBy: Kostanjšek, RTŠB 2011; sex: 1 female; **Location:** locationID: SI04; country: Slovenia; locality: Cerkvenjak; minimumElevationInMeters: 230; maximumElevationInMeters: 230; decimalLatitude: 46.5641; decimalLongitude: 15.9863; **Event:** eventDate: 2011-07-22; habitat: grassland

#### Pardosa amentata

(Clerck, 1757)

##### Materials

- **Type status:** Other material. **Occurrence:** recordedBy: Kuntner, Gregorič, Čandek; sex: 3 females; **Location:** locationID: CH05; country: Switzerland; locality: Bernese Alps, Gasteretal; minimumElevationInMeters: 1380; maximumElevationInMeters: 1380; decimalLatitude: 46.4674; decimalLongitude: 7.6640; **Event:** eventDate: 2011-07-07; habitat: river vegetation
- **Type status:** Other material. **Occurrence:** recordedBy: Kuntner, Gregorič, Čandek; sex: 8 females, 4 males; **Location:** locationID: CH14; country: Switzerland; locality: Glarus Alps, Oberalppass; minimumElevationInMeters: 2040; maximumElevationInMeters: 2040; decimalLatitude: 46.6617; decimalLongitude: 8.6719; **Event:** eventDate: 2011-07-10; habitat: grassland and shrubs
- **Type status:** Other material. **Occurrence:** recordedBy: Kuntner, Gregorič, Čandek; sex: 2 females, 1 male; **Location:** locationID: CH20; country: Switzerland; locality: Grison Alps, Alp Flix, Salategnas; minimumElevationInMeters: 1900; maximumElevationInMeters: 1900; decimalLatitude: 46.5181; decimalLongitude: 9.6480; **Event:** eventDate: 2011-07-12; habitat: grazed meadow
- **Type status:** Other material. **Occurrence:** recordedBy: Kuntner, Gregorič, Čandek; sex: 1 female; **Location:** locationID: CH21; country: Switzerland; locality: Grison Alps, Alp Flix, Salategnas; minimumElevationInMeters: 1970; maximumElevationInMeters: 1970; decimalLatitude: 46.5194; decimalLongitude: 9.6490; **Event:** eventDate: 2011-07-12; habitat: swamp grazed vegetation
- **Type status:** Other material. **Occurrence:** recordedBy: Kuntner, Gregorič, Čandek; sex: 3 females; **Location:** locationID: CH25; country: Switzerland; locality: Grison Alps, Alp Flix, Salategnas; minimumElevationInMeters: 1950; maximumElevationInMeters: 1950; decimalLatitude: 46.5159; decimalLongitude: 9.6496; **Event:** eventDate: 2011-07-12/16; habitat: meadow and shrubs at stream
- **Type status:** Other material. **Occurrence:** recordedBy: Kuntner, Gregorič, Čandek; sex: 4 females; **Location:** locationID: CH30; country: Switzerland; locality: Grison Alps, Alp Flix - Lai Flix; minimumElevationInMeters: 1967; maximumElevationInMeters: 1967; decimalLatitude: 46.5358; decimalLongitude: 9.6409; **Event:** eventDate: 2011-07-16; habitat: next to alpine lake
- **Type status:** Other material. **Occurrence:** recordedBy: Čandek; sex: 1 female; **Location:** locationID: SI35; country: Slovenia; locality: Menina planina; minimumElevationInMeters: 1373; maximumElevationInMeters: 1373; decimalLatitude: 46.2527; decimalLongitude: 14.8325; **Event:** eventDate: 2011-08-07; habitat: forest clearing

#### Pardosa bifasciata

(C. L. Koch, 1834)

##### Materials

- **Type status:** Other material. **Occurrence:** recordedBy: Kuntner, Gregorič, Čandek; sex: 1 female; **Location:** locationID: CH08; country: Switzerland; locality: Bernese Alps, Rothorn; minimumElevationInMeters: 2250; maximumElevationInMeters: 2250; decimalLatitude: 46.0183; decimalLongitude: 7.7687; **Event:** eventDate: 2011-07-08; habitat: grass, shrubs, spruce
- **Type status:** Other material. **Occurrence:** recordedBy: Gregorič, Čandek; sex: 1 male; **Location:** locationID: SI57; country: Slovenia; locality: Dinaric Karst, Novelo; minimumElevationInMeters: 325; maximumElevationInMeters: 325; decimalLatitude: 45.8482; decimalLongitude: 13.6584; **Event:** eventDate: 2011-05-10; habitat: grassland

#### Pardosa blanda

(C. L. Koch, 1833)

##### Materials

- **Type status:** Other material. **Occurrence:** recordedBy: Kuntner, Gregorič, Čandek; sex: 1 female; **Location:** locationID: CH08; country: Switzerland; locality: Bernese Alps, Rothorn; minimumElevationInMeters: 2250; maximumElevationInMeters: 2250; decimalLatitude: 46.0183; decimalLongitude: 7.7687; **Event:** eventDate: 2011-07-08; habitat: grass, shrubs, spruce
- **Type status:** Other material. **Occurrence:** recordedBy: Kuntner, Gregorič, Čandek; sex: 5 females, 5 males; **Location:** locationID: CH20; country: Switzerland; locality: Grison Alps, Alp Flix, Salategnas; minimumElevationInMeters: 1900; maximumElevationInMeters: 1900; decimalLatitude: 46.5181; decimalLongitude: 9.6480; **Event:** eventDate: 2011-07-12; habitat: grazed meadow
- **Type status:** Other material. **Occurrence:** recordedBy: Kuntner, Gregorič, Čandek; sex: 1 female; **Location:** locationID: CH21; country: Switzerland; locality: Grison Alps, Alp Flix, Salategnas; minimumElevationInMeters: 1970; maximumElevationInMeters: 1970; decimalLatitude: 46.5194; decimalLongitude: 9.6490; **Event:** eventDate: 2011-07-12; habitat: swamp grazed vegetation
- **Type status:** Other material. **Occurrence:** recordedBy: Kuntner, Gregorič, Čandek; sex: 5 females, 1 male; **Location:** locationID: CH25; country: Switzerland; locality: Grison Alps, Alp Flix, Salategnas; minimumElevationInMeters: 1950; maximumElevationInMeters: 1950; decimalLatitude: 46.5159; decimalLongitude: 9.6496; **Event:** eventDate: 2011-07-12/16; habitat: meadow and shrubs at stream

#### Pardosa cf. hyperborea

(Thorell, 1872)

##### Materials

- **Type status:** Other material. **Occurrence:** recordedBy: Kuntner, Gregorič, Čandek; sex: 1 female; **Location:** locationID: CH14; country: Switzerland; locality: Glarus Alps, Oberalppass; minimumElevationInMeters: 2040; maximumElevationInMeters: 2040; decimalLatitude: 46.6617; decimalLongitude: 8.6719; **Event:** eventDate: 2011-07-10; habitat: grassland and shrubs

#### Pardosa cf. lugubris

(Walckenaer, 1802)

##### Materials

- **Type status:** Other material. **Occurrence:** recordedBy: Kuntner, Gregorič, Čandek; sex: 3 females; **Location:** locationID: CH11; country: Switzerland; locality: Bernese Alps, Lake Brienz; minimumElevationInMeters: 600; maximumElevationInMeters: 600; decimalLatitude: 46.7569; decimalLongitude: 8.0107; **Event:** eventDate: 2011-07-10; habitat: meadows and forest
- **Type status:** Other material. **Occurrence:** recordedBy: Kuntner, Gregorič, Čandek; sex: 2 females; **Location:** locationID: CH12; country: Switzerland; locality: Bernese Alps, Nessental; minimumElevationInMeters: 930; maximumElevationInMeters: 930; decimalLatitude: 46.7213; decimalLongitude: 8.3039; **Event:** eventDate: 2011-07-10; habitat: grassland and loan trees
- **Type status:** Other material. **Occurrence:** recordedBy: Kuntner, Gregorič, Čandek; sex: 4 females; **Location:** locationID: CH15; country: Switzerland; locality: Glarus Alps, near Affeier; minimumElevationInMeters: 817; maximumElevationInMeters: 817; decimalLatitude: 46.7606; decimalLongitude: 9.0933; **Event:** eventDate: 2011-07-10; habitat: meadow and forest
- **Type status:** Other material. **Occurrence:** recordedBy: Kuntner, Gregorič, Čandek; sex: 6 females, 1 male; **Location:** locationID: CH27; country: Switzerland; locality: Grison Alps, road to Davos; minimumElevationInMeters: 1180; maximumElevationInMeters: 1180; decimalLatitude: 46.6808; decimalLongitude: 9.6557; **Event:** eventDate: 2011-07-15; habitat: roadside vegetation and forest edge
- **Type status:** Other material. **Occurrence:** recordedBy: Kostanjšek, RTŠB 2011; sex: 1 female; **Location:** locationID: SI15; country: Slovenia; locality: Apače; minimumElevationInMeters: 220; maximumElevationInMeters: 220; decimalLatitude: 46.6804; decimalLongitude: 15.8988; **Event:** eventDate: 2011-07-26; habitat: forest
- **Type status:** Other material. **Occurrence:** recordedBy: Čandek; sex: 1 female; **Location:** locationID: SI38; country: Slovenia; locality: Poreče; minimumElevationInMeters: 135; maximumElevationInMeters: 135; decimalLatitude: 45.8188; decimalLongitude: 13.9692; **Event:** eventDate: 2011-05-08; habitat: grassland
- **Type status:** Other material. **Occurrence:** recordedBy: Kuntner, Lokovšek; sex: 3 females; **Location:** locationID: SI40; country: Slovenia; locality: Slavnik; minimumElevationInMeters: 816; maximumElevationInMeters: 816; decimalLatitude: 45.5499; decimalLongitude: 13.9619; **Event:** eventDate: 2010-08-26; habitat: grassland and forest
- **Type status:** Other material. **Occurrence:** recordedBy: Čandek; sex: 2 females; **Location:** locationID: SI58; country: Slovenia; locality: Budanje; minimumElevationInMeters: 243; maximumElevationInMeters: 243; decimalLatitude: 45.8743; decimalLongitude: 13.9497; **Event:** eventDate: 2011-05-07; habitat: school and surroundings
- **Type status:** Other material. **Occurrence:** recordedBy: Čandek; sex: 3 females, 4 males; **Location:** locationID: SI59; country: Slovenia; locality: Budanje; minimumElevationInMeters: 305; maximumElevationInMeters: 305; decimalLatitude: 45.8797; decimalLongitude: 13.9468; **Event:** eventDate: 2011-05-07; habitat: forest
- **Type status:** Other material. **Occurrence:** recordedBy: Čandek; sex: 2 females; **Location:** locationID: SI60; country: Slovenia; locality: Budanje; minimumElevationInMeters: 295; maximumElevationInMeters: 295; decimalLatitude: 45.8799; decimalLongitude: 13.9459; **Event:** eventDate: 2011-05-07; habitat: forest clearing
- **Type status:** Other material. **Occurrence:** recordedBy: Čandek; sex: 4 females; **Location:** locationID: SI61; country: Slovenia; locality: Sekirišče; minimumElevationInMeters: 750; maximumElevationInMeters: 750; decimalLatitude: 45.8631; decimalLongitude: 14.5367; **Event:** eventDate: 2011-06-23/2012-06-21; habitat: house, grassland, overgrowth

#### Pardosa ferruginea

(L. Koch, 1870)

##### Materials

- **Type status:** Other material. **Occurrence:** recordedBy: Kuntner, Gregorič, Čandek; sex: 1 female; **Location:** locationID: CH23; country: Switzerland; locality: Grison Alps, Alp Flix, Salategnas; minimumElevationInMeters: 1900; maximumElevationInMeters: 1900; decimalLatitude: 46.5141; decimalLongitude: 9.6448; **Event:** eventDate: 2011-07-12; habitat: forest opening, grass and shrubs

#### Pardosa hortensis

(Thorell, 1872)

##### Materials

- **Type status:** Other material. **Occurrence:** recordedBy: Kostanjšek, RTŠB 2012; sex: 1 female; **Location:** locationID: SI13; country: Slovenia; locality: Čepno; minimumElevationInMeters: 555; maximumElevationInMeters: 555; decimalLatitude: 45.6735; decimalLongitude: 14.1068; **Event:** eventDate: 2012-07-22; habitat: grassland

#### Pardosa oreophila

Simon, 1937

##### Materials

- **Type status:** Other material. **Occurrence:** recordedBy: Kuntner, Gregorič, Čandek; sex: 1 female; **Location:** locationID: CH10; country: Switzerland; locality: Bernese Alps, Kleine Scheidegg; minimumElevationInMeters: 2061; maximumElevationInMeters: 2061; decimalLatitude: 46.5853; decimalLongitude: 7.9606; **Event:** eventDate: 2011-07-09; habitat: grassland
- **Type status:** Other material. **Occurrence:** recordedBy: Kuntner, Gregorič, Čandek; sex: 4 males; **Location:** locationID: CH13; country: Switzerland; locality: Bernese Alps, Sustenpass; minimumElevationInMeters: 2040; maximumElevationInMeters: 2040; decimalLatitude: 46.7330; decimalLongitude: 8.4324; **Event:** eventDate: 2011-07-10; habitat: alpine grassland and shrubs

#### Pardosa palustris

(Linnaeus, 1758)

##### Materials

- **Type status:** Other material. **Occurrence:** recordedBy: Kuntner, Gregorič, Čandek; sex: 1 female; **Location:** locationID: CH10; country: Switzerland; locality: Bernese Alps, Kleine Scheidegg; minimumElevationInMeters: 2061; maximumElevationInMeters: 2061; decimalLatitude: 46.5853; decimalLongitude: 7.9606; **Event:** eventDate: 2011-07-09; habitat: grassland
- **Type status:** Other material. **Occurrence:** recordedBy: Kuntner, Gregorič, Čandek; sex: 1 male; **Location:** locationID: CH15; country: Switzerland; locality: Glarus Alps, near Affeier; minimumElevationInMeters: 817; maximumElevationInMeters: 817; decimalLatitude: 46.7606; decimalLongitude: 9.0933; **Event:** eventDate: 2011-07-10; habitat: meadow and forest
- **Type status:** Other material. **Occurrence:** recordedBy: Kuntner, Gregorič, Čandek; sex: 2 females, 1 male; **Location:** locationID: CH19; country: Switzerland; locality: Grison Alps, Alp Flix, Salategnas; minimumElevationInMeters: 1910; maximumElevationInMeters: 1910; decimalLatitude: 46.5172; decimalLongitude: 9.6533; **Event:** eventDate: 2011-07-12; habitat: flat uncut grassland
- **Type status:** Other material. **Occurrence:** recordedBy: Čandek; sex: 1 female; **Location:** locationID: SI61; country: Slovenia; locality: Sekirišče; minimumElevationInMeters: 750; maximumElevationInMeters: 750; decimalLatitude: 45.8631; decimalLongitude: 14.5367; **Event:** eventDate: 2011-06-23/2012-06-21; habitat: house, grassland, overgrowth

#### Pardosa proxima

(C. L. Koch, 1847)

##### Materials

- **Type status:** Other material. **Occurrence:** recordedBy: Kostanjšek, RTŠB 2011; sex: 1 female; **Location:** locationID: SI05; country: Slovenia; locality: Sv. Jurij ob Ščavnici; minimumElevationInMeters: 190; maximumElevationInMeters: 190; decimalLatitude: 46.5509; decimalLongitude: 16.0451; **Event:** eventDate: 2011-07-22; habitat: overgrown river channel

#### Pardosa riparia

(C. L. Koch, 1833)

##### Materials

- **Type status:** Other material. **Occurrence:** recordedBy: Kuntner, Gregorič, Čandek; sex: 6 females, 4 males; **Location:** locationID: CH01; country: Switzerland; locality: Bernese Alps, Gasteretal; minimumElevationInMeters: 1662; maximumElevationInMeters: 1662; decimalLatitude: 46.4457; decimalLongitude: 7.7413; **Event:** eventDate: 2011-07-07; habitat: alpine meadow
- **Type status:** Other material. **Occurrence:** recordedBy: Kuntner, Gregorič, Čandek; sex: 2 females; **Location:** locationID: CH02; country: Switzerland; locality: Bernese Alps, Gasteretal; minimumElevationInMeters: 1698; maximumElevationInMeters: 1698; decimalLatitude: 46.4486; decimalLongitude: 7.7438; **Event:** eventDate: 2011-07-07; habitat: spruce thicket and grass
- **Type status:** Other material. **Occurrence:** recordedBy: Kuntner, Gregorič, Čandek; sex: 6 females; **Location:** locationID: CH05; country: Switzerland; locality: Bernese Alps, Gasteretal; minimumElevationInMeters: 1380; maximumElevationInMeters: 1380; decimalLatitude: 46.4674; decimalLongitude: 7.6640; **Event:** eventDate: 2011-07-07; habitat: river vegetation
- **Type status:** Other material. **Occurrence:** recordedBy: Kuntner, Gregorič, Čandek; sex: 4 females; **Location:** locationID: CH06; country: Switzerland; locality: Bernese Alps, Kandersteg; minimumElevationInMeters: 1677; maximumElevationInMeters: 1677; decimalLatitude: 46.5020; decimalLongitude: 7.6992; **Event:** eventDate: 2011-07-07; habitat: alpine meadow
- **Type status:** Other material. **Occurrence:** recordedBy: Kuntner, Gregorič, Čandek; sex: 3 females, 3 males; **Location:** locationID: CH20; country: Switzerland; locality: Grison Alps, Alp Flix, Salategnas; minimumElevationInMeters: 1900; maximumElevationInMeters: 1900; decimalLatitude: 46.5181; decimalLongitude: 9.6480; **Event:** eventDate: 2011-07-12; habitat: grazed meadow
- **Type status:** Other material. **Occurrence:** recordedBy: Kuntner, Gregorič, Čandek; sex: 15 females, 1 male; **Location:** locationID: CH23; country: Switzerland; locality: Grison Alps, Alp Flix, Salategnas; minimumElevationInMeters: 1900; maximumElevationInMeters: 1900; decimalLatitude: 46.5141; decimalLongitude: 9.6448; **Event:** eventDate: 2011-07-12; habitat: forest opening, grass and shrubs
- **Type status:** Other material. **Occurrence:** recordedBy: Kuntner, Gregorič, Čandek; sex: 9 females, 3 males; **Location:** locationID: CH24; country: Switzerland; locality: Grison Alps, Alp Flix, Salategnas; minimumElevationInMeters: 1830; maximumElevationInMeters: 1830; decimalLatitude: 46.5131; decimalLongitude: 9.6430; **Event:** eventDate: 2011-07-12; habitat: meadow and forest
- **Type status:** Other material. **Occurrence:** recordedBy: Kuntner, Gregorič, Čandek; sex: 2 females; **Location:** locationID: CH25; country: Switzerland; locality: Grison Alps, Alp Flix, Salategnas; minimumElevationInMeters: 1950; maximumElevationInMeters: 1950; decimalLatitude: 46.5159; decimalLongitude: 9.6496; **Event:** eventDate: 2011-07-12/16; habitat: meadow and shrubs at stream
- **Type status:** Other material. **Occurrence:** recordedBy: Kuntner, Gregorič, Čandek; sex: 7 females, 7 males; **Location:** locationID: CH30; country: Switzerland; locality: Grison Alps, Alp Flix - Lai Flix; minimumElevationInMeters: 1967; maximumElevationInMeters: 1967; decimalLatitude: 46.5358; decimalLongitude: 9.6409; **Event:** eventDate: 2011-07-16; habitat: next to alpine lake
- **Type status:** Other material. **Occurrence:** recordedBy: Kuntner, Gregorič, Čandek; sex: 2 females, 2 males; **Location:** locationID: CH31; country: Switzerland; locality: Grison Alps, Alp Flix - Lai Neir; minimumElevationInMeters: 1910; maximumElevationInMeters: 1910; decimalLatitude: 46.5343; decimalLongitude: 9.6375; **Event:** eventDate: 2011-07-16; habitat: lake and swamp around forest
- **Type status:** Other material. **Occurrence:** recordedBy: Gregorič, Čandek; sex: 1 male; **Location:** locationID: SI53; country: Slovenia; locality: Dinaric Karst, Griže; minimumElevationInMeters: 434; maximumElevationInMeters: 434; decimalLatitude: 45.7548; decimalLongitude: 13.9495; **Event:** eventDate: 2011-05-10/2011-06-21; habitat: grassland
- **Type status:** Other material. **Occurrence:** recordedBy: Čandek; sex: 4 females, 1 male; **Location:** locationID: SI61; country: Slovenia; locality: Sekirišče; minimumElevationInMeters: 750; maximumElevationInMeters: 750; decimalLatitude: 45.8631; decimalLongitude: 14.5367; **Event:** eventDate: 2011-06-23/2012-06-21; habitat: house, grassland, overgrowth

#### Pirata piraticus

(Clerck, 1757)

##### Materials

- **Type status:** Other material. **Occurrence:** recordedBy: Kuntner, Gregorič, Čandek; sex: 1 male; **Location:** locationID: CH20; country: Switzerland; locality: Grison Alps, Alp Flix, Salategnas; minimumElevationInMeters: 1900; maximumElevationInMeters: 1900; decimalLatitude: 46.5181; decimalLongitude: 9.6480; **Event:** eventDate: 2011-07-12; habitat: grazed meadow
- **Type status:** Other material. **Occurrence:** recordedBy: Kuntner, Gregorič, Čandek; sex: 1 male; **Location:** locationID: CH31; country: Switzerland; locality: Grison Alps, Alp Flix - Lai Neir; minimumElevationInMeters: 1910; maximumElevationInMeters: 1910; decimalLatitude: 46.5343; decimalLongitude: 9.6375; **Event:** eventDate: 2011-07-16; habitat: lake and swamp around forest

#### Piratula hygrophila

(Thorell, 1872)

##### Materials

- **Type status:** Other material. **Occurrence:** recordedBy: Kostanjšek, RTŠB 2011; sex: 1 female; **Location:** locationID: SI14; country: Slovenia; locality: Spodnji Velovlek; minimumElevationInMeters: 225; maximumElevationInMeters: 225; decimalLatitude: 46.4768; decimalLongitude: 15.9316; **Event:** eventDate: 2011-07-25; habitat: forest

#### Piratula knorri

(Scopoli, 1763)

##### Materials

- **Type status:** Other material. **Occurrence:** recordedBy: Kostanjšek, RTŠB 2011; sex: 1 female; **Location:** locationID: SI14; country: Slovenia; locality: Spodnji Velovlek; minimumElevationInMeters: 225; maximumElevationInMeters: 225; decimalLatitude: 46.4768; decimalLongitude: 15.9316; **Event:** eventDate: 2011-07-25; habitat: forest
- **Type status:** Other material. **Occurrence:** recordedBy: Kostanjšek, RTŠB 2012; sex: 1 female; **Location:** locationID: SI27; country: Slovenia; locality: Buje; minimumElevationInMeters: 370; maximumElevationInMeters: 370; decimalLatitude: 45.6400; decimalLongitude: 14.0875; **Event:** eventDate: 2012-07-20; habitat: dry river basin

#### Trochosa spinipalpis

(F. O. P.-Cambridge, 1895)

##### Materials

- **Type status:** Other material. **Occurrence:** recordedBy: Čandek; sex: 2 females; **Location:** locationID: SI30; country: Slovenia; locality: Ig, Iški Vintgar; minimumElevationInMeters: 371; maximumElevationInMeters: 371; decimalLatitude: 45.9090; decimalLongitude: 14.4955; **Event:** eventDate: 2011-07-31; habitat: forest

#### Xerolycosa nemoralis

(Westring, 1861)

##### Materials

- **Type status:** Other material. **Occurrence:** recordedBy: Kuntner, Gregorič, Čandek; sex: 2 females, 1 male; **Location:** locationID: CH27; country: Switzerland; locality: Grison Alps, road to Davos; minimumElevationInMeters: 1180; maximumElevationInMeters: 1180; decimalLatitude: 46.6808; decimalLongitude: 9.6557; **Event:** eventDate: 2011-07-15; habitat: roadside vegetation and forest edge

#### Mimetidae

Simon, 1881

#### Ero furcata

(Villers, 1789)

##### Materials

- **Type status:** Other material. **Occurrence:** recordedBy: Kuntner, Gregorič, Čandek; sex: 1 male; **Location:** locationID: CH03; country: Switzerland; locality: Bernese Alps, Gasteretal; minimumElevationInMeters: 1520; maximumElevationInMeters: 1520; decimalLatitude: 46.4498; decimalLongitude: 7.7135; **Event:** eventDate: 2011-07-07; habitat: spruce forest

#### Miturgidae

Simon, 1886

#### Cheiracanthium erraticum

(Walckenaer, 1802)

##### Materials

- **Type status:** Other material. **Occurrence:** recordedBy: Kostanjšek, RTŠB 2011; sex: 1 female; **Location:** locationID: SI22; country: Slovenia; locality: Polenšak; minimumElevationInMeters: 235; maximumElevationInMeters: 235; decimalLatitude: 46.4699; decimalLongitude: 16.0227; **Event:** eventDate: 2011-07-29; habitat: forest edge

#### Cheiracanthium mildei

L. Koch, 1864

##### Materials

- **Type status:** Other material. **Occurrence:** recordedBy: Čandek; sex: 1 male; **Location:** locationID: SI46; country: Slovenia; locality: Šešče pri Preboldu; minimumElevationInMeters: 284; maximumElevationInMeters: 285; decimalLatitude: 46.2356; decimalLongitude: 15.1228; **Event:** eventDate: 2011-06-13/2012-06-22; habitat: house and surroundings

#### Cheiracanthium punctorium

(Villers, 1789)

##### Materials

- **Type status:** Other material. **Occurrence:** recordedBy: Kostanjšek, RTŠB 2012; sex: 1 male; **Location:** locationID: SI11; country: Slovenia; locality: Divača; minimumElevationInMeters: 460; maximumElevationInMeters: 460; decimalLatitude: 45.6835; decimalLongitude: 14.0166; **Event:** eventDate: 2012-07-22; habitat: grassland
- **Type status:** Other material. **Occurrence:** recordedBy: Kuntner, Lokovšek; sex: 4 females; **Location:** locationID: SI40; country: Slovenia; locality: Slavnik; minimumElevationInMeters: 816; maximumElevationInMeters: 816; decimalLatitude: 45.5499; decimalLongitude: 13.9619; **Event:** eventDate: 2010-08-26; habitat: grassland and forest

#### Nemesiidae

Simon, 1889

#### Nemesia pannonica

Herman, 1879

##### Materials

- **Type status:** Other material. **Occurrence:** recordedBy: Kostanjšek, RTŠB 2012; sex: 1 female; **Location:** locationID: SI09; country: Slovenia; locality: Divača; minimumElevationInMeters: 445; maximumElevationInMeters: 445; decimalLatitude: 45.6784; decimalLongitude: 13.9952; **Event:** eventDate: 2012-07-22; habitat: grassland

#### Nesticidae

Simon, 1894

#### Nesticus cellulanus

(Clerck, 1757)

##### Materials

- **Type status:** Other material. **Occurrence:** recordedBy: Kostanjšek, RTŠB 2011; sex: 1 female; **Location:** locationID: SI10; country: Slovenia; locality: Sv. Jurij ob Ščavnici; minimumElevationInMeters: 195; maximumElevationInMeters: 195; decimalLatitude: 46.5573; decimalLongitude: 16.0386; **Event:** eventDate: 2011-07-25; habitat: wooden house

#### Oecobiidae

Blackwall, 1862

#### Uroctea durandi

(Latreille, 1809)

##### Materials

- **Type status:** Other material. **Occurrence:** recordedBy: Kuntner, Gregorič, Čandek, Kralj-Fišer, Cheng; sex: 1 female1 juvenile; **Location:** locationID: SI41; country: Slovenia; locality: Socerb, Osp; minimumElevationInMeters: 116; maximumElevationInMeters: 116; decimalLatitude: 45.5819; decimalLongitude: 13.8558; **Event:** eventDate: 2012-06-07; habitat: trail from Socerb to Osp

#### Philodromidae

Thorell, 1870

#### Philodromus albidus

Kulczyn'ski, 1911

##### Materials

- **Type status:** Other material. **Occurrence:** recordedBy: Čandek; sex: 2 females; **Location:** locationID: SI61; country: Slovenia; locality: Sekirišče; minimumElevationInMeters: 750; maximumElevationInMeters: 750; decimalLatitude: 45.8631; decimalLongitude: 14.5367; **Event:** eventDate: 2011-06-23/2012-06-21; habitat: house, grassland, overgrowth

#### Philodromus aureolus

(Clerck, 1757)

##### Materials

- **Type status:** Other material. **Occurrence:** recordedBy: Čandek; sex: 1 female; **Location:** locationID: SI61; country: Slovenia; locality: Sekirišče; minimumElevationInMeters: 750; maximumElevationInMeters: 750; decimalLatitude: 45.8631; decimalLongitude: 14.5367; **Event:** eventDate: 2011-06-23/2012-06-21; habitat: house, grassland, overgrowth

#### Philodromus cespitum

(Walckenaer, 1802)

##### Materials

- **Type status:** Other material. **Occurrence:** recordedBy: Kuntner, Gregorič, Čandek; sex: 1 female; **Location:** locationID: CH31; country: Switzerland; locality: Grison Alps, Alp Flix - Lai Neir; minimumElevationInMeters: 1910; maximumElevationInMeters: 1910; decimalLatitude: 46.5343; decimalLongitude: 9.6375; **Event:** eventDate: 2011-07-16; habitat: lake and swamp around forest
- **Type status:** Other material. **Occurrence:** recordedBy: Kostanjšek, RTŠB 2011; sex: 1 female; **Location:** locationID: SI04; country: Slovenia; locality: Cerkvenjak; minimumElevationInMeters: 230; maximumElevationInMeters: 230; decimalLatitude: 46.5641; decimalLongitude: 15.9863; **Event:** eventDate: 2011-07-22; habitat: grassland

#### Philodromus dispar

Walckenaer, 1826

##### Materials

- **Type status:** Other material. **Occurrence:** recordedBy: Čandek; sex: 1 female; **Location:** locationID: SI59; country: Slovenia; locality: Budanje; minimumElevationInMeters: 305; maximumElevationInMeters: 305; decimalLatitude: 45.8797; decimalLongitude: 13.9468; **Event:** eventDate: 2011-05-07; habitat: forest

#### Philodromus praedatus

O. P.-Cambridge, 1871

##### Materials

- **Type status:** Other material. **Occurrence:** recordedBy: Kuntner, Gregorič, Čandek; sex: 1 male; **Location:** locationID: CH15; country: Switzerland; locality: Glarus Alps, near Affeier; minimumElevationInMeters: 817; maximumElevationInMeters: 817; decimalLatitude: 46.7606; decimalLongitude: 9.0933; **Event:** eventDate: 2011-07-10; habitat: meadow and forest
- **Type status:** Other material. **Occurrence:** recordedBy: Kuntner, Gregorič, Čandek, Kralj-Fišer, Cheng; sex: 1 male; **Location:** locationID: SI41; country: Slovenia; locality: Socerb, Osp; minimumElevationInMeters: 116; maximumElevationInMeters: 116; decimalLatitude: 45.5819; decimalLongitude: 13.8558; **Event:** eventDate: 2012-06-07; habitat: trail from Socerb to Osp

#### Philodromus pulchellus

Lucas, 1846

##### Materials

- **Type status:** Other material. **Occurrence:** recordedBy: Kuntner, Gregorič, Čandek, Kralj-Fišer, Cheng; sex: 5 females; **Location:** locationID: SI41; country: Slovenia; locality: Socerb, Osp; minimumElevationInMeters: 116; maximumElevationInMeters: 116; decimalLatitude: 45.5819; decimalLongitude: 13.8558; **Event:** eventDate: 2012-06-07; habitat: trail from Socerb to Osp

#### Philodromus vagulus

Simon, 1875

##### Materials

- **Type status:** Other material. **Occurrence:** recordedBy: Kuntner, Gregorič, Čandek; sex: 1 female; **Location:** locationID: CH19; country: Switzerland; locality: Grison Alps, Alp Flix, Salategnas; minimumElevationInMeters: 1910; maximumElevationInMeters: 1910; decimalLatitude: 46.5172; decimalLongitude: 9.6533; **Event:** eventDate: 2011-07-12; habitat: flat uncut grassland
- **Type status:** Other material. **Occurrence:** recordedBy: Kuntner, Gregorič, Čandek; sex: 1 female; **Location:** locationID: CH29; country: Switzerland; locality: Grison Alps, Alp Flix - Lai Flix; minimumElevationInMeters: 1960; maximumElevationInMeters: 1960; decimalLatitude: 46.5340; decimalLongitude: 9.6431; **Event:** eventDate: 2011-07-16; habitat: next to alpine lake

#### Thanatus formicinus

(Clerck, 1757)

##### Materials

- **Type status:** Other material. **Occurrence:** recordedBy: Gregorič, Čandek, Kralj-Fišer; sex: 1 female; **Location:** locationID: SI52; country: Slovenia; locality: Dinaric Karst, Griže; minimumElevationInMeters: 484; maximumElevationInMeters: 484; decimalLatitude: 45.7506; decimalLongitude: 13.9509; **Event:** eventDate: 2011-04-04/05-10; habitat: overgrowth

#### Tibellus macellus

Simon, 1875

##### Materials

- **Type status:** Other material. **Occurrence:** recordedBy: Kuntner, Gregorič, Čandek, Kralj-Fišer, Cheng; sex: 3 females, 2 males; **Location:** locationID: SI41; country: Slovenia; locality: Socerb, Osp; minimumElevationInMeters: 116; maximumElevationInMeters: 116; decimalLatitude: 45.5819; decimalLongitude: 13.8558; **Event:** eventDate: 2012-06-07; habitat: trail from Socerb to Osp
- **Type status:** Other material. **Occurrence:** recordedBy: Gregorič, Čandek, Kralj-Fišer; sex: 2 males; **Location:** locationID: SI52; country: Slovenia; locality: Dinaric Karst, Griže; minimumElevationInMeters: 484; maximumElevationInMeters: 484; decimalLatitude: 45.7506; decimalLongitude: 13.9509; **Event:** eventDate: 2011-04-04/05-10; habitat: overgrowth

#### Tibellus oblongus

(Walckenaer, 1802)

##### Materials

- **Type status:** Other material. **Occurrence:** recordedBy: Gregorič, Čandek, Kralj-Fišer; sex: 1 female; **Location:** locationID: SI55; country: Slovenia; locality: Dinaric Karst, Lokvice; minimumElevationInMeters: 273; maximumElevationInMeters: 275; decimalLatitude: 45.8659; decimalLongitude: 13.6102; **Event:** eventDate: 2011-04-04/05-10; habitat: overgrowth

#### Pholcidae

C. L. Koch, 1850

#### Pholcus opilionoides

(Schrank, 1781)

##### Materials

- **Type status:** Other material. **Occurrence:** recordedBy: Kostanjšek, RTŠB 2011; sex: 1 female; **Location:** locationID: SI10; country: Slovenia; locality: Sv. Jurij ob Ščavnici; minimumElevationInMeters: 195; maximumElevationInMeters: 195; decimalLatitude: 46.5573; decimalLongitude: 16.0386; **Event:** eventDate: 2011-07-25; habitat: wooden house
- **Type status:** Other material. **Occurrence:** recordedBy: Kostanjšek, RTŠB 2012; sex: 1 male; **Location:** locationID: SI13; country: Slovenia; locality: Čepno; minimumElevationInMeters: 555; maximumElevationInMeters: 555; decimalLatitude: 45.6735; decimalLongitude: 14.1068; **Event:** eventDate: 2012-07-22; habitat: grassland

#### Pholcus phalangioides

(Fuesslin, 1775)

##### Materials

- **Type status:** Other material. **Occurrence:** recordedBy: Kuntner, Čandek; sex: 1 female, 1 male; **Location:** locationID: SI50; country: Slovenia; locality: Sp. Prapreče; minimumElevationInMeters: 351; maximumElevationInMeters: 351; decimalLatitude: 46.1620; decimalLongitude: 14.6933; **Event:** eventDate: 2010-08-03/2012-05-28; habitat: house and surroundings
- **Type status:** Other material. **Occurrence:** recordedBy: Kostanjšek, RTŠB 2011; sex: 1 female; **Location:** locationID: SI68; country: Slovenia; locality: Sv. Jurij ob Ščavnici; minimumElevationInMeters: 235; maximumElevationInMeters: 235; decimalLatitude: 46.5687; decimalLongitude: 16.0223; **Event:** eventDate: 2011-07-22; habitat: school

#### Psilochorus simoni

(Berland, 1911)

##### Materials

- **Type status:** Other material. **Occurrence:** recordedBy: Kuntner, Čandek; sex: 1 female; **Location:** locationID: SI50; country: Slovenia; locality: Sp. Prapreče; minimumElevationInMeters: 351; maximumElevationInMeters: 351; decimalLatitude: 46.1620; decimalLongitude: 14.6933; **Event:** eventDate: 2010-08-03/2012-05-28; habitat: house and surroundings

#### Pisauridae

Simon, 1890

#### Pisaura mirabilis

(Clerck, 1757)

##### Materials

- **Type status:** Other material. **Occurrence:** recordedBy: Kuntner, Gregorič, Čandek; sex: 1 male; **Location:** locationID: CH06; country: Switzerland; locality: Bernese Alps, Kandersteg; minimumElevationInMeters: 1677; maximumElevationInMeters: 1677; decimalLatitude: 46.5020; decimalLongitude: 7.6992; **Event:** eventDate: 2011-07-07; habitat: alpine meadow
- **Type status:** Other material. **Occurrence:** recordedBy: Čandek; sex: 1 female, 1 male; **Location:** locationID: SI38; country: Slovenia; locality: Poreče; minimumElevationInMeters: 135; maximumElevationInMeters: 135; decimalLatitude: 45.8188; decimalLongitude: 13.9692; **Event:** eventDate: 2011-05-08; habitat: grassland
- **Type status:** Other material. **Occurrence:** recordedBy: Kuntner, Gregorič, Čandek, Kralj-Fišer, Cheng; sex: 1 female; **Location:** locationID: SI41; country: Slovenia; locality: Socerb, Osp; minimumElevationInMeters: 116; maximumElevationInMeters: 116; decimalLatitude: 45.5819; decimalLongitude: 13.8558; **Event:** eventDate: 2012-06-07; habitat: trail from Socerb to Osp
- **Type status:** Other material. **Occurrence:** recordedBy: Čandek; sex: 1 female, 2 males; **Location:** locationID: SI43; country: Slovenia; locality: Vipava; minimumElevationInMeters: 114; maximumElevationInMeters: 114; decimalLatitude: 45.8282; decimalLongitude: 13.9594; **Event:** eventDate: 2011-05-08; habitat: grassland
- **Type status:** Other material. **Occurrence:** recordedBy: Gregorič, Čandek; sex: 1 female, 2 males; **Location:** locationID: SI53; country: Slovenia; locality: Dinaric Karst, Griže; minimumElevationInMeters: 434; maximumElevationInMeters: 434; decimalLatitude: 45.7548; decimalLongitude: 13.9495; **Event:** eventDate: 2011-05-10/2011-06-21; habitat: grassland
- **Type status:** Other material. **Occurrence:** recordedBy: Čandek; sex: 1 male; **Location:** locationID: SI58; country: Slovenia; locality: Budanje; minimumElevationInMeters: 243; maximumElevationInMeters: 243; decimalLatitude: 45.8743; decimalLongitude: 13.9497; **Event:** eventDate: 2011-05-07; habitat: school and surroundings
- **Type status:** Other material. **Occurrence:** recordedBy: Čandek; sex: 2 females, 1 male; **Location:** locationID: SI60; country: Slovenia; locality: Budanje; minimumElevationInMeters: 295; maximumElevationInMeters: 295; decimalLatitude: 45.8799; decimalLongitude: 13.9459; **Event:** eventDate: 2011-05-07; habitat: forest clearing
- **Type status:** Other material. **Occurrence:** recordedBy: Čandek; sex: 1 female, 4 males; **Location:** locationID: SI61; country: Slovenia; locality: Sekirišče; minimumElevationInMeters: 750; maximumElevationInMeters: 750; decimalLatitude: 45.8631; decimalLongitude: 14.5367; **Event:** eventDate: 2011-06-23/2012-06-21; habitat: house, grassland, overgrowth

#### Salticidae

Blackwall, 1841

#### Evarcha arcuata

(Clerck, 1757)

##### Materials

- **Type status:** Other material. **Occurrence:** recordedBy: Kuntner, Gregorič, Čandek; sex: 2 females; **Location:** locationID: CH23; country: Switzerland; locality: Grison Alps, Alp Flix, Salategnas; minimumElevationInMeters: 1900; maximumElevationInMeters: 1900; decimalLatitude: 46.5141; decimalLongitude: 9.6448; **Event:** eventDate: 2011-07-12; habitat: forest opening, grass and shrubs
- **Type status:** Other material. **Occurrence:** recordedBy: Kuntner, Gregorič, Čandek; sex: 1 male; **Location:** locationID: CH30; country: Switzerland; locality: Grison Alps, Alp Flix - Lai Flix; minimumElevationInMeters: 1967; maximumElevationInMeters: 1967; decimalLatitude: 46.5358; decimalLongitude: 9.6409; **Event:** eventDate: 2011-07-16; habitat: next to alpine lake
- **Type status:** Other material. **Occurrence:** recordedBy: Kostanjšek, RTŠB 2011; sex: 1 female; **Location:** locationID: SI01; country: Slovenia; locality: Biš; minimumElevationInMeters: 225; maximumElevationInMeters: 225; decimalLatitude: 46.5374; decimalLongitude: 15.8963; **Event:** eventDate: 2011-07-22; habitat: forest
- **Type status:** Other material. **Occurrence:** recordedBy: Čandek; sex: 4 females, 1 male; **Location:** locationID: SI38; country: Slovenia; locality: Poreče; minimumElevationInMeters: 135; maximumElevationInMeters: 135; decimalLatitude: 45.8188; decimalLongitude: 13.9692; **Event:** eventDate: 2011-05-08; habitat: grassland
- **Type status:** Other material. **Occurrence:** recordedBy: Kuntner, Gregorič, Lokovšek; sex: 1 male; **Location:** locationID: SI39; country: Slovenia; locality: Primostek; minimumElevationInMeters: 157; maximumElevationInMeters: 157; decimalLatitude: 45.6299; decimalLongitude: 15.2997; **Event:** eventDate: 2010-08-24; habitat: grassland
- **Type status:** Other material. **Occurrence:** recordedBy: Kuntner, Lokovšek; sex: 1 female, 1 male; **Location:** locationID: SI40; country: Slovenia; locality: Slavnik; minimumElevationInMeters: 816; maximumElevationInMeters: 816; decimalLatitude: 45.5499; decimalLongitude: 13.9619; **Event:** eventDate: 2010-08-26; habitat: grassland and forest
- **Type status:** Other material. **Occurrence:** recordedBy: Čandek; sex: 2 females; **Location:** locationID: SI43; country: Slovenia; locality: Vipava; minimumElevationInMeters: 114; maximumElevationInMeters: 114; decimalLatitude: 45.8282; decimalLongitude: 13.9594; **Event:** eventDate: 2011-05-08; habitat: grassland
- **Type status:** Other material. **Occurrence:** recordedBy: Gregorič, Čandek, Kralj-Fišer; sex: 1 female, 1 male; **Location:** locationID: SI52; country: Slovenia; locality: Dinaric Karst, Griže; minimumElevationInMeters: 484; maximumElevationInMeters: 484; decimalLatitude: 45.7506; decimalLongitude: 13.9509; **Event:** eventDate: 2011-04-04/05-10; habitat: overgrowth
- **Type status:** Other material. **Occurrence:** recordedBy: Gregorič, Čandek; sex: 3 females, 2 males; **Location:** locationID: SI53; country: Slovenia; locality: Dinaric Karst, Griže; minimumElevationInMeters: 434; maximumElevationInMeters: 434; decimalLatitude: 45.7548; decimalLongitude: 13.9495; **Event:** eventDate: 2011-05-10/2011-06-21; habitat: grassland
- **Type status:** Other material. **Occurrence:** recordedBy: Gregorič, Čandek, Kralj-Fišer; sex: 2 males; **Location:** locationID: SI56; country: Slovenia; locality: Dinaric Karst, Novelo; minimumElevationInMeters: 358; maximumElevationInMeters: 359; decimalLatitude: 45.8533; decimalLongitude: 13.6552; **Event:** eventDate: 2011-04-04/05-10; habitat: overgrowth
- **Type status:** Other material. **Occurrence:** recordedBy: Čandek; sex: 1 female, 1 male; **Location:** locationID: SI60; country: Slovenia; locality: Budanje; minimumElevationInMeters: 295; maximumElevationInMeters: 295; decimalLatitude: 45.8799; decimalLongitude: 13.9459; **Event:** eventDate: 2011-05-07; habitat: forest clearing
- **Type status:** Other material. **Occurrence:** recordedBy: Čandek; sex: 6 females, 3 males; **Location:** locationID: SI61; country: Slovenia; locality: Sekirišče; minimumElevationInMeters: 750; maximumElevationInMeters: 750; decimalLatitude: 45.8631; decimalLongitude: 14.5367; **Event:** eventDate: 2011-06-23/2012-06-21; habitat: house, grassland, overgrowth

#### Evarcha falcata

(Clerck, 1757)

##### Materials

- **Type status:** Other material. **Occurrence:** recordedBy: Kostanjšek, RTŠB 2011; sex: 1 male; **Location:** locationID: SI01; country: Slovenia; locality: Biš; minimumElevationInMeters: 225; maximumElevationInMeters: 225; decimalLatitude: 46.5374; decimalLongitude: 15.8963; **Event:** eventDate: 2011-07-22; habitat: forest
- **Type status:** Other material. **Occurrence:** recordedBy: Kuntner, Gregorič, Lokovšek; sex: 1 female; **Location:** locationID: SI39; country: Slovenia; locality: Primostek; minimumElevationInMeters: 157; maximumElevationInMeters: 157; decimalLatitude: 45.6299; decimalLongitude: 15.2997; **Event:** eventDate: 2010-08-24; habitat: grassland
- **Type status:** Other material. **Occurrence:** recordedBy: Gregorič, Čandek, Kralj-Fišer; sex: 1 female, 1 male; **Location:** locationID: SI55; country: Slovenia; locality: Dinaric Karst, Lokvice; minimumElevationInMeters: 273; maximumElevationInMeters: 275; decimalLatitude: 45.8659; decimalLongitude: 13.6102; **Event:** eventDate: 2011-04-04/05-10; habitat: overgrowth
- **Type status:** Other material. **Occurrence:** recordedBy: Gregorič, Čandek, Kralj-Fišer; sex: 1 female, 1 male; **Location:** locationID: SI56; country: Slovenia; locality: Dinaric Karst, Novelo; minimumElevationInMeters: 358; maximumElevationInMeters: 359; decimalLatitude: 45.8533; decimalLongitude: 13.6552; **Event:** eventDate: 2011-04-04/05-10; habitat: overgrowth
- **Type status:** Other material. **Occurrence:** recordedBy: Čandek; sex: 2 females; **Location:** locationID: SI61; country: Slovenia; locality: Sekirišče; minimumElevationInMeters: 750; maximumElevationInMeters: 750; decimalLatitude: 45.8631; decimalLongitude: 14.5367; **Event:** eventDate: 2011-06-23/2012-06-21; habitat: house, grassland, overgrowth

#### Evarcha jucunda

(Lucas, 1846)

##### Materials

- **Type status:** Other material. **Occurrence:** recordedBy: Kuntner, Gregorič, Čandek, Kralj-Fišer, Cheng; sex: 1 male; **Location:** locationID: SI41; country: Slovenia; locality: Socerb, Osp; minimumElevationInMeters: 116; maximumElevationInMeters: 116; decimalLatitude: 45.5819; decimalLongitude: 13.8558; **Event:** eventDate: 2012-06-07; habitat: trail from Socerb to Osp

#### Evarcha laetabunda

(C. L. Koch, 1846)

##### Materials

- **Type status:** Other material. **Occurrence:** recordedBy: Čandek; sex: 2 females; **Location:** locationID: SI38; country: Slovenia; locality: Poreče; minimumElevationInMeters: 135; maximumElevationInMeters: 135; decimalLatitude: 45.8188; decimalLongitude: 13.9692; **Event:** eventDate: 2011-05-08; habitat: grassland
- **Type status:** Other material. **Occurrence:** recordedBy: Gregorič, Čandek, Kralj-Fišer; sex: 1 male; **Location:** locationID: SI52; country: Slovenia; locality: Dinaric Karst, Griže; minimumElevationInMeters: 484; maximumElevationInMeters: 484; decimalLatitude: 45.7506; decimalLongitude: 13.9509; **Event:** eventDate: 2011-04-04/05-10; habitat: overgrowth
- **Type status:** Other material. **Occurrence:** recordedBy: Gregorič, Čandek; sex: 2 females; **Location:** locationID: SI53; country: Slovenia; locality: Dinaric Karst, Griže; minimumElevationInMeters: 434; maximumElevationInMeters: 434; decimalLatitude: 45.7548; decimalLongitude: 13.9495; **Event:** eventDate: 2011-05-10/2011-06-21; habitat: grassland
- **Type status:** Other material. **Occurrence:** recordedBy: Gregorič, Čandek, Kralj-Fišer; sex: 5 females, 2 males; **Location:** locationID: SI56; country: Slovenia; locality: Dinaric Karst, Novelo; minimumElevationInMeters: 358; maximumElevationInMeters: 359; decimalLatitude: 45.8533; decimalLongitude: 13.6552; **Event:** eventDate: 2011-04-04/05-10; habitat: overgrowth

#### Evarcha michailovi

Logunov, 1992

##### Materials

- **Type status:** Other material. **Occurrence:** recordedBy: Gregorič, Čandek, Kralj-Fišer; sex: 1 male; **Location:** locationID: SI52; country: Slovenia; locality: Dinaric Karst, Griže; minimumElevationInMeters: 484; maximumElevationInMeters: 484; decimalLatitude: 45.7506; decimalLongitude: 13.9509; **Event:** eventDate: 2011-04-04/05-10; habitat: overgrowth

#### Hasarius adansoni

(Audouin, 1826)

##### Materials

- **Type status:** Other material. **Occurrence:** recordedBy: Čandek; sex: 1 male; **Location:** locationID: SI51; country: Slovenia; locality: Ljubljana, Nove Jarše; minimumElevationInMeters: 294; maximumElevationInMeters: 294; decimalLatitude: 46.0712; decimalLongitude: 14.5403; **Event:** eventDate: 2011-06-10/08-31; habitat: house

#### Heliophanus aeneus

(Hahn, 1832)

##### Materials

- **Type status:** Other material. **Occurrence:** recordedBy: Kostanjšek, RTŠB 2012; sex: 1 female, 1 male; **Location:** locationID: SI21; country: Slovenia; locality: Jurišče; minimumElevationInMeters: 730; maximumElevationInMeters: 730; decimalLatitude: 45.6735; decimalLongitude: 14.3093; **Event:** eventDate: 2012-07-23; habitat: overgrown grassland

#### Heliophanus cupreus

(Walckenaer, 1802)

##### Materials

- **Type status:** Other material. **Occurrence:** recordedBy: Kuntner, Gregorič, Čandek; sex: 2 males; **Location:** locationID: CH12; country: Switzerland; locality: Bernese Alps, Nessental; minimumElevationInMeters: 930; maximumElevationInMeters: 930; decimalLatitude: 46.7213; decimalLongitude: 8.3039; **Event:** eventDate: 2011-07-10; habitat: grassland and loan trees
- **Type status:** Other material. **Occurrence:** recordedBy: Čandek; sex: 1 male; **Location:** locationID: SI38; country: Slovenia; locality: Poreče; minimumElevationInMeters: 135; maximumElevationInMeters: 135; decimalLatitude: 45.8188; decimalLongitude: 13.9692; **Event:** eventDate: 2011-05-08; habitat: grassland
- **Type status:** Other material. **Occurrence:** recordedBy: Kuntner, Gregorič, Čandek, Kralj-Fišer, Cheng; sex: 1 male; **Location:** locationID: SI41; country: Slovenia; locality: Socerb, Osp; minimumElevationInMeters: 116; maximumElevationInMeters: 116; decimalLatitude: 45.5819; decimalLongitude: 13.8558; **Event:** eventDate: 2012-06-07; habitat: trail from Socerb to Osp
- **Type status:** Other material. **Occurrence:** recordedBy: Gregorič, Čandek, Kralj-Fišer; sex: 2 females; **Location:** locationID: SI52; country: Slovenia; locality: Dinaric Karst, Griže; minimumElevationInMeters: 484; maximumElevationInMeters: 484; decimalLatitude: 45.7506; decimalLongitude: 13.9509; **Event:** eventDate: 2011-04-04/05-10; habitat: overgrowth
- **Type status:** Other material. **Occurrence:** recordedBy: Gregorič, Čandek; sex: 4 females, 4 males; **Location:** locationID: SI53; country: Slovenia; locality: Dinaric Karst, Griže; minimumElevationInMeters: 434; maximumElevationInMeters: 434; decimalLatitude: 45.7548; decimalLongitude: 13.9495; **Event:** eventDate: 2011-05-10/2011-06-21; habitat: grassland
- **Type status:** Other material. **Occurrence:** recordedBy: Gregorič, Čandek, Kralj-Fišer; sex: 5 females; **Location:** locationID: SI55; country: Slovenia; locality: Dinaric Karst, Lokvice; minimumElevationInMeters: 273; maximumElevationInMeters: 275; decimalLatitude: 45.8659; decimalLongitude: 13.6102; **Event:** eventDate: 2011-04-04/05-10; habitat: overgrowth
- **Type status:** Other material. **Occurrence:** recordedBy: Gregorič, Čandek, Kralj-Fišer; sex: 2 females, 3 males; **Location:** locationID: SI56; country: Slovenia; locality: Dinaric Karst, Novelo; minimumElevationInMeters: 358; maximumElevationInMeters: 359; decimalLatitude: 45.8533; decimalLongitude: 13.6552; **Event:** eventDate: 2011-04-04/05-10; habitat: overgrowth
- **Type status:** Other material. **Occurrence:** recordedBy: Gregorič, Čandek; sex: 1 male; **Location:** locationID: SI57; country: Slovenia; locality: Dinaric Karst, Novelo; minimumElevationInMeters: 325; maximumElevationInMeters: 325; decimalLatitude: 45.8482; decimalLongitude: 13.6584; **Event:** eventDate: 2011-05-10; habitat: grassland
- **Type status:** Other material. **Occurrence:** recordedBy: Čandek; sex: 8 females, 4 males; **Location:** locationID: SI60; country: Slovenia; locality: Budanje; minimumElevationInMeters: 295; maximumElevationInMeters: 295; decimalLatitude: 45.8799; decimalLongitude: 13.9459; **Event:** eventDate: 2011-05-07; habitat: forest clearing
- **Type status:** Other material. **Occurrence:** recordedBy: Čandek; sex: 1 male; **Location:** locationID: SI61; country: Slovenia; locality: Sekirišče; minimumElevationInMeters: 750; maximumElevationInMeters: 750; decimalLatitude: 45.8631; decimalLongitude: 14.5367; **Event:** eventDate: 2011-06-23/2012-06-21; habitat: house, grassland, overgrowth

#### Heliophanus flavipes

(Hahn, 1832)

##### Materials

- **Type status:** Other material. **Occurrence:** recordedBy: Kostanjšek, RTŠB 2011; sex: 1 female; **Location:** locationID: SI20; country: Slovenia; locality: Dragotinci; minimumElevationInMeters: 225; maximumElevationInMeters: 225; decimalLatitude: 46.5885; decimalLongitude: 16.0297; **Event:** eventDate: 2011-07-27; habitat: grassland
- **Type status:** Other material. **Occurrence:** recordedBy: Čandek; sex: 3 females; **Location:** locationID: SI38; country: Slovenia; locality: Poreče; minimumElevationInMeters: 135; maximumElevationInMeters: 135; decimalLatitude: 45.8188; decimalLongitude: 13.9692; **Event:** eventDate: 2011-05-08; habitat: grassland
- **Type status:** Other material. **Occurrence:** recordedBy: Čandek; sex: 3 females; **Location:** locationID: SI43; country: Slovenia; locality: Vipava; minimumElevationInMeters: 114; maximumElevationInMeters: 114; decimalLatitude: 45.8282; decimalLongitude: 13.9594; **Event:** eventDate: 2011-05-08; habitat: grassland
- **Type status:** Other material. **Occurrence:** recordedBy: Čandek; sex: 1 male; **Location:** locationID: SI61; country: Slovenia; locality: Sekirišče; minimumElevationInMeters: 750; maximumElevationInMeters: 750; decimalLatitude: 45.8631; decimalLongitude: 14.5367; **Event:** eventDate: 2011-06-23/2012-06-21; habitat: house, grassland, overgrowth

#### Heliophanus kochii

Simon, 1868

##### Materials

- **Type status:** Other material. **Occurrence:** recordedBy: Kuntner, Gregorič, Čandek, Kralj-Fišer, Cheng; sex: 1 male; **Location:** locationID: SI41; country: Slovenia; locality: Socerb, Osp; minimumElevationInMeters: 116; maximumElevationInMeters: 116; decimalLatitude: 45.5819; decimalLongitude: 13.8558; **Event:** eventDate: 2012-06-07; habitat: trail from Socerb to Osp

#### Icius subinermis

Simon, 1937

##### Materials

- **Type status:** Other material. **Occurrence:** recordedBy: Kuntner, Čandek; sex: 1 male; **Location:** locationID: SI50; country: Slovenia; locality: Sp. Prapreče; minimumElevationInMeters: 351; maximumElevationInMeters: 351; decimalLatitude: 46.1620; decimalLongitude: 14.6933; **Event:** eventDate: 2010-08-03/2012-05-28; habitat: house and surroundings
- **Type status:** Other material. **Occurrence:** recordedBy: Čandek; sex: 2 females; **Location:** locationID: SI51; country: Slovenia; locality: Ljubljana, Nove Jarše; minimumElevationInMeters: 294; maximumElevationInMeters: 294; decimalLatitude: 46.0712; decimalLongitude: 14.5403; **Event:** eventDate: 2011-06-10/08-31; habitat: house

#### Leptorchetes berolinensis

(C. L. Koch, 1846)

##### Materials

- **Type status:** Other material. **Occurrence:** recordedBy: Kuntner, Čandek; sex: 1 male; **Location:** locationID: SI50; country: Slovenia; locality: Sp. Prapreče; minimumElevationInMeters: 351; maximumElevationInMeters: 351; decimalLatitude: 46.1620; decimalLongitude: 14.6933; **Event:** eventDate: 2010-08-03/2012-05-28; habitat: house and surroundings

#### Macaroeris nidicolens

(Walckenaer, 1802)

##### Materials

- **Type status:** Other material. **Occurrence:** recordedBy: Kuntner, Gregorič, Čandek, Kralj-Fišer, Cheng; sex: 2 females; **Location:** locationID: SI41; country: Slovenia; locality: Socerb, Osp; minimumElevationInMeters: 116; maximumElevationInMeters: 116; decimalLatitude: 45.5819; decimalLongitude: 13.8558; **Event:** eventDate: 2012-06-07; habitat: trail from Socerb to Osp
- **Type status:** Other material. **Occurrence:** recordedBy: Čandek; sex: 1 male; **Location:** locationID: SI46; country: Slovenia; locality: Šešče pri Preboldu; minimumElevationInMeters: 284; maximumElevationInMeters: 285; decimalLatitude: 46.2356; decimalLongitude: 15.1228; **Event:** eventDate: 2011-06-13/2012-06-22; habitat: house and surroundings

#### Marpissa muscosa

(Clerck, 1757)

##### Materials

- **Type status:** Other material. **Occurrence:** recordedBy: Kuntner, Gregorič, Lokovšek; sex: 1 male; **Location:** locationID: SI39; country: Slovenia; locality: Primostek; minimumElevationInMeters: 157; maximumElevationInMeters: 157; decimalLatitude: 45.6299; decimalLongitude: 15.2997; **Event:** eventDate: 2010-08-24; habitat: grassland

#### Marpissa nivoyi

(Lucas, 1846)

##### Materials

- **Type status:** Other material. **Occurrence:** recordedBy: Kuntner, Gregorič, Čandek, Kralj-Fišer, Cheng; sex: 1 female; **Location:** locationID: SI41; country: Slovenia; locality: Socerb, Osp; minimumElevationInMeters: 116; maximumElevationInMeters: 116; decimalLatitude: 45.5819; decimalLongitude: 13.8558; **Event:** eventDate: 2012-06-07; habitat: trail from Socerb to Osp
- **Type status:** Other material. **Occurrence:** recordedBy: Gregorič, Čandek, Kralj-Fišer; sex: 3 females, 3 males; **Location:** locationID: SI52; country: Slovenia; locality: Dinaric Karst, Griže; minimumElevationInMeters: 484; maximumElevationInMeters: 484; decimalLatitude: 45.7506; decimalLongitude: 13.9509; **Event:** eventDate: 2011-04-04/05-10; habitat: overgrowth
- **Type status:** Other material. **Occurrence:** recordedBy: Gregorič, Čandek, Kralj-Fišer; sex: 2 females, 1 male; **Location:** locationID: SI56; country: Slovenia; locality: Dinaric Karst, Novelo; minimumElevationInMeters: 358; maximumElevationInMeters: 359; decimalLatitude: 45.8533; decimalLongitude: 13.6552; **Event:** eventDate: 2011-04-04/05-10; habitat: overgrowth

#### Myrmarachne formicaria

(De Geer, 1778)

##### Materials

- **Type status:** Other material. **Occurrence:** recordedBy: Čandek; sex: 1 female; **Location:** locationID: SI33; country: Slovenia; locality: Ljubljana, Biotechnical faculty; minimumElevationInMeters: 297; maximumElevationInMeters: 297; decimalLatitude: 46.0513; decimalLongitude: 14.4700; **Event:** eventDate: 2012-05-09; habitat: house and surroundings

#### Neon reticulatus

(Blackwall, 1853)

##### Materials

- **Type status:** Other material. **Occurrence:** recordedBy: Kostanjšek, RTŠB 2011; sex: 1 female; **Location:** locationID: SI04; country: Slovenia; locality: Cerkvenjak; minimumElevationInMeters: 230; maximumElevationInMeters: 230; decimalLatitude: 46.5641; decimalLongitude: 15.9863; **Event:** eventDate: 2011-07-22; habitat: grassland

#### Pellenes seriatus

(Thorell, 1875)

##### Materials

- **Type status:** Other material. **Occurrence:** recordedBy: Čandek; sex: 1 female; **Location:** locationID: SI38; country: Slovenia; locality: Poreče; minimumElevationInMeters: 135; maximumElevationInMeters: 135; decimalLatitude: 45.8188; decimalLongitude: 13.9692; **Event:** eventDate: 2011-05-08; habitat: grassland
- **Type status:** Other material. **Occurrence:** recordedBy: Gregorič, Čandek, Kralj-Fišer; sex: 1 male; **Location:** locationID: SI52; country: Slovenia; locality: Dinaric Karst, Griže; minimumElevationInMeters: 484; maximumElevationInMeters: 484; decimalLatitude: 45.7506; decimalLongitude: 13.9509; **Event:** eventDate: 2011-04-04/05-10; habitat: overgrowth
- **Type status:** Other material. **Occurrence:** recordedBy: Gregorič, Čandek; sex: 1 female; **Location:** locationID: SI53; country: Slovenia; locality: Dinaric Karst, Griže; minimumElevationInMeters: 434; maximumElevationInMeters: 434; decimalLatitude: 45.7548; decimalLongitude: 13.9495; **Event:** eventDate: 2011-05-10/2011-06-21; habitat: grassland
- **Type status:** Other material. **Occurrence:** recordedBy: Gregorič, Čandek, Kralj-Fišer; sex: 1 female; **Location:** locationID: SI55; country: Slovenia; locality: Dinaric Karst, Lokvice; minimumElevationInMeters: 273; maximumElevationInMeters: 275; decimalLatitude: 45.8659; decimalLongitude: 13.6102; **Event:** eventDate: 2011-04-04/05-10; habitat: overgrowth

#### Pellenes tripunctatus

(Walckenaer, 1802)

##### Materials

- **Type status:** Other material. **Occurrence:** recordedBy: Gregorič, Čandek, Kralj-Fišer; sex: 1 male; **Location:** locationID: SI56; country: Slovenia; locality: Dinaric Karst, Novelo; minimumElevationInMeters: 358; maximumElevationInMeters: 359; decimalLatitude: 45.8533; decimalLongitude: 13.6552; **Event:** eventDate: 2011-04-04/05-10; habitat: overgrowth

#### Philaeus chrysops

(Poda, 1761)

##### Materials

- **Type status:** Other material. **Occurrence:** recordedBy: Kostanjšek, RTŠB 2012; sex: 1 female; **Location:** locationID: SI25; country: Slovenia; locality: Dolnja Košana; minimumElevationInMeters: 435; maximumElevationInMeters: 435; decimalLatitude: 45.6646; decimalLongitude: 14.1350; **Event:** eventDate: 2012-07-27; habitat: grassland
- **Type status:** Other material. **Occurrence:** recordedBy: Gregorič, Čandek; sex: 1 male; **Location:** locationID: SI53; country: Slovenia; locality: Dinaric Karst, Griže; minimumElevationInMeters: 434; maximumElevationInMeters: 434; decimalLatitude: 45.7548; decimalLongitude: 13.9495; **Event:** eventDate: 2011-05-10/2011-06-21; habitat: grassland

#### Pseudeuophrys lanigera

(Simon, 1871)

##### Materials

- **Type status:** Other material. **Occurrence:** recordedBy: Kuntner, Čandek; sex: 1 female; **Location:** locationID: SI50; country: Slovenia; locality: Sp. Prapreče; minimumElevationInMeters: 351; maximumElevationInMeters: 351; decimalLatitude: 46.1620; decimalLongitude: 14.6933; **Event:** eventDate: 2010-08-03/2012-05-28; habitat: house and surroundings

#### Saitis barbipes

(Simon, 1868)

##### Materials

- **Type status:** Other material. **Occurrence:** recordedBy: Kuntner, Gregorič, Čandek, Kralj-Fišer, Cheng; sex: 7 females, 6 males; **Location:** locationID: SI41; country: Slovenia; locality: Socerb, Osp; minimumElevationInMeters: 116; maximumElevationInMeters: 116; decimalLatitude: 45.5819; decimalLongitude: 13.8558; **Event:** eventDate: 2012-06-07; habitat: trail from Socerb to Osp
- **Type status:** Other material. **Occurrence:** recordedBy: Gregorič, Čandek, Kralj-Fišer; sex: 1 male; **Location:** locationID: SI56; country: Slovenia; locality: Dinaric Karst, Novelo; minimumElevationInMeters: 358; maximumElevationInMeters: 359; decimalLatitude: 45.8533; decimalLongitude: 13.6552; **Event:** eventDate: 2011-04-04/05-10; habitat: overgrowth
- **Type status:** Other material. **Occurrence:** recordedBy: Čandek; sex: 1 male; **Location:** locationID: SI59; country: Slovenia; locality: Budanje; minimumElevationInMeters: 305; maximumElevationInMeters: 305; decimalLatitude: 45.8797; decimalLongitude: 13.9468; **Event:** eventDate: 2011-05-07; habitat: forest

#### Salticus scenius

(Clerck, 1757)

##### Materials

- **Type status:** Other material. **Occurrence:** recordedBy: Kuntner, Gregorič, Čandek; sex: 1 female; **Location:** locationID: CH18; country: Switzerland; locality: Grison Alps, Alp Flix, Salategnas; minimumElevationInMeters: 1900; maximumElevationInMeters: 1900; decimalLatitude: 46.5166; decimalLongitude: 9.6523; **Event:** eventDate: 2011-07-12/19; habitat: around house
- **Type status:** Other material. **Occurrence:** recordedBy: Čandek; sex: 1 male; **Location:** locationID: SI33; country: Slovenia; locality: Ljubljana, Biotechnical faculty; minimumElevationInMeters: 297; maximumElevationInMeters: 297; decimalLatitude: 46.0513; decimalLongitude: 14.4700; **Event:** eventDate: 2012-05-09; habitat: house and surroundings
- **Type status:** Other material. **Occurrence:** recordedBy: Gregorič, Kuntner, Čandek; sex: 1 female; **Location:** locationID: SI48; country: Slovenia; locality: Ljubljana, center; minimumElevationInMeters: 291; maximumElevationInMeters: 291; decimalLatitude: 46.0434; decimalLongitude: 14.5041; **Event:** eventDate: 2011-05-24/2012-06-19; habitat: house

#### Sibianor aurocinctus

(Ohlert, 1865)

##### Materials

- **Type status:** Other material. **Occurrence:** recordedBy: Kuntner, Gregorič, Čandek; sex: 3 males; **Location:** locationID: CH12; country: Switzerland; locality: Bernese Alps, Nessental; minimumElevationInMeters: 930; maximumElevationInMeters: 930; decimalLatitude: 46.7213; decimalLongitude: 8.3039; **Event:** eventDate: 2011-07-10; habitat: grassland and loan trees

#### Sitticus rupicola

(C. L. Koch, 1837)

##### Materials

- **Type status:** Other material. **Occurrence:** recordedBy: Kuntner, Gregorič, Čandek; sex: 1 male; **Location:** locationID: CH01; country: Switzerland; locality: Bernese Alps, Gasteretal; minimumElevationInMeters: 1662; maximumElevationInMeters: 1662; decimalLatitude: 46.4457; decimalLongitude: 7.7413; **Event:** eventDate: 2011-07-07; habitat: alpine meadow
- **Type status:** Other material. **Occurrence:** recordedBy: Kuntner, Gregorič, Čandek; sex: 2 females, 1 male; **Location:** locationID: CH23; country: Switzerland; locality: Grison Alps, Alp Flix, Salategnas; minimumElevationInMeters: 1900; maximumElevationInMeters: 1900; decimalLatitude: 46.5141; decimalLongitude: 9.6448; **Event:** eventDate: 2011-07-12; habitat: forest opening, grass and shrubs
- **Type status:** Other material. **Occurrence:** recordedBy: Kuntner, Gregorič, Čandek; sex: 1 male; **Location:** locationID: CH25; country: Switzerland; locality: Grison Alps, Alp Flix, Salategnas; minimumElevationInMeters: 1950; maximumElevationInMeters: 1950; decimalLatitude: 46.5159; decimalLongitude: 9.6496; **Event:** eventDate: 2011-07-12/16; habitat: meadow and shrubs at stream

#### Scytodidae

Blackwall, 1864

#### Scytodes thoracica

(Latreille, 1802)

##### Materials

- **Type status:** Other material. **Occurrence:** recordedBy: Kuntner, Gregorič, Čandek, Kralj-Fišer, Cheng; sex: 1 female2 juveniles; **Location:** locationID: SI41; country: Slovenia; locality: Socerb, Osp; minimumElevationInMeters: 116; maximumElevationInMeters: 116; decimalLatitude: 45.5819; decimalLongitude: 13.8558; **Event:** eventDate: 2012-06-07; habitat: trail from Socerb to Osp
- **Type status:** Other material. **Occurrence:** recordedBy: Kuntner, Čandek; sex: 1 male; **Location:** locationID: SI50; country: Slovenia; locality: Sp. Prapreče; minimumElevationInMeters: 351; maximumElevationInMeters: 351; decimalLatitude: 46.1620; decimalLongitude: 14.6933; **Event:** eventDate: 2010-08-03/2012-05-28; habitat: house and surroundings

#### Segestriidae

Simon, 1893

#### Segestria senoculata

(Linnaeus, 1758)

##### Materials

- **Type status:** Other material. **Occurrence:** recordedBy: Kostanjšek, RTŠB 2012; sex: 1 female; **Location:** locationID: SI11; country: Slovenia; locality: Divača; minimumElevationInMeters: 460; maximumElevationInMeters: 460; decimalLatitude: 45.6835; decimalLongitude: 14.0166; **Event:** eventDate: 2012-07-22; habitat: grassland
- **Type status:** Other material. **Occurrence:** recordedBy: Kostanjšek, RTŠB 2012; sex: 1 female; **Location:** locationID: SI21; country: Slovenia; locality: Jurišče; minimumElevationInMeters: 730; maximumElevationInMeters: 730; decimalLatitude: 45.6735; decimalLongitude: 14.3093; **Event:** eventDate: 2012-07-23; habitat: overgrown grassland

#### Sparassidae

Bertkau, 1872

#### Micrommata virescens

(Clerck, 1757)

##### Materials

- **Type status:** Other material. **Occurrence:** recordedBy: Kuntner, Gregorič, Čandek; sex: 1 male; **Location:** locationID: CH05; country: Switzerland; locality: Bernese Alps, Gasteretal; minimumElevationInMeters: 1380; maximumElevationInMeters: 1380; decimalLatitude: 46.4674; decimalLongitude: 7.6640; **Event:** eventDate: 2011-07-07; habitat: river vegetation
- **Type status:** Other material. **Occurrence:** recordedBy: Kuntner, Gregorič, Čandek; sex: 1 female1 juvenile; **Location:** locationID: CH23; country: Switzerland; locality: Grison Alps, Alp Flix, Salategnas; minimumElevationInMeters: 1900; maximumElevationInMeters: 1900; decimalLatitude: 46.5141; decimalLongitude: 9.6448; **Event:** eventDate: 2011-07-12; habitat: forest opening, grass and shrubs
- **Type status:** Other material. **Occurrence:** recordedBy: Kuntner, Gregorič, Čandek; sex: 1 male, 1 juvenile; **Location:** locationID: CH25; country: Switzerland; locality: Grison Alps, Alp Flix, Salategnas; minimumElevationInMeters: 1950; maximumElevationInMeters: 1950; decimalLatitude: 46.5159; decimalLongitude: 9.6496; **Event:** eventDate: 2011-07-12/16; habitat: meadow and shrubs at stream
- **Type status:** Other material. **Occurrence:** recordedBy: Kuntner, Gregorič, Čandek; sex: 1 juvenile; **Location:** locationID: CH30; country: Switzerland; locality: Grison Alps, Alp Flix - Lai Flix; minimumElevationInMeters: 1967; maximumElevationInMeters: 1967; decimalLatitude: 46.5358; decimalLongitude: 9.6409; **Event:** eventDate: 2011-07-16; habitat: next to alpine lake
- **Type status:** Other material. **Occurrence:** recordedBy: Kuntner, Gregorič, Čandek; sex: 1 male; **Location:** locationID: CH32; country: Switzerland; locality: Grison Alps, Alp Flix, Salategnas; minimumElevationInMeters: 1955; maximumElevationInMeters: 1955; decimalLatitude: 46.5203; decimalLongitude: 9.6458; **Event:** eventDate: 2011-07-16; habitat: timberline forest, moss
- **Type status:** Other material. **Occurrence:** recordedBy: Čandek; sex: 1 male; **Location:** locationID: SI38; country: Slovenia; locality: Poreče; minimumElevationInMeters: 135; maximumElevationInMeters: 135; decimalLatitude: 45.8188; decimalLongitude: 13.9692; **Event:** eventDate: 2011-05-08; habitat: grassland
- **Type status:** Other material. **Occurrence:** recordedBy: Gregorič, Čandek, Kralj-Fišer; sex: 1 male; **Location:** locationID: SI52; country: Slovenia; locality: Dinaric Karst, Griže; minimumElevationInMeters: 484; maximumElevationInMeters: 484; decimalLatitude: 45.7506; decimalLongitude: 13.9509; **Event:** eventDate: 2011-04-04/05-10; habitat: overgrowth
- **Type status:** Other material. **Occurrence:** recordedBy: Gregorič, Čandek, Kralj-Fišer; sex: 1 female, 2 males; **Location:** locationID: SI55; country: Slovenia; locality: Dinaric Karst, Lokvice; minimumElevationInMeters: 273; maximumElevationInMeters: 275; decimalLatitude: 45.8659; decimalLongitude: 13.6102; **Event:** eventDate: 2011-04-04/05-10; habitat: overgrowth
- **Type status:** Other material. **Occurrence:** recordedBy: Gregorič, Čandek, Kralj-Fišer; sex: 1 female; **Location:** locationID: SI56; country: Slovenia; locality: Dinaric Karst, Novelo; minimumElevationInMeters: 358; maximumElevationInMeters: 359; decimalLatitude: 45.8533; decimalLongitude: 13.6552; **Event:** eventDate: 2011-04-04/05-10; habitat: overgrowth
- **Type status:** Other material. **Occurrence:** recordedBy: Čandek; sex: 1 male; **Location:** locationID: SI58; country: Slovenia; locality: Budanje; minimumElevationInMeters: 243; maximumElevationInMeters: 243; decimalLatitude: 45.8743; decimalLongitude: 13.9497; **Event:** eventDate: 2011-05-07; habitat: school and surroundings
- **Type status:** Other material. **Occurrence:** recordedBy: Čandek; sex: 1 female, 2 males; **Location:** locationID: SI60; country: Slovenia; locality: Budanje; minimumElevationInMeters: 295; maximumElevationInMeters: 295; decimalLatitude: 45.8799; decimalLongitude: 13.9459; **Event:** eventDate: 2011-05-07; habitat: forest clearing
- **Type status:** Other material. **Occurrence:** recordedBy: Čandek; sex: 1 female; **Location:** locationID: SI61; country: Slovenia; locality: Sekirišče; minimumElevationInMeters: 750; maximumElevationInMeters: 750; decimalLatitude: 45.8631; decimalLongitude: 14.5367; **Event:** eventDate: 2011-06-23/2012-06-21; habitat: house, grassland, overgrowth

#### Tetragnathidae

Menge, 1866

#### Meta menardi

(Latreille, 1804)

##### Materials

- **Type status:** Other material. **Occurrence:** recordedBy: Aljančič; sex: 1 female; **Location:** locationID: SI31; country: Slovenia; locality: Kranj, Tular cave; minimumElevationInMeters: 357; maximumElevationInMeters: 357; decimalLatitude: 46.2359; decimalLongitude: 14.3490; **Event:** eventDate: 2010-09-04; habitat: cave

#### Metellina mengei

(Blackwall, 1870)

##### Materials

- **Type status:** Other material. **Occurrence:** recordedBy: Kuntner, Gregorič, Čandek; sex: 2 females; **Location:** locationID: CH17; country: Switzerland; locality: Engadin, Bivio; minimumElevationInMeters: 1780; maximumElevationInMeters: 1780; decimalLatitude: 46.4753; decimalLongitude: 9.6469; **Event:** eventDate: 2011-07-11; habitat: forest and river edge
- **Type status:** Other material. **Occurrence:** recordedBy: Kuntner, Gregorič, Čandek; sex: 1 female, 1 male; **Location:** locationID: CH25; country: Switzerland; locality: Grison Alps, Alp Flix, Salategnas; minimumElevationInMeters: 1950; maximumElevationInMeters: 1950; decimalLatitude: 46.5159; decimalLongitude: 9.6496; **Event:** eventDate: 2011-07-12/16; habitat: meadow and shrubs at stream
- **Type status:** Other material. **Occurrence:** recordedBy: Kuntner, Gregorič, Čandek; sex: 1 female; **Location:** locationID: CH31; country: Switzerland; locality: Grison Alps, Alp Flix - Lai Neir; minimumElevationInMeters: 1910; maximumElevationInMeters: 1910; decimalLatitude: 46.5343; decimalLongitude: 9.6375; **Event:** eventDate: 2011-07-16; habitat: lake and swamp around forest

#### Metellina merianae

(Scopoli, 1763)

##### Materials

- **Type status:** Other material. **Occurrence:** recordedBy: Kuntner, Gregorič, Čandek; sex: 2 females; **Location:** locationID: CH02; country: Switzerland; locality: Bernese Alps, Gasteretal; minimumElevationInMeters: 1698; maximumElevationInMeters: 1698; decimalLatitude: 46.4486; decimalLongitude: 7.7438; **Event:** eventDate: 2011-07-07; habitat: spruce thicket and grass
- **Type status:** Other material. **Occurrence:** recordedBy: Kostanjšek, RTŠB 2011; sex: 1 female; **Location:** locationID: SI01; country: Slovenia; locality: Biš; minimumElevationInMeters: 225; maximumElevationInMeters: 225; decimalLatitude: 46.5374; decimalLongitude: 15.8963; **Event:** eventDate: 2011-07-22; habitat: forest
- **Type status:** Other material. **Occurrence:** recordedBy: Čandek; sex: 2 females, 1 male; **Location:** locationID: SI29; country: Slovenia; locality: Gradišče pri Lukovici, Gradiško jezero; minimumElevationInMeters: 360; maximumElevationInMeters: 360; decimalLatitude: 46.1626; decimalLongitude: 14.7127; **Event:** eventDate: 2011-10-06; habitat: lake edge
- **Type status:** Other material. **Occurrence:** recordedBy: Kuntner; sex: 1 male; **Location:** locationID: SI36; country: Slovenia; locality: Močilnik; minimumElevationInMeters: 318; maximumElevationInMeters: 318; decimalLatitude: 45.9547; decimalLongitude: 14.2925; **Event:** eventDate: 2011-10-02; habitat: forest edge
- **Type status:** Other material. **Occurrence:** recordedBy: Kuntner, Čandek; sex: 1 female, 1 male; **Location:** locationID: SI50; country: Slovenia; locality: Sp. Prapreče; minimumElevationInMeters: 351; maximumElevationInMeters: 351; decimalLatitude: 46.1620; decimalLongitude: 14.6933; **Event:** eventDate: 2010-08-03/2012-05-28; habitat: house and surroundings

#### Metellina segmentata

(Clerk, 1757)

#### Pachygnatha degeeri

Sundevall, 1830

##### Materials

- **Type status:** Other material. **Occurrence:** recordedBy: Kuntner, Gregorič, Čandek; sex: 2 males; **Location:** locationID: CH12; country: Switzerland; locality: Bernese Alps, Nessental; minimumElevationInMeters: 930; maximumElevationInMeters: 930; decimalLatitude: 46.7213; decimalLongitude: 8.3039; **Event:** eventDate: 2011-07-10; habitat: grassland and loan trees
- **Type status:** Other material. **Occurrence:** recordedBy: Kuntner, Gregorič, Čandek; sex: 4 males; **Location:** locationID: CH15; country: Switzerland; locality: Glarus Alps, near Affeier; minimumElevationInMeters: 817; maximumElevationInMeters: 817; decimalLatitude: 46.7606; decimalLongitude: 9.0933; **Event:** eventDate: 2011-07-10; habitat: meadow and forest
- **Type status:** Other material. **Occurrence:** recordedBy: Kostanjšek, RTŠB 2011; sex: 1 male; **Location:** locationID: SI05; country: Slovenia; locality: Sv. Jurij ob Ščavnici; minimumElevationInMeters: 190; maximumElevationInMeters: 190; decimalLatitude: 46.5509; decimalLongitude: 16.0451; **Event:** eventDate: 2011-07-22; habitat: overgrown river channel

#### Pachygnatha listeri

Sundevall, 1830

##### Materials

- **Type status:** Other material. **Occurrence:** recordedBy: Kuntner, Gregorič, Čandek; sex: 1 female; **Location:** locationID: CH15; country: Switzerland; locality: Glarus Alps, near Affeier; minimumElevationInMeters: 817; maximumElevationInMeters: 817; decimalLatitude: 46.7606; decimalLongitude: 9.0933; **Event:** eventDate: 2011-07-10; habitat: meadow and forest
- **Type status:** Other material. **Occurrence:** recordedBy: Kostanjšek, RTŠB 2011; sex: 1 male; **Location:** locationID: SI14; country: Slovenia; locality: Spodnji Velovlek; minimumElevationInMeters: 225; maximumElevationInMeters: 225; decimalLatitude: 46.4768; decimalLongitude: 15.9316; **Event:** eventDate: 2011-07-25; habitat: forest

#### Tetragnatha extensa

(Linnaeus, 1758)

##### Materials

- **Type status:** Other material. **Occurrence:** recordedBy: Čandek; sex: 1 female; **Location:** locationID: SI61; country: Slovenia; locality: Sekirišče; minimumElevationInMeters: 750; maximumElevationInMeters: 750; decimalLatitude: 45.8631; decimalLongitude: 14.5367; **Event:** eventDate: 2011-06-23/2012-06-21; habitat: house, grassland, overgrowth

#### Tetragnatha nigrita

Lendl, 1886

##### Materials

- **Type status:** Other material. **Occurrence:** recordedBy: Kuntner, Gregorič, Lokovšek; sex: 2 females, 1 male; **Location:** locationID: SI39; country: Slovenia; locality: Primostek; minimumElevationInMeters: 157; maximumElevationInMeters: 157; decimalLatitude: 45.6299; decimalLongitude: 15.2997; **Event:** eventDate: 2010-08-24; habitat: grassland

#### Tetragnatha pinicola

L. Koch, 1870

##### Materials

- **Type status:** Other material. **Occurrence:** recordedBy: Kuntner, Gregorič, Čandek; sex: 1 male; **Location:** locationID: CH09; country: Switzerland; locality: Pennine Alps, Mattertal; minimumElevationInMeters: 1447; maximumElevationInMeters: 1447; decimalLatitude: 46.0976; decimalLongitude: 7.7789; **Event:** eventDate: 2011-07-08; habitat: forest and meadow near river
- **Type status:** Other material. **Occurrence:** recordedBy: Čandek; sex: 1 male; **Location:** locationID: SI61; country: Slovenia; locality: Sekirišče; minimumElevationInMeters: 750; maximumElevationInMeters: 750; decimalLatitude: 45.8631; decimalLongitude: 14.5367; **Event:** eventDate: 2011-06-23/2012-06-21; habitat: house, grassland, overgrowth

#### Theridiidae

Sundevall, 1833

#### Asagena phalerata

(Panzer, 1801)

##### Materials

- **Type status:** Other material. **Occurrence:** recordedBy: Čandek; sex: 1 female; **Location:** locationID: SI46; country: Slovenia; locality: Šešče pri Preboldu; minimumElevationInMeters: 284; maximumElevationInMeters: 285; decimalLatitude: 46.2356; decimalLongitude: 15.1228; **Event:** eventDate: 2011-06-13/2012-06-22; habitat: house and surroundings

#### Crustulina guttata

(Wider, 1834)

##### Materials

- **Type status:** Other material. **Occurrence:** recordedBy: Gregorič, Čandek, Kralj-Fišer; sex: 1 male; **Location:** locationID: SI56; country: Slovenia; locality: Dinaric Karst, Novelo; minimumElevationInMeters: 358; maximumElevationInMeters: 359; decimalLatitude: 45.8533; decimalLongitude: 13.6552; **Event:** eventDate: 2011-04-04/05-10; habitat: overgrowth
- **Type status:** Other material. **Occurrence:** recordedBy: Gregorič, Čandek; sex: 1 male; **Location:** locationID: SI57; country: Slovenia; locality: Dinaric Karst, Novelo; minimumElevationInMeters: 325; maximumElevationInMeters: 325; decimalLatitude: 45.8482; decimalLongitude: 13.6584; **Event:** eventDate: 2011-05-10; habitat: grassland

#### Crustulina scabripes

Simon, 1881

##### Materials

- **Type status:** Other material. **Occurrence:** recordedBy: Kuntner, Gregorič, Čandek, Kralj-Fišer, Cheng; sex: 2 males; **Location:** locationID: SI41; country: Slovenia; locality: Socerb, Osp; minimumElevationInMeters: 116; maximumElevationInMeters: 116; decimalLatitude: 45.5819; decimalLongitude: 13.8558; **Event:** eventDate: 2012-06-07; habitat: trail from Socerb to Osp

#### Dipoena melanogaster

(C. L. Koch, 1837)

##### Materials

- **Type status:** Other material. **Occurrence:** recordedBy: Kuntner, Gregorič, Čandek, Kralj-Fišer, Cheng; sex: 1 female; **Location:** locationID: SI41; country: Slovenia; locality: Socerb, Osp; minimumElevationInMeters: 116; maximumElevationInMeters: 116; decimalLatitude: 45.5819; decimalLongitude: 13.8558; **Event:** eventDate: 2012-06-07; habitat: trail from Socerb to Osp

#### Enoplognatha afrodite

Hippa & Oksala, 1983

##### Materials

- **Type status:** Other material. **Occurrence:** recordedBy: Kuntner, Gregorič, Čandek, Kralj-Fišer, Cheng; sex: 2 females; **Location:** locationID: SI41; country: Slovenia; locality: Socerb, Osp; minimumElevationInMeters: 116; maximumElevationInMeters: 116; decimalLatitude: 45.5819; decimalLongitude: 13.8558; **Event:** eventDate: 2012-06-07; habitat: trail from Socerb to Osp

#### Enoplognatha latimana

Hippa & Oksala, 1982

##### Materials

- **Type status:** Other material. **Occurrence:** recordedBy: Kuntner, Gregorič, Čandek; sex: 1 male; **Location:** locationID: CH09; country: Switzerland; locality: Pennine Alps, Mattertal; minimumElevationInMeters: 1447; maximumElevationInMeters: 1447; decimalLatitude: 46.0976; decimalLongitude: 7.7789; **Event:** eventDate: 2011-07-08; habitat: forest and meadow near river
- **Type status:** Other material. **Occurrence:** recordedBy: Kostanjšek, RTŠB 2012; sex: 1 male; **Location:** locationID: SI72; country: Slovenia; locality: Dolnja Košana; minimumElevationInMeters: 415; maximumElevationInMeters: 415; decimalLatitude: 45.6579; decimalLongitude: 14.1384; **Event:** eventDate: 2012-07-21; habitat: rastlinje ob potoku

#### Enoplognatha ovata

(Clerck, 1757)

##### Materials

- **Type status:** Other material. **Occurrence:** recordedBy: Kuntner, Gregorič, Čandek; sex: 5 females, 3 males; **Location:** locationID: CH09; country: Switzerland; locality: Pennine Alps, Mattertal; minimumElevationInMeters: 1447; maximumElevationInMeters: 1447; decimalLatitude: 46.0976; decimalLongitude: 7.7789; **Event:** eventDate: 2011-07-08; habitat: forest and meadow near river
- **Type status:** Other material. **Occurrence:** recordedBy: Kuntner, Gregorič, Čandek; sex: 4 females, 1 male; **Location:** locationID: CH11; country: Switzerland; locality: Bernese Alps, Lake Brienz; minimumElevationInMeters: 600; maximumElevationInMeters: 600; decimalLatitude: 46.7569; decimalLongitude: 8.0107; **Event:** eventDate: 2011-07-10; habitat: meadows and forest
- **Type status:** Other material. **Occurrence:** recordedBy: Kuntner, Gregorič, Čandek; sex: 3 females, 3 males; **Location:** locationID: CH12; country: Switzerland; locality: Bernese Alps, Nessental; minimumElevationInMeters: 930; maximumElevationInMeters: 930; decimalLatitude: 46.7213; decimalLongitude: 8.3039; **Event:** eventDate: 2011-07-10; habitat: grassland and loan trees
- **Type status:** Other material. **Occurrence:** recordedBy: Kuntner, Gregorič, Čandek; sex: 1 female, 1 male; **Location:** locationID: CH15; country: Switzerland; locality: Glarus Alps, near Affeier; minimumElevationInMeters: 817; maximumElevationInMeters: 817; decimalLatitude: 46.7606; decimalLongitude: 9.0933; **Event:** eventDate: 2011-07-10; habitat: meadow and forest
- **Type status:** Other material. **Occurrence:** recordedBy: Kostanjšek, RTŠB 2011; sex: 1 female; **Location:** locationID: SI04; country: Slovenia; locality: Cerkvenjak; minimumElevationInMeters: 230; maximumElevationInMeters: 230; decimalLatitude: 46.5641; decimalLongitude: 15.9863; **Event:** eventDate: 2011-07-22; habitat: grassland
- **Type status:** Other material. **Occurrence:** recordedBy: Kuntner, Gregorič, Lokovšek; sex: 1 female; **Location:** locationID: SI39; country: Slovenia; locality: Primostek; minimumElevationInMeters: 157; maximumElevationInMeters: 157; decimalLatitude: 45.6299; decimalLongitude: 15.2997; **Event:** eventDate: 2010-08-24; habitat: grassland
- **Type status:** Other material. **Occurrence:** recordedBy: Čandek; sex: 2 females, 1 male; **Location:** locationID: SI61; country: Slovenia; locality: Sekirišče; minimumElevationInMeters: 750; maximumElevationInMeters: 750; decimalLatitude: 45.8631; decimalLongitude: 14.5367; **Event:** eventDate: 2011-06-23/2012-06-21; habitat: house, grassland, overgrowth

#### Episinus angulatus

(Blackwall, 1836)

##### Materials

- **Type status:** Other material. **Occurrence:** recordedBy: Kostanjšek, RTŠB 2011; sex: 1 male; **Location:** locationID: SI18; country: Slovenia; locality: Podgorje; minimumElevationInMeters: 220; maximumElevationInMeters: 220; decimalLatitude: 46.7183; decimalLongitude: 15.8243; **Event:** eventDate: 2011-07-26; habitat: vegetation at trail

#### Episinus maculipes

Cavanna, 1876

##### Materials

- **Type status:** Other material. **Occurrence:** recordedBy: Kuntner, Gregorič, Čandek, Kralj-Fišer, Cheng; sex: 1 female; **Location:** locationID: SI41; country: Slovenia; locality: Socerb, Osp; minimumElevationInMeters: 116; maximumElevationInMeters: 116; decimalLatitude: 45.5819; decimalLongitude: 13.8558; **Event:** eventDate: 2012-06-07; habitat: trail from Socerb to Osp

#### Episinus truncatus

Latreille, 1809

##### Materials

- **Type status:** Other material. **Occurrence:** recordedBy: Kuntner, Gregorič, Čandek; sex: 2 females, 2 males; **Location:** locationID: CH11; country: Switzerland; locality: Bernese Alps, Lake Brienz; minimumElevationInMeters: 600; maximumElevationInMeters: 600; decimalLatitude: 46.7569; decimalLongitude: 8.0107; **Event:** eventDate: 2011-07-10; habitat: meadows and forest
- **Type status:** Other material. **Occurrence:** recordedBy: Kuntner, Gregorič, Čandek; sex: 3 females; **Location:** locationID: CH27; country: Switzerland; locality: Grison Alps, road to Davos; minimumElevationInMeters: 1180; maximumElevationInMeters: 1180; decimalLatitude: 46.6808; decimalLongitude: 9.6557; **Event:** eventDate: 2011-07-15; habitat: roadside vegetation and forest edge
- **Type status:** Other material. **Occurrence:** recordedBy: Kostanjšek, RTŠB 2011; sex: 1 female; **Location:** locationID: SI04; country: Slovenia; locality: Cerkvenjak; minimumElevationInMeters: 230; maximumElevationInMeters: 230; decimalLatitude: 46.5641; decimalLongitude: 15.9863; **Event:** eventDate: 2011-07-22; habitat: grassland
- **Type status:** Other material. **Occurrence:** recordedBy: Kuntner, Gregorič, Čandek, Kralj-Fišer, Cheng; sex: 1 female; **Location:** locationID: SI41; country: Slovenia; locality: Socerb, Osp; minimumElevationInMeters: 116; maximumElevationInMeters: 116; decimalLatitude: 45.5819; decimalLongitude: 13.8558; **Event:** eventDate: 2012-06-07; habitat: trail from Socerb to Osp

#### Euryopis flavomaculata

(C. L. Koch, 1836)

##### Materials

- **Type status:** Other material. **Occurrence:** recordedBy: Gregorič, Čandek; sex: 1 male; **Location:** locationID: SI54; country: Slovenia; locality: Dinaric Karst, Griže; minimumElevationInMeters: 484; maximumElevationInMeters: 484; decimalLatitude: 45.7510; decimalLongitude: 13.9504; **Event:** eventDate: 2011-05-10; habitat: forest

#### Heterotheridion nigrovariegatum

(Simon, 1873)

##### Materials

- **Type status:** Other material. **Occurrence:** recordedBy: Kuntner, Gregorič, Čandek, Kralj-Fišer, Cheng; sex: 2 females, 1 male; **Location:** locationID: SI41; country: Slovenia; locality: Socerb, Osp; minimumElevationInMeters: 116; maximumElevationInMeters: 116; decimalLatitude: 45.5819; decimalLongitude: 13.8558; **Event:** eventDate: 2012-06-07; habitat: trail from Socerb to Osp
- **Type status:** Other material. **Occurrence:** recordedBy: Kuntner, Čandek; sex: 1 female; **Location:** locationID: SI50; country: Slovenia; locality: Sp. Prapreče; minimumElevationInMeters: 351; maximumElevationInMeters: 351; decimalLatitude: 46.1620; decimalLongitude: 14.6933; **Event:** eventDate: 2010-08-03/2012-05-28; habitat: house and surroundings

#### Neottiura bimaculata

(Linnaeus, 1767)

##### Materials

- **Type status:** Other material. **Occurrence:** recordedBy: Kuntner, Gregorič, Čandek; sex: 3 females, 1 male; **Location:** locationID: CH09; country: Switzerland; locality: Pennine Alps, Mattertal; minimumElevationInMeters: 1447; maximumElevationInMeters: 1447; decimalLatitude: 46.0976; decimalLongitude: 7.7789; **Event:** eventDate: 2011-07-08; habitat: forest and meadow near river
- **Type status:** Other material. **Occurrence:** recordedBy: Gregorič, Čandek; sex: 1 male; **Location:** locationID: SI53; country: Slovenia; locality: Dinaric Karst, Griže; minimumElevationInMeters: 434; maximumElevationInMeters: 434; decimalLatitude: 45.7548; decimalLongitude: 13.9495; **Event:** eventDate: 2011-05-10/2011-06-21; habitat: grassland
- **Type status:** Other material. **Occurrence:** recordedBy: Čandek; sex: 5 females, 2 males; **Location:** locationID: SI61; country: Slovenia; locality: Sekirišče; minimumElevationInMeters: 750; maximumElevationInMeters: 750; decimalLatitude: 45.8631; decimalLongitude: 14.5367; **Event:** eventDate: 2011-06-23/2012-06-21; habitat: house, grassland, overgrowth

#### Neottiura herbigrada

(Simon, 1873)

##### Materials

- **Type status:** Other material. **Occurrence:** recordedBy: Gregorič, Čandek, Kralj-Fišer; sex: 1 male; **Location:** locationID: SI55; country: Slovenia; locality: Dinaric Karst, Lokvice; minimumElevationInMeters: 273; maximumElevationInMeters: 275; decimalLatitude: 45.8659; decimalLongitude: 13.6102; **Event:** eventDate: 2011-04-04/05-10; habitat: overgrowth

#### Neottiura suaveolens

(Simon, 1879)

##### Materials

- **Type status:** Other material. **Occurrence:** recordedBy: Kostanjšek, RTŠB 2012; sex: 2 females; **Location:** locationID: SI26; country: Slovenia; locality: Dolnja Košana; minimumElevationInMeters: 420; maximumElevationInMeters: 420; decimalLatitude: 45.6587; decimalLongitude: 14.1397; **Event:** eventDate: 2012-07-21; habitat: grassland
- **Type status:** Other material. **Occurrence:** recordedBy: Čandek; sex: 4 females; **Location:** locationID: SI61; country: Slovenia; locality: Sekirišče; minimumElevationInMeters: 750; maximumElevationInMeters: 750; decimalLatitude: 45.8631; decimalLongitude: 14.5367; **Event:** eventDate: 2011-06-23/2012-06-21; habitat: house, grassland, overgrowth

#### Paidiscura pallens

(Blackwall, 1834)

##### Materials

- **Type status:** Other material. **Occurrence:** recordedBy: Čandek; sex: 2 females; **Location:** locationID: SI61; country: Slovenia; locality: Sekirišče; minimumElevationInMeters: 750; maximumElevationInMeters: 750; decimalLatitude: 45.8631; decimalLongitude: 14.5367; **Event:** eventDate: 2011-06-23/2012-06-21; habitat: house, grassland, overgrowth

#### Parasteatoda lunata

(Clerck, 1757)

##### Materials

- **Type status:** Other material. **Occurrence:** recordedBy: Kostanjšek, RTŠB 2011; sex: 1 female; **Location:** locationID: SI01; country: Slovenia; locality: Biš; minimumElevationInMeters: 225; maximumElevationInMeters: 225; decimalLatitude: 46.5374; decimalLongitude: 15.8963; **Event:** eventDate: 2011-07-22; habitat: forest
- **Type status:** Other material. **Occurrence:** recordedBy: Kuntner, Lokovšek; sex: 1 female; **Location:** locationID: SI40; country: Slovenia; locality: Slavnik; minimumElevationInMeters: 816; maximumElevationInMeters: 816; decimalLatitude: 45.5499; decimalLongitude: 13.9619; **Event:** eventDate: 2010-08-26; habitat: grassland and forest
- **Type status:** Other material. **Occurrence:** recordedBy: Kuntner, Gregorič, Čandek, Kralj-Fišer, Cheng; sex: 3 females; **Location:** locationID: SI41; country: Slovenia; locality: Socerb, Osp; minimumElevationInMeters: 116; maximumElevationInMeters: 116; decimalLatitude: 45.5819; decimalLongitude: 13.8558; **Event:** eventDate: 2012-06-07; habitat: trail from Socerb to Osp
- **Type status:** Other material. **Occurrence:** recordedBy: Čandek; sex: 1 female, 1 male; **Location:** locationID: SI59; country: Slovenia; locality: Budanje; minimumElevationInMeters: 305; maximumElevationInMeters: 305; decimalLatitude: 45.8797; decimalLongitude: 13.9468; **Event:** eventDate: 2011-05-07; habitat: forest

#### Parasteatoda tepidariorum

(C. L. Koch, 1841)

##### Materials

- **Type status:** Other material. **Occurrence:** recordedBy: Kostanjšek, RTŠB 2011; sex: 1 female; **Location:** locationID: SI10; country: Slovenia; locality: Sv. Jurij ob Ščavnici; minimumElevationInMeters: 195; maximumElevationInMeters: 195; decimalLatitude: 46.5573; decimalLongitude: 16.0386; **Event:** eventDate: 2011-07-25; habitat: wooden house
- **Type status:** Other material. **Occurrence:** recordedBy: Kuntner, Gregorič, Lokovšek; sex: 2 females; **Location:** locationID: SI39; country: Slovenia; locality: Primostek; minimumElevationInMeters: 157; maximumElevationInMeters: 157; decimalLatitude: 45.6299; decimalLongitude: 15.2997; **Event:** eventDate: 2010-08-24; habitat: grassland
- **Type status:** Other material. **Occurrence:** recordedBy: Čandek; sex: 1 female; **Location:** locationID: SI46; country: Slovenia; locality: Šešče pri Preboldu; minimumElevationInMeters: 284; maximumElevationInMeters: 285; decimalLatitude: 46.2356; decimalLongitude: 15.1228; **Event:** eventDate: 2011-06-13/2012-06-22; habitat: house and surroundings
- **Type status:** Other material. **Occurrence:** recordedBy: Kuntner, Čandek; sex: 1 female, 1 male; **Location:** locationID: SI50; country: Slovenia; locality: Sp. Prapreče; minimumElevationInMeters: 351; maximumElevationInMeters: 351; decimalLatitude: 46.1620; decimalLongitude: 14.6933; **Event:** eventDate: 2010-08-03/2012-05-28; habitat: house and surroundings
- **Type status:** Other material. **Occurrence:** recordedBy: Čandek; sex: 1 female; **Location:** locationID: SI61; country: Slovenia; locality: Sekirišče; minimumElevationInMeters: 750; maximumElevationInMeters: 750; decimalLatitude: 45.8631; decimalLongitude: 14.5367; **Event:** eventDate: 2011-06-23/2012-06-21; habitat: house, grassland, overgrowth

#### Phylloneta impressa

(L. Koch, 1881)

##### Materials

- **Type status:** Other material. **Occurrence:** recordedBy: Kuntner, Gregorič, Čandek; sex: 2 females; **Location:** locationID: CH01; country: Switzerland; locality: Bernese Alps, Gasteretal; minimumElevationInMeters: 1662; maximumElevationInMeters: 1662; decimalLatitude: 46.4457; decimalLongitude: 7.7413; **Event:** eventDate: 2011-07-07; habitat: alpine meadow
- **Type status:** Other material. **Occurrence:** recordedBy: Kuntner, Gregorič, Čandek; sex: 1 female; **Location:** locationID: CH12; country: Switzerland; locality: Bernese Alps, Nessental; minimumElevationInMeters: 930; maximumElevationInMeters: 930; decimalLatitude: 46.7213; decimalLongitude: 8.3039; **Event:** eventDate: 2011-07-10; habitat: grassland and loan trees
- **Type status:** Other material. **Occurrence:** recordedBy: Kuntner, Gregorič, Čandek; sex: 1 female; **Location:** locationID: CH15; country: Switzerland; locality: Glarus Alps, near Affeier; minimumElevationInMeters: 817; maximumElevationInMeters: 817; decimalLatitude: 46.7606; decimalLongitude: 9.0933; **Event:** eventDate: 2011-07-10; habitat: meadow and forest
- **Type status:** Other material. **Occurrence:** recordedBy: Kuntner, Gregorič, Čandek; sex: 3 males; **Location:** locationID: CH17; country: Switzerland; locality: Engadin, Bivio; minimumElevationInMeters: 1780; maximumElevationInMeters: 1780; decimalLatitude: 46.4753; decimalLongitude: 9.6469; **Event:** eventDate: 2011-07-11; habitat: forest and river edge
- **Type status:** Other material. **Occurrence:** recordedBy: Kuntner, Gregorič, Čandek; sex: 2 males; **Location:** locationID: CH19; country: Switzerland; locality: Grison Alps, Alp Flix, Salategnas; minimumElevationInMeters: 1910; maximumElevationInMeters: 1910; decimalLatitude: 46.5172; decimalLongitude: 9.6533; **Event:** eventDate: 2011-07-12; habitat: flat uncut grassland
- **Type status:** Other material. **Occurrence:** recordedBy: Kuntner, Gregorič, Čandek; sex: 9 females, 1 male; **Location:** locationID: CH20; country: Switzerland; locality: Grison Alps, Alp Flix, Salategnas; minimumElevationInMeters: 1900; maximumElevationInMeters: 1900; decimalLatitude: 46.5181; decimalLongitude: 9.6480; **Event:** eventDate: 2011-07-12; habitat: grazed meadow
- **Type status:** Other material. **Occurrence:** recordedBy: Kuntner, Gregorič, Čandek; sex: 8 females; **Location:** locationID: CH23; country: Switzerland; locality: Grison Alps, Alp Flix, Salategnas; minimumElevationInMeters: 1900; maximumElevationInMeters: 1900; decimalLatitude: 46.5141; decimalLongitude: 9.6448; **Event:** eventDate: 2011-07-12; habitat: forest opening, grass and shrubs
- **Type status:** Other material. **Occurrence:** recordedBy: Kuntner, Gregorič, Čandek; sex: 1 female; **Location:** locationID: CH30; country: Switzerland; locality: Grison Alps, Alp Flix - Lai Flix; minimumElevationInMeters: 1967; maximumElevationInMeters: 1967; decimalLatitude: 46.5358; decimalLongitude: 9.6409; **Event:** eventDate: 2011-07-16; habitat: next to alpine lake

#### Phylloneta sisyphia

(Clerck, 1757)

##### Materials

- **Type status:** Other material. **Occurrence:** recordedBy: Kuntner, Gregorič, Čandek; sex: 2 females; **Location:** locationID: CH03; country: Switzerland; locality: Bernese Alps, Gasteretal; minimumElevationInMeters: 1520; maximumElevationInMeters: 1520; decimalLatitude: 46.4498; decimalLongitude: 7.7135; **Event:** eventDate: 2011-07-07; habitat: spruce forest
- **Type status:** Other material. **Occurrence:** recordedBy: Kuntner, Gregorič, Čandek; sex: 1 female; **Location:** locationID: CH05; country: Switzerland; locality: Bernese Alps, Gasteretal; minimumElevationInMeters: 1380; maximumElevationInMeters: 1380; decimalLatitude: 46.4674; decimalLongitude: 7.6640; **Event:** eventDate: 2011-07-07; habitat: river vegetation
- **Type status:** Other material. **Occurrence:** recordedBy: Kuntner, Gregorič, Čandek; sex: 1 female; **Location:** locationID: CH09; country: Switzerland; locality: Pennine Alps, Mattertal; minimumElevationInMeters: 1447; maximumElevationInMeters: 1447; decimalLatitude: 46.0976; decimalLongitude: 7.7789; **Event:** eventDate: 2011-07-08; habitat: forest and meadow near river
- **Type status:** Other material. **Occurrence:** recordedBy: Kuntner, Gregorič, Čandek; sex: 1 female, 1 male; **Location:** locationID: CH17; country: Switzerland; locality: Engadin, Bivio; minimumElevationInMeters: 1780; maximumElevationInMeters: 1780; decimalLatitude: 46.4753; decimalLongitude: 9.6469; **Event:** eventDate: 2011-07-11; habitat: forest and river edge
- **Type status:** Other material. **Occurrence:** recordedBy: Kuntner, Gregorič, Čandek; sex: 1 male; **Location:** locationID: CH18; country: Switzerland; locality: Grison Alps, Alp Flix, Salategnas; minimumElevationInMeters: 1900; maximumElevationInMeters: 1900; decimalLatitude: 46.5166; decimalLongitude: 9.6523; **Event:** eventDate: 2011-07-12/19; habitat: around house
- **Type status:** Other material. **Occurrence:** recordedBy: Kuntner, Gregorič, Čandek; sex: 1 female; **Location:** locationID: CH23; country: Switzerland; locality: Grison Alps, Alp Flix, Salategnas; minimumElevationInMeters: 1900; maximumElevationInMeters: 1900; decimalLatitude: 46.5141; decimalLongitude: 9.6448; **Event:** eventDate: 2011-07-12; habitat: forest opening, grass and shrubs
- **Type status:** Other material. **Occurrence:** recordedBy: Kuntner, Gregorič, Čandek; sex: 3 females; **Location:** locationID: CH27; country: Switzerland; locality: Grison Alps, road to Davos; minimumElevationInMeters: 1180; maximumElevationInMeters: 1180; decimalLatitude: 46.6808; decimalLongitude: 9.6557; **Event:** eventDate: 2011-07-15; habitat: roadside vegetation and forest edge

#### Platnickina tincta

(Walckenaer, 1802)

##### Materials

- **Type status:** Other material. **Occurrence:** recordedBy: Kostanjšek, RTŠB 2011; sex: 1 female; **Location:** locationID: SI15; country: Slovenia; locality: Apače; minimumElevationInMeters: 220; maximumElevationInMeters: 220; decimalLatitude: 46.6804; decimalLongitude: 15.8988; **Event:** eventDate: 2011-07-26; habitat: forest

#### Robertus lividus

(Blackwall, 1836)

##### Materials

- **Type status:** Other material. **Occurrence:** recordedBy: Kuntner, Gregorič, Čandek; sex: 1 male; **Location:** locationID: CH09; country: Switzerland; locality: Pennine Alps, Mattertal; minimumElevationInMeters: 1447; maximumElevationInMeters: 1447; decimalLatitude: 46.0976; decimalLongitude: 7.7789; **Event:** eventDate: 2011-07-08; habitat: forest and meadow near river
- **Type status:** Other material. **Occurrence:** recordedBy: Kuntner, Gregorič, Čandek; sex: 1 female; **Location:** locationID: CH24; country: Switzerland; locality: Grison Alps, Alp Flix, Salategnas; minimumElevationInMeters: 1830; maximumElevationInMeters: 1830; decimalLatitude: 46.5131; decimalLongitude: 9.6430; **Event:** eventDate: 2011-07-12; habitat: meadow and forest

#### Robertus mediterraneus

Eskov, 1987

##### Materials

- **Type status:** Other material. **Occurrence:** recordedBy: Kuntner, Gregorič, Čandek; sex: 1 male; **Location:** locationID: CH15; country: Switzerland; locality: Glarus Alps, near Affeier; minimumElevationInMeters: 817; maximumElevationInMeters: 817; decimalLatitude: 46.7606; decimalLongitude: 9.0933; **Event:** eventDate: 2011-07-10; habitat: meadow and forest

#### Robertus scoticus

Jackson, 1914

##### Materials

- **Type status:** Other material. **Occurrence:** recordedBy: Kostanjšek, RTŠB 2012; sex: 1 female; **Location:** locationID: SI21; country: Slovenia; locality: Jurišče; minimumElevationInMeters: 730; maximumElevationInMeters: 730; decimalLatitude: 45.6735; decimalLongitude: 14.3093; **Event:** eventDate: 2012-07-23; habitat: overgrown grassland

#### Robertus truncorum

(L. Koch, 1872)

##### Materials

- **Type status:** Other material. **Occurrence:** recordedBy: Kuntner, Gregorič, Čandek; sex: 1 female; **Location:** locationID: CH26; country: Switzerland; locality: Grison Alps, Alp Flix, Salategnas; minimumElevationInMeters: 1987; maximumElevationInMeters: 1987; decimalLatitude: 46.5166; decimalLongitude: 9.6516; **Event:** eventDate: 2011-07-14; habitat: grassland
- **Type status:** Other material. **Occurrence:** recordedBy: Kuntner, Gregorič, Čandek; sex: 1 male; **Location:** locationID: CH31; country: Switzerland; locality: Grison Alps, Alp Flix - Lai Neir; minimumElevationInMeters: 1910; maximumElevationInMeters: 1910; decimalLatitude: 46.5343; decimalLongitude: 9.6375; **Event:** eventDate: 2011-07-16; habitat: lake and swamp around forest

#### Sardinidion blackwalli

(O. P.-Cambridge, 1871)

##### Materials

- **Type status:** Other material. **Occurrence:** recordedBy: Čandek; sex: 1 female; **Location:** locationID: SI28; country: Slovenia; locality: Bistra; minimumElevationInMeters: 266; maximumElevationInMeters: 266; decimalLatitude: 45.9475; decimalLongitude: 14.3369; **Event:** eventDate: 2011-05-17; habitat: grassland
- **Type status:** Other material. **Occurrence:** recordedBy: Kuntner; sex: 1 male; **Location:** locationID: SI73; country: Slovenia; locality: Ljubljana, Galjevica; minimumElevationInMeters: 290; maximumElevationInMeters: 290; decimalLatitude: 46.0352; decimalLongitude: 14.5211; **Event:** eventDate: 2011-05-31; habitat: house

#### Simitidion simile

(C. L. Koch, 1836)

##### Materials

- **Type status:** Other material. **Occurrence:** recordedBy: Kuntner, Gregorič, Čandek, Kralj-Fišer, Cheng; sex: 1 male; **Location:** locationID: SI41; country: Slovenia; locality: Socerb, Osp; minimumElevationInMeters: 116; maximumElevationInMeters: 116; decimalLatitude: 45.5819; decimalLongitude: 13.8558; **Event:** eventDate: 2012-06-07; habitat: trail from Socerb to Osp
- **Type status:** Other material. **Occurrence:** recordedBy: Gregorič, Čandek, Kralj-Fišer; sex: 2 males; **Location:** locationID: SI52; country: Slovenia; locality: Dinaric Karst, Griže; minimumElevationInMeters: 484; maximumElevationInMeters: 484; decimalLatitude: 45.7506; decimalLongitude: 13.9509; **Event:** eventDate: 2011-04-04/05-10; habitat: overgrowth

#### Steatoda bipunctata

(Linnaeus, 1758)

##### Materials

- **Type status:** Other material. **Occurrence:** recordedBy: Kuntner, Gregorič, Čandek; sex: 1 female; **Location:** locationID: CH18; country: Switzerland; locality: Grison Alps, Alp Flix, Salategnas; minimumElevationInMeters: 1900; maximumElevationInMeters: 1900; decimalLatitude: 46.5166; decimalLongitude: 9.6523; **Event:** eventDate: 2011-07-12/19; habitat: around house
- **Type status:** Other material. **Occurrence:** recordedBy: Kostanjšek, RTŠB 2011; sex: 1 female; **Location:** locationID: SI10; country: Slovenia; locality: Sv. Jurij ob Ščavnici; minimumElevationInMeters: 195; maximumElevationInMeters: 195; decimalLatitude: 46.5573; decimalLongitude: 16.0386; **Event:** eventDate: 2011-07-25; habitat: wooden house
- **Type status:** Other material. **Occurrence:** recordedBy: Kuntner, Gregorič, Lokovšek; sex: 1 male; **Location:** locationID: SI39; country: Slovenia; locality: Primostek; minimumElevationInMeters: 157; maximumElevationInMeters: 157; decimalLatitude: 45.6299; decimalLongitude: 15.2997; **Event:** eventDate: 2010-08-24; habitat: grassland
- **Type status:** Other material. **Occurrence:** recordedBy: Čandek; sex: 1 female; **Location:** locationID: SI46; country: Slovenia; locality: Šešče pri Preboldu; minimumElevationInMeters: 284; maximumElevationInMeters: 285; decimalLatitude: 46.2356; decimalLongitude: 15.1228; **Event:** eventDate: 2011-06-13/2012-06-22; habitat: house and surroundings
- **Type status:** Other material. **Occurrence:** recordedBy: Čandek; sex: 2 females; **Location:** locationID: SI65; country: Slovenia; locality: Dramlje; minimumElevationInMeters: 409; maximumElevationInMeters: 409; decimalLatitude: 46.2799; decimalLongitude: 15.4044; **Event:** eventDate: 2011-08-27; habitat: house and surroundings

#### Steatoda triangulosa

(Walckenaer, 1802)

##### Materials

- **Type status:** Other material. **Occurrence:** recordedBy: Rozman; sex: 1 male; **Location:** locationID: SI32; country: Slovenia; locality: Ljubljana; minimumElevationInMeters: 311; maximumElevationInMeters: 311; decimalLatitude: 46.0804; decimalLongitude: 14.4690; **Event:** eventDate: 2010-07-31; habitat: house
- **Type status:** Other material. **Occurrence:** recordedBy: Kuntner, Čandek; sex: 2 females, 1 male; **Location:** locationID: SI50; country: Slovenia; locality: Sp. Prapreče; minimumElevationInMeters: 351; maximumElevationInMeters: 351; decimalLatitude: 46.1620; decimalLongitude: 14.6933; **Event:** eventDate: 2010-08-03/2012-05-28; habitat: house and surroundings
- **Type status:** Other material. **Occurrence:** recordedBy: Čandek; sex: 3 males; **Location:** locationID: SI51; country: Slovenia; locality: Ljubljana, Nove Jarše; minimumElevationInMeters: 294; maximumElevationInMeters: 294; decimalLatitude: 46.0712; decimalLongitude: 14.5403; **Event:** eventDate: 2011-06-10/08-31; habitat: house
- **Type status:** Other material. **Occurrence:** recordedBy: Gregorič; sex: 1 male; **Location:** locationID: SI74; country: Slovenia; locality: Brezovica pri Ljubljani; minimumElevationInMeters: 297; maximumElevationInMeters: 297; decimalLatitude: 46.0154; decimalLongitude: 14.4119; **Event:** eventDate: 2010-08-17; habitat: house

#### Theridion betteni

Wiehle, 1960

##### Materials

- **Type status:** Other material. **Occurrence:** recordedBy: Kuntner, Gregorič, Čandek; sex: 1 female; **Location:** locationID: CH01; country: Switzerland; locality: Bernese Alps, Gasteretal; minimumElevationInMeters: 1662; maximumElevationInMeters: 1662; decimalLatitude: 46.4457; decimalLongitude: 7.7413; **Event:** eventDate: 2011-07-07; habitat: alpine meadow

#### Theridion pinastri

L. Koch, 1872

##### Materials

- **Type status:** Other material. **Occurrence:** recordedBy: Kuntner, Gregorič, Čandek, Kralj-Fišer, Cheng; sex: 1 female, 1 male; **Location:** locationID: SI41; country: Slovenia; locality: Socerb, Osp; minimumElevationInMeters: 116; maximumElevationInMeters: 116; decimalLatitude: 45.5819; decimalLongitude: 13.8558; **Event:** eventDate: 2012-06-07; habitat: trail from Socerb to Osp
- **Type status:** Other material. **Occurrence:** recordedBy: Čandek; sex: 1 male; **Location:** locationID: SI61; country: Slovenia; locality: Sekirišče; minimumElevationInMeters: 750; maximumElevationInMeters: 750; decimalLatitude: 45.8631; decimalLongitude: 14.5367; **Event:** eventDate: 2011-06-23/2012-06-21; habitat: house, grassland, overgrowth

#### Theridion varians

Hahn, 1833

##### Materials

- **Type status:** Other material. **Occurrence:** recordedBy: Kuntner, Gregorič, Lokovšek; sex: 1 female; **Location:** locationID: SI39; country: Slovenia; locality: Primostek; minimumElevationInMeters: 157; maximumElevationInMeters: 157; decimalLatitude: 45.6299; decimalLongitude: 15.2997; **Event:** eventDate: 2010-08-24; habitat: grassland
- **Type status:** Other material. **Occurrence:** recordedBy: Gregorič, Kuntner, Čandek; sex: 1 male; **Location:** locationID: SI48; country: Slovenia; locality: Ljubljana, center; minimumElevationInMeters: 291; maximumElevationInMeters: 291; decimalLatitude: 46.0434; decimalLongitude: 14.5041; **Event:** eventDate: 2011-05-24/2012-06-19; habitat: house
- **Type status:** Other material. **Occurrence:** recordedBy: Čandek; sex: 1 male; **Location:** locationID: SI61; country: Slovenia; locality: Sekirišče; minimumElevationInMeters: 750; maximumElevationInMeters: 750; decimalLatitude: 45.8631; decimalLongitude: 14.5367; **Event:** eventDate: 2011-06-23/2012-06-21; habitat: house, grassland, overgrowth

#### Thomisidae

Sundevall, 1833

#### Diaea livens

Simon, 1876

##### Materials

- **Type status:** Other material. **Occurrence:** recordedBy: Čandek; sex: 1 male; **Location:** locationID: SI43; country: Slovenia; locality: Vipava; minimumElevationInMeters: 114; maximumElevationInMeters: 114; decimalLatitude: 45.8282; decimalLongitude: 13.9594; **Event:** eventDate: 2011-05-08; habitat: grassland

#### Ebrechtella tricuspidata

(Fabricius, 1775)

##### Materials

- **Type status:** Other material. **Occurrence:** recordedBy: Čandek; sex: 1 male; **Location:** locationID: SI28; country: Slovenia; locality: Bistra; minimumElevationInMeters: 266; maximumElevationInMeters: 266; decimalLatitude: 45.9475; decimalLongitude: 14.3369; **Event:** eventDate: 2011-05-17; habitat: grassland
- **Type status:** Other material. **Occurrence:** recordedBy: Kuntner, Gregorič, Lokovšek; sex: 3 males; **Location:** locationID: SI39; country: Slovenia; locality: Primostek; minimumElevationInMeters: 157; maximumElevationInMeters: 157; decimalLatitude: 45.6299; decimalLongitude: 15.2997; **Event:** eventDate: 2010-08-24; habitat: grassland

#### Heriaeus hirtus

(Latreille, 1819)

##### Materials

- **Type status:** Other material. **Occurrence:** recordedBy: Gregorič, Čandek, Kralj-Fišer; sex: 1 male; **Location:** locationID: SI55; country: Slovenia; locality: Dinaric Karst, Lokvice; minimumElevationInMeters: 273; maximumElevationInMeters: 275; decimalLatitude: 45.8659; decimalLongitude: 13.6102; **Event:** eventDate: 2011-04-04/05-10; habitat: overgrowth

#### Misumena vatia

(Clerck, 1757)

##### Materials

- **Type status:** Other material. **Occurrence:** recordedBy: Kostanjšek, RTŠB 2011; sex: 1 male; **Location:** locationID: SI19; country: Slovenia; locality: Ptujska cesta; minimumElevationInMeters: 240; maximumElevationInMeters: 240; decimalLatitude: 46.6283; decimalLongitude: 15.9973; **Event:** eventDate: 2011-07-26; habitat: grassland
- **Type status:** Other material. **Occurrence:** recordedBy: Čandek; sex: 1 female, 1 male; **Location:** locationID: SI38; country: Slovenia; locality: Poreče; minimumElevationInMeters: 135; maximumElevationInMeters: 135; decimalLatitude: 45.8188; decimalLongitude: 13.9692; **Event:** eventDate: 2011-05-08; habitat: grassland
- **Type status:** Other material. **Occurrence:** recordedBy: Čandek; sex: 1 female; **Location:** locationID: SI43; country: Slovenia; locality: Vipava; minimumElevationInMeters: 114; maximumElevationInMeters: 114; decimalLatitude: 45.8282; decimalLongitude: 13.9594; **Event:** eventDate: 2011-05-08; habitat: grassland
- **Type status:** Other material. **Occurrence:** recordedBy: Kuntner, Čandek; sex: 1 female; **Location:** locationID: SI50; country: Slovenia; locality: Sp. Prapreče; minimumElevationInMeters: 351; maximumElevationInMeters: 351; decimalLatitude: 46.1620; decimalLongitude: 14.6933; **Event:** eventDate: 2010-08-03/2012-05-28; habitat: house and surroundings
- **Type status:** Other material. **Occurrence:** recordedBy: Čandek; sex: 2 females, 1 male; **Location:** locationID: SI60; country: Slovenia; locality: Budanje; minimumElevationInMeters: 295; maximumElevationInMeters: 295; decimalLatitude: 45.8799; decimalLongitude: 13.9459; **Event:** eventDate: 2011-05-07; habitat: forest clearing
- **Type status:** Other material. **Occurrence:** recordedBy: Čandek; sex: 1 male; **Location:** locationID: SI61; country: Slovenia; locality: Sekirišče; minimumElevationInMeters: 750; maximumElevationInMeters: 750; decimalLatitude: 45.8631; decimalLongitude: 14.5367; **Event:** eventDate: 2011-06-23/2012-06-21; habitat: house, grassland, overgrowth

#### Ozyptila atomaria

(Panzer, 1801)

##### Materials

- **Type status:** Other material. **Occurrence:** recordedBy: Kuntner, Gregorič, Čandek; sex: 1 male; **Location:** locationID: CH25; country: Switzerland; locality: Grison Alps, Alp Flix, Salategnas; minimumElevationInMeters: 1950; maximumElevationInMeters: 1950; decimalLatitude: 46.5159; decimalLongitude: 9.6496; **Event:** eventDate: 2011-07-12/16; habitat: meadow and shrubs at stream

#### Ozyptila claveata

(Walckenaer, 1837)

##### Materials

- **Type status:** Other material. **Occurrence:** recordedBy: Gregorič, Čandek, Kralj-Fišer; sex: 1 male; **Location:** locationID: SI56; country: Slovenia; locality: Dinaric Karst, Novelo; minimumElevationInMeters: 358; maximumElevationInMeters: 359; decimalLatitude: 45.8533; decimalLongitude: 13.6552; **Event:** eventDate: 2011-04-04/05-10; habitat: overgrowth

#### Ozyptila trux

(Blackwall, 1846)

##### Materials

- **Type status:** Other material. **Occurrence:** recordedBy: Kuntner, Gregorič, Čandek; sex: 1 male; **Location:** locationID: CH05; country: Switzerland; locality: Bernese Alps, Gasteretal; minimumElevationInMeters: 1380; maximumElevationInMeters: 1380; decimalLatitude: 46.4674; decimalLongitude: 7.6640; **Event:** eventDate: 2011-07-07; habitat: river vegetation

#### Synema globosum

(Fabricius, 1775)

##### Materials

- **Type status:** Other material. **Occurrence:** recordedBy: Kuntner, Gregorič, Čandek; sex: 1 female; **Location:** locationID: CH15; country: Switzerland; locality: Glarus Alps, near Affeier; minimumElevationInMeters: 817; maximumElevationInMeters: 817; decimalLatitude: 46.7606; decimalLongitude: 9.0933; **Event:** eventDate: 2011-07-10; habitat: meadow and forest
- **Type status:** Other material. **Occurrence:** recordedBy: Čandek; sex: 2 males; **Location:** locationID: SI60; country: Slovenia; locality: Budanje; minimumElevationInMeters: 295; maximumElevationInMeters: 295; decimalLatitude: 45.8799; decimalLongitude: 13.9459; **Event:** eventDate: 2011-05-07; habitat: forest clearing

#### Thomisus onustus

Walckenaer, 1805

##### Materials

- **Type status:** Other material. **Occurrence:** recordedBy: Čandek; sex: 2 females, 4 males; **Location:** locationID: SI38; country: Slovenia; locality: Poreče; minimumElevationInMeters: 135; maximumElevationInMeters: 135; decimalLatitude: 45.8188; decimalLongitude: 13.9692; **Event:** eventDate: 2011-05-08; habitat: grassland
- **Type status:** Other material. **Occurrence:** recordedBy: Gregorič, Čandek; sex: 1 male; **Location:** locationID: SI57; country: Slovenia; locality: Dinaric Karst, Novelo; minimumElevationInMeters: 325; maximumElevationInMeters: 325; decimalLatitude: 45.8482; decimalLongitude: 13.6584; **Event:** eventDate: 2011-05-10; habitat: grassland

#### Tmarus piger

(Walckenaer, 1802)

##### Materials

- **Type status:** Other material. **Occurrence:** recordedBy: Čandek; sex: 1 female; **Location:** locationID: SI38; country: Slovenia; locality: Poreče; minimumElevationInMeters: 135; maximumElevationInMeters: 135; decimalLatitude: 45.8188; decimalLongitude: 13.9692; **Event:** eventDate: 2011-05-08; habitat: grassland
- **Type status:** Other material. **Occurrence:** recordedBy: Gregorič, Čandek, Kralj-Fišer; sex: 1 female; **Location:** locationID: SI55; country: Slovenia; locality: Dinaric Karst, Lokvice; minimumElevationInMeters: 273; maximumElevationInMeters: 275; decimalLatitude: 45.8659; decimalLongitude: 13.6102; **Event:** eventDate: 2011-04-04/05-10; habitat: overgrowth
- **Type status:** Other material. **Occurrence:** recordedBy: Čandek; sex: 1 female, 1 male; **Location:** locationID: SI58; country: Slovenia; locality: Budanje; minimumElevationInMeters: 243; maximumElevationInMeters: 243; decimalLatitude: 45.8743; decimalLongitude: 13.9497; **Event:** eventDate: 2011-05-07; habitat: school and surroundings
- **Type status:** Other material. **Occurrence:** recordedBy: Čandek; sex: 1 female; **Location:** locationID: SI59; country: Slovenia; locality: Budanje; minimumElevationInMeters: 305; maximumElevationInMeters: 305; decimalLatitude: 45.8797; decimalLongitude: 13.9468; **Event:** eventDate: 2011-05-07; habitat: forest

#### Xysticus acerbus

Thorell, 1872

##### Materials

- **Type status:** Other material. **Occurrence:** recordedBy: Čandek; sex: 1 female; **Location:** locationID: SI60; country: Slovenia; locality: Budanje; minimumElevationInMeters: 295; maximumElevationInMeters: 295; decimalLatitude: 45.8799; decimalLongitude: 13.9459; **Event:** eventDate: 2011-05-07; habitat: forest clearing

#### Xysticus audax

(Schrank, 1803)

##### Materials

- **Type status:** Other material. **Occurrence:** recordedBy: Čandek; sex: 1 female; **Location:** locationID: SI38; country: Slovenia; locality: Poreče; minimumElevationInMeters: 135; maximumElevationInMeters: 135; decimalLatitude: 45.8188; decimalLongitude: 13.9692; **Event:** eventDate: 2011-05-08; habitat: grassland
- **Type status:** Other material. **Occurrence:** recordedBy: Čandek; sex: 1 female; **Location:** locationID: SI43; country: Slovenia; locality: Vipava; minimumElevationInMeters: 114; maximumElevationInMeters: 114; decimalLatitude: 45.8282; decimalLongitude: 13.9594; **Event:** eventDate: 2011-05-08; habitat: grassland

#### Xysticus bifasciatus

C. L. Koch, 1837

##### Materials

- **Type status:** Other material. **Occurrence:** recordedBy: Čandek; sex: 1 male; **Location:** locationID: SI61; country: Slovenia; locality: Sekirišče; minimumElevationInMeters: 750; maximumElevationInMeters: 750; decimalLatitude: 45.8631; decimalLongitude: 14.5367; **Event:** eventDate: 2011-06-23/2012-06-21; habitat: house, grassland, overgrowth

#### Xysticus cristatus

(Clerck, 1757)

##### Materials

- **Type status:** Other material. **Occurrence:** recordedBy: Kuntner, Gregorič, Čandek; sex: 1 male; **Location:** locationID: CH19; country: Switzerland; locality: Grison Alps, Alp Flix, Salategnas; minimumElevationInMeters: 1910; maximumElevationInMeters: 1910; decimalLatitude: 46.5172; decimalLongitude: 9.6533; **Event:** eventDate: 2011-07-12; habitat: flat uncut grassland
- **Type status:** Other material. **Occurrence:** recordedBy: Čandek; sex: 1 male; **Location:** locationID: SI61; country: Slovenia; locality: Sekirišče; minimumElevationInMeters: 750; maximumElevationInMeters: 750; decimalLatitude: 45.8631; decimalLongitude: 14.5367; **Event:** eventDate: 2011-06-23/2012-06-21; habitat: house, grassland, overgrowth

#### Xysticus desidosus

Simon, 1875

##### Materials

- **Type status:** Other material. **Occurrence:** recordedBy: Čandek; sex: 1 female; **Location:** locationID: SI38; country: Slovenia; locality: Poreče; minimumElevationInMeters: 135; maximumElevationInMeters: 135; decimalLatitude: 45.8188; decimalLongitude: 13.9692; **Event:** eventDate: 2011-05-08; habitat: grassland

#### Xysticus erraticus

(Blackwall, 1834)

##### Materials

- **Type status:** Other material. **Occurrence:** recordedBy: Kostanjšek, RTŠB 2012; sex: 1 male; **Location:** locationID: SI25; country: Slovenia; locality: Dolnja Košana; minimumElevationInMeters: 435; maximumElevationInMeters: 435; decimalLatitude: 45.6646; decimalLongitude: 14.1350; **Event:** eventDate: 2012-07-27; habitat: grassland

#### Xysticus kempeleni

Thorell, 1872

##### Materials

- **Type status:** Other material. **Occurrence:** recordedBy: Čandek; sex: 1 female; **Location:** locationID: SI38; country: Slovenia; locality: Poreče; minimumElevationInMeters: 135; maximumElevationInMeters: 135; decimalLatitude: 45.8188; decimalLongitude: 13.9692; **Event:** eventDate: 2011-05-08; habitat: grassland
- **Type status:** Other material. **Occurrence:** recordedBy: Čandek; sex: 1 female; **Location:** locationID: SI60; country: Slovenia; locality: Budanje; minimumElevationInMeters: 295; maximumElevationInMeters: 295; decimalLatitude: 45.8799; decimalLongitude: 13.9459; **Event:** eventDate: 2011-05-07; habitat: forest clearing

#### Xysticus kochi

Thorell, 1872

##### Materials

- **Type status:** Other material. **Occurrence:** recordedBy: Čandek; sex: 1 male; **Location:** locationID: SI38; country: Slovenia; locality: Poreče; minimumElevationInMeters: 135; maximumElevationInMeters: 135; decimalLatitude: 45.8188; decimalLongitude: 13.9692; **Event:** eventDate: 2011-05-08; habitat: grassland
- **Type status:** Other material. **Occurrence:** recordedBy: Čandek; sex: 7 females, 1 male; **Location:** locationID: SI43; country: Slovenia; locality: Vipava; minimumElevationInMeters: 114; maximumElevationInMeters: 114; decimalLatitude: 45.8282; decimalLongitude: 13.9594; **Event:** eventDate: 2011-05-08; habitat: grassland
- **Type status:** Other material. **Occurrence:** recordedBy: Gregorič, Čandek; sex: 1 male; **Location:** locationID: SI53; country: Slovenia; locality: Dinaric Karst, Griže; minimumElevationInMeters: 434; maximumElevationInMeters: 434; decimalLatitude: 45.7548; decimalLongitude: 13.9495; **Event:** eventDate: 2011-05-10/2011-06-21; habitat: grassland
- **Type status:** Other material. **Occurrence:** recordedBy: Čandek; sex: 1 male; **Location:** locationID: SI60; country: Slovenia; locality: Budanje; minimumElevationInMeters: 295; maximumElevationInMeters: 295; decimalLatitude: 45.8799; decimalLongitude: 13.9459; **Event:** eventDate: 2011-05-07; habitat: forest clearing

#### Xysticus lanio

C. L. Koch, 1835

##### Materials

- **Type status:** Other material. **Occurrence:** recordedBy: Kuntner, Gregorič, Čandek, Kralj-Fišer, Cheng; sex: 1 female; **Location:** locationID: SI41; country: Slovenia; locality: Socerb, Osp; minimumElevationInMeters: 116; maximumElevationInMeters: 116; decimalLatitude: 45.5819; decimalLongitude: 13.8558; **Event:** eventDate: 2012-06-07; habitat: trail from Socerb to Osp

#### Xysticus lineatus

(Westring, 1851)

##### Materials

- **Type status:** Other material. **Occurrence:** recordedBy: Čandek; sex: 2 males; **Location:** locationID: SI61; country: Slovenia; locality: Sekirišče; minimumElevationInMeters: 750; maximumElevationInMeters: 750; decimalLatitude: 45.8631; decimalLongitude: 14.5367; **Event:** eventDate: 2011-06-23/2012-06-21; habitat: house, grassland, overgrowth

#### Xysticus macedonicus

Silhavy, 1944

##### Materials

- **Type status:** Other material. **Occurrence:** recordedBy: Kuntner, Gregorič, Čandek; sex: 1 male; **Location:** locationID: CH02; country: Switzerland; locality: Bernese Alps, Gasteretal; minimumElevationInMeters: 1698; maximumElevationInMeters: 1698; decimalLatitude: 46.4486; decimalLongitude: 7.7438; **Event:** eventDate: 2011-07-07; habitat: spruce thicket and grass

#### Xysticus tenebrosus

Silhavy, 1944

##### Materials

- **Type status:** Other material. **Occurrence:** recordedBy: Kuntner, Gregorič, Čandek, Kralj-Fišer, Cheng; sex: 3 males; **Location:** locationID: SI41; country: Slovenia; locality: Socerb, Osp; minimumElevationInMeters: 116; maximumElevationInMeters: 116; decimalLatitude: 45.5819; decimalLongitude: 13.8558; **Event:** eventDate: 2012-06-07; habitat: trail from Socerb to Osp

#### Titanoecidae

Lehtinen, 1967

#### Titanoeca tristis

L. Koch, 1872

##### Materials

- **Type status:** Other material. **Occurrence:** recordedBy: Kostanjšek, RTŠB 2012; sex: 1 female; **Location:** locationID: SI11; country: Slovenia; locality: Divača; minimumElevationInMeters: 460; maximumElevationInMeters: 460; decimalLatitude: 45.6835; decimalLongitude: 14.0166; **Event:** eventDate: 2012-07-22; habitat: grassland

#### Uloboridae

Thorell, 1869

#### Hyptiotes paradoxus

(C. L. Koch, 1834)

##### Materials

- **Type status:** Other material. **Occurrence:** recordedBy: Kostanjšek, RTŠB 2012; sex: 1 female; **Location:** locationID: SI08; country: Slovenia; locality: Ribnica, Pivka; minimumElevationInMeters: 400; maximumElevationInMeters: 400; decimalLatitude: 45.6333; decimalLongitude: 14.1392; **Event:** eventDate: 2012-07-21; habitat: forest
- **Type status:** Other material. **Occurrence:** recordedBy: Kuntner, Gregorič, Lokovšek; sex: 2 females; **Location:** locationID: SI39; country: Slovenia; locality: Primostek; minimumElevationInMeters: 157; maximumElevationInMeters: 157; decimalLatitude: 45.6299; decimalLongitude: 15.2997; **Event:** eventDate: 2010-08-24; habitat: grassland

#### Uloborus plumipes

Lucas, 1846

##### Materials

- **Type status:** Other material. **Occurrence:** recordedBy: Gregorič; sex: 4 females; **Location:** locationID: SI47; country: Slovenia; locality: Ljubljana, center; minimumElevationInMeters: 290; maximumElevationInMeters: 290; decimalLatitude: 46.0396; decimalLongitude: 14.5147; **Event:** eventDate: 2012-07-16; habitat: Botanical garden greenhouse

#### Uloborus walckenaerius

Latreille, 1806

##### Materials

- **Type status:** Other material. **Occurrence:** recordedBy: Gregorič, Čandek, Kralj-Fišer; **Location:** locationID: SI55; country: Slovenia; locality: Dinaric Karst, Lokvice; minimumElevationInMeters: 273; maximumElevationInMeters: 275; decimalLatitude: 45.8659; decimalLongitude: 13.6102; **Event:** eventDate: 2011-04-04/05-10; habitat: overgrowth

#### Zoridae

F. O. P.-Cambridge, 1893

#### Zora spinimana

(Sundevall, 1833)

##### Materials

- **Type status:** Other material. **Occurrence:** recordedBy: Kuntner, Gregorič, Čandek; sex: 2 females, 1 male; **Location:** locationID: CH02; country: Switzerland; locality: Bernese Alps, Gasteretal; minimumElevationInMeters: 1698; maximumElevationInMeters: 1698; decimalLatitude: 46.4486; decimalLongitude: 7.7438; **Event:** eventDate: 2011-07-07; habitat: spruce thicket and grass
- **Type status:** Other material. **Occurrence:** recordedBy: Kuntner, Gregorič, Čandek; sex: 1 female; **Location:** locationID: CH05; country: Switzerland; locality: Bernese Alps, Gasteretal; minimumElevationInMeters: 1380; maximumElevationInMeters: 1380; decimalLatitude: 46.4674; decimalLongitude: 7.6640; **Event:** eventDate: 2011-07-07; habitat: river vegetation
- **Type status:** Other material. **Occurrence:** recordedBy: Kuntner, Gregorič, Čandek; sex: 1 female; **Location:** locationID: CH24; country: Switzerland; locality: Grison Alps, Alp Flix, Salategnas; minimumElevationInMeters: 1830; maximumElevationInMeters: 1830; decimalLatitude: 46.5131; decimalLongitude: 9.6430; **Event:** eventDate: 2011-07-12; habitat: meadow and forest
- **Type status:** Other material. **Occurrence:** recordedBy: Kuntner, Gregorič, Čandek; sex: 1 female; **Location:** locationID: CH31; country: Switzerland; locality: Grison Alps, Alp Flix - Lai Neir; minimumElevationInMeters: 1910; maximumElevationInMeters: 1910; decimalLatitude: 46.5343; decimalLongitude: 9.6375; **Event:** eventDate: 2011-07-16; habitat: lake and swamp around forest
- **Type status:** Other material. **Occurrence:** recordedBy: Gregorič, Čandek, Kralj-Fišer; sex: 1 male; **Location:** locationID: SI56; country: Slovenia; locality: Dinaric Karst, Novelo; minimumElevationInMeters: 358; maximumElevationInMeters: 359; decimalLatitude: 45.8533; decimalLongitude: 13.6552; **Event:** eventDate: 2011-04-04/05-10; habitat: overgrowth
- **Type status:** Other material. **Occurrence:** recordedBy: Čandek; sex: 2 females; **Location:** locationID: SI61; country: Slovenia; locality: Sekirišče; minimumElevationInMeters: 750; maximumElevationInMeters: 750; decimalLatitude: 45.8631; decimalLongitude: 14.5367; **Event:** eventDate: 2011-06-23/2012-06-21; habitat: house, grassland, overgrowth

## Analysis

Altogether, we identified 1596 adult individuals, belonging to 324 species, 183 genera and 33 spider families. From the 76 localities in Slovenia, we recorded 227 species belonging to 144 genera and 31 families. From the 33 localities in Switzerland, we recorded 143 species belonging to 89 genera and 18 families. The number of unique species was 181 and 97 for Slovenia and Switzerland, respectively. As an indication of rare species in our survey, singletons (those species represented on the list with a single individual (Coddington et al. 1996, Coddington et al. 2009) represented 38,27% (124 species).

## Discussion

Faunistic research has recently been neglected in most scientific outlets. Yet, faunistic studies contain crucial base line data urgently needed for ecological work, and particularly for meaningful conservation decisions. We see no reason for taxonomic experts of particular clade, such as spiders, not to offer such hard produced data to the public, particularly if these researchers consulted an array of literature sources to back up the species identifications. Our survey spanning two central European countries, Slovenia and Switzerland, is larger in scope and in faunistic data compared with most temperate studies reported in the literature. A quick overview of the literature suggests that only one study of temperate spider fauna reports more than 300 species (Blick 2011), and an additional study reports on more than 275 species that we initially targeted in our study (Blick 1996, Blick 2009).

The main goals of our study was not to merely report on the species lists, nor to compare the lists between the two countries as the sampling was not structured qualitatively nor quantitatively. Rather, this report is the first step towards our larger efforts to process this freshly collected and expertly identified biological material further. Thus, our upcoming works will report on the inclusion of selected vouchers from this survey in permanent preservation facilities, both as tissue and as genome grade DNA. Furthermore, we intend to provide DNA barcode data for selected representatives of these reported faunas. Because the taxa reported here are expertly identified, upcoming DNA barcodes, once freely available on BOLD and GenBank databases, will facilitate species identification (Barrett and Hebert 2005).

## References

[B153993] Barrett Rowan D. H, Hebert Paul D. N (2005). Identifying spiders through DNA barcodes. Canadian Journal of Zoology.

[B154242] Blick T., Dorow WHO., Blick T., Kopelke JP. (1996). Naturwaldreservate in Hessen.

[B157325] Blick Theo, Dorow Wolfgang H. O., Blick Theo, Kopelke Jens-Peter (2009). Mitteilungen der Hessischen Landesforstverwaltung 45.

[B154014] Blick Theo (2011). Abundant and rare spiders on tree trunks in German forests (Arachnida, Araneae). Arachnologische Mitteilungen.

[B154024] Coddington J A, Levi H W (1991). Systematics and Evolution of Spiders (Araneae). Annual Review of Ecology and Systematics.

[B154201] Coddington Jonathan A., Young Laurel H., Coyle Frederick A. (1996). Estimating spider species richness in a southern Appalachian cove hardwood forest. Journal of Arachnology.

[B154035] Coddington Jonathan A., Agnarsson Ingi, Miller Jeremy A., Kuntner Matjaž, Hormiga Gustavo (2009). Undersampling bias: the null hypothesis for singleton species in tropical arthropod surveys. Journal of Animal Ecology.

[B154049] Dornelas M., Magurran A. E., Buckland S. T., Chao A., Chazdon R. L., Colwell R. K., Curtis T., Gaston K. J., Gotelli N. J., Kosnik M. A., McGill B., McCune J. L., Morlon H., Mumby P. J., Ovreas L., Studeny A., Vellend M. (2013). Quantifying temporal change in biodiversity: challenges and opportunities. Proceedings of the Royal Society B: Biological Sciences.

[B154220] Foelix Rianer F. (2010). Biology of Spiders.

[B154075] Hebert P. D. N., Cywinska A., Ball S. L., deWaard J. R. (2003). Biological identifications through DNA barcodes. Proceedings of the Royal Society B: Biological Sciences.

[B154088] Hoban S., Vernesi C. (2012). Challenges in global biodiversity conservation and solutions that cross sociology, politics, economics and ecology. Biology Letters.

[B154211] Maurer Richard, Hänggi Ambros (1990). Katalog der schweizerischen Spinnen. Documenta Faunistica Helvetiae 12.

[B154099] Scharff Nikolaj, Coddington Jonathan A., Griswold Charles E., Hormiga Gustavo, Bjørn Per de Place (2003). When to quit? Estimating spider species richness in a northern european deciduous forest. Journal of Arachnology.

[B154174] Global Genome Initiative. http://www.mnh.si.edu/ggi/.

[B154229] Ezlab, Evolutionary Zoology Lab. http://ezlab.zrc-sazu.si/.

[B154153] Spiders of Europe. www.araneae.unibe.ch.

[B157321] Checklist of the spiders of Central Europe. (Arachnida: Araneae). Version 1.. http://www.arages.de/checklist.html#2004_Araneae.

[B154113] The World Spider Catalog, Version 13.0.. http://research.amnh.org/iz/spiders/catalog/INTRO1.html.

