# Corrigendum: Targeting a portion of central European spider diversity for permanent preservation

**DOI:** 10.3897/BDJ.3.e4301

**Published:** 2015-01-21

**Authors:** Klemen Čandek, Matjaž Gregorič, Rok Kostanjšek, Holger Frick, Christian Kropf, Matjaž Kuntner

**Affiliations:** † Institute of Biology, Scientific Research Centre, Slovenian Academy of Sciences and Arts, Ljubljana, Slovenia; ‡ Department of Biology, Biotechnical faculty, University of Ljubljana, Ljubljana, Slovenia; § National Collection of Natural History, Office of Environment, Vaduz, Liechtenstein; | Department of Invertebrates, Natural History Museum, Bern, Switzerland; ¶ National Museum of Natural History, Smithsonian Institution, Washington, DC, United States of America

## Main Text

After publication (Čandek et al. 2013), we discovered identification and count errors. We reexamined material and identified five species (*Araniella opisthographa*, *Microlinyphia impigra*, *Oryphantes cf. angulatus*, *Alopecosa taeniata* and *Pardosa cf. saltans*) that are new to the checklist and omitted eight species (*Lathys nielseni*, *Dysdera erythrina*, *Megalepthyphantes nebulosus*, *Meioneta similis*, *Pelecopsis mengei*, *Alopecosa aculeata*, *Pardosa cf. hyperborea* and *Pellenes tripunctatus*) from it. We also added *cf.* to sixteen less reliably identified species. All errors are corrected below in bold letters:

**Abstract:** The sentence with corrected counts should now read: “We here report on the faunistic part of this project, which resulted in **320** species (**224** in Slovenia, **142** in Switzerland) for which identification was reasonably established.”

**Analysis:** The paragraph with corrected counts should now read: “Altogether, we identified **1595** adult individuals, belonging to **320** species, **184** genera and 33 spider families. From the 76 localities in Slovenia, we recorded **224** species belonging to **144** genera and 31 families. From the 33 localities in Switzerland, we recorded **142** species belonging to **89** genera and 18 families. The number of unique species was **178** and **96** for Slovenia and Switzerland, respectively. As an indication of rare species in our survey, singletons (those species represented on the list with a single individual (Coddington et al. 1996, Coddington et al. 2009) represented **36.6%** (**117** species).”

**Checklist**:

Page 10: The entries for *Araniella cucurbitina* (Clerck, 1757) represent that species and *Araniella opisthographa* (Kulczyn'ski, 1905) (new to the checklist) and the corrected text should read:

*Araniella cucurbitina* (Clerck, 1757)

Materials

1. locationID: SI41; country: Slovenia; locality: Socerb, Osp; minimumElevationInMeters: 116; maximumElevationInMeters: 116; decimalLatitude: 45.5819; decimalLongitude: 13.8558; eventDate: 2012-06-07; habitat: trail from Socerb to Osp; sex: 1 female; recordedBy: Kuntner, Gregorič, Čandek, Kralj-Fišer, Cheng
2. locationID: SI50; country: Slovenia; locality: Sp. Prapreče; minimumElevationInMeters: 351; maximumElevationInMeters: 351; decimalLatitude: 46.1620; decimalLongitude: 14.6933; eventDate: 2010-08-03/2012-05-28; habitat: house and surroundings; sex: **1 male**; recordedBy: Kuntner, Čandek

***Araniella opisthographa* (Kulczyn'ski, 1905)**

**Materials**

1. **locationID: SI50; country: Slovenia; locality: Sp. Prapreče; minimumElevationInMeters: 351; maximumElevationInMeters: 351; decimalLatitude: 46.1620; decimalLongitude: 14.6933; eventDate: 2010-08-03/2012-05-28; habitat: house and surroundings; sex: 1 male; recordedBy: Kuntner, Čandek**
2. **locationID: SI58; country: Slovenia; locality: Budanje; minimumElevationInMeters: 243; maximumElevationInMeters: 243; decimalLatitude: 45.8743; decimalLongitude: 13.9497; eventDate: 2011-05-07; habitat: school and surroundings; sex: 1 male; recordedBy: Čandek**

Page 19-20: Due to misidentification, *Lathys nielseni* (Schenkel, 1932) is omitted, and the only female reported is added to the material listed for *Lathys humilis* (Blackwall, 1855) and the corrected text should read:

*Lathys humilis* (Blackwall, 1855)

Materials

1. locationID: SI56; country: Slovenia; locality: Dinaric Karst, Novelo; minimumElevationInMeters: 358; maximumElevationInMeters: 359; decimalLatitude: 45.8533; decimalLongitude: 13.6552; eventDate: 2011-04-04/05-10; habitat: overgrowth; sex: 1 female; recordedBy: Gregorič, Čandek, Kralj-Fišer
2. **locationID: SI41; country: Slovenia; locality: Socerb, Osp; minimumElevationInMeters: 116; maximumElevationInMeters: 116; decimalLatitude: 45.5819; decimalLongitude: 13.8558; eventDate: 2012-06-07; habitat: trail from Socerb to Osp; sex: 1 female; recordedBy: Kuntner, Gregorič, Čandek, Kralj-Fišer, Cheng**

Page 20: Due to misidentification, *Dysdera erythrina* (Walckenaer, 1802) is omitted, and the only female reported is added to the material listed for *Dysdera adriatica* Kulczyn'ski, 1897, and the corrected text should read:

*Dysdera adriatica* Kulczyn'ski, 1897

Materials

1. locationID: SI12; country: Slovenia; locality: Divača; minimumElevationInMeters: 660; maximumElevationInMeters: 660; decimalLatitude: 45.6788; decimalLongitude: 14.0437; eventDate: 2012-07-22; habitat: forest; sex: 1 female, 1 male; recordedBy: Kostanjšek, RTŠB 2012
2. **locationID: SI08; country: Slovenia; locality: Ribnica, Pivka; minimumElevationInMeters: 400; maximumElevationInMeters: 400; decimalLatitude: 45.6333; decimalLongitude: 14.1392; eventDate: 2012-07-21; habitat: forest; sex: 1 female; recordedBy: Kostanjšek, RTŠB 2012**

Page 23: Misspelling: *Nomisia extornata* should read ***Nomisia exornata*.**

Page 35, 41: Due to misidentification, *Megalepthyphantes nebulosus* (Sundevall, 1830) is omitted, and all representatives are added to the material listed for *Mughiphantes mughi* (Fickert, 1875) and the corrected text should read:

*Mughiphantes mughi* (Fickert, 1875)

Materials

1. locationID: CH21; country: Switzerland; locality: Grison Alps, Alp Flix, Salategnas; minimumElevationInMeters: 1970; maximumElevationInMeters: 1970; decimalLatitude: 46.5194; decimalLongitude: 9.6490; eventDate: 2011-07-12; habitat: swamp grazed vegetation; sex: 1 male; recordedBy: Kuntner, Gregorič, Čandek
2. locationID: CH23; country: Switzerland; locality: Grison Alps, Alp Flix, Salategnas; minimumElevationInMeters: 1900; maximumElevationInMeters: 1900; decimalLatitude: 46.5141; decimalLongitude: 9.6448; eventDate: 2011-07-12; habitat: forest opening, grass and shrubs; sex: **6 females**; recordedBy: Kuntner, Gregorič, Čandek
3. locationID: CH24; country: Switzerland; locality: Grison Alps, Alp Flix, Salategnas; minimumElevationInMeters: 1830; maximumElevationInMeters: 1830; decimalLatitude: 46.5131; decimalLongitude: 9.6430; eventDate: 2011-07-12; habitat: meadow and forest; sex: 1 male; recordedBy: Kuntner, Gregorič, Čandek
4. locationID: CH31; country: Switzerland; locality: Grison Alps, Alp Flix - Lai Neir; minimumElevationInMeters: 1910; maximumElevationInMeters: 1910; decimalLatitude: 46.5343; decimalLongitude: 9.6375; eventDate: 2011-07-16; habitat: lake and swamp around forest; sex: 5 females, 2 males; recordedBy: Kuntner, Gregorič, Čandek
5. locationID: CH32; country: Switzerland; locality: Grison Alps, Alp Flix, Salategnas; minimumElevationInMeters: 1955; maximumElevationInMeters: 1955; decimalLatitude: 46.5203; decimalLongitude: 9.6458; eventDate: 2011-07-16; habitat: timberline forest, moss; sex: 3 females, 1 male; recordedBy: Kuntner, Gregorič, Čandek
6. **locationID: CH17; country: Switzerland; locality: Engadin, Bivio; minimumElevationInMeters: 1780; maximumElevationInMeters: 1780; decimalLatitude: 46.4753; decimalLongitude: 9.6469; eventDate: 2011-07-11; habitat: forest and river edge; sex: 1 female; recordedBy: Kuntner, Gregorič, Čandek**

Page 37: ***Meioneta similis* (Kulczyn'ski, 1926)** data should be **deleted**.

Page 38: The entries for *Micrargus alpinus* (O. P.-Cambridge, 1871) represent that species and *Micrargus herbigradus* (Blackwall, 1854) and the corrected text should read:

*Micrargus alpinus* (O. P.-Cambridge, 1871)

Materials

1. locationID: CH28; country: Switzerland; locality: Grison Alps, Alp Flix, Salategnas; minimumElevationInMeters: 1713; maximumElevationInMeters: 1713; decimalLatitude: 46.5165; decimalLongitude: 9.6387; eventDate: 2011-07-15; habitat: forest edge; sex: 1 female; recordedBy: Kuntner, Gregorič, Čandek
2. locationID: CH30; country: Switzerland; locality: Grison Alps, Alp Flix - Lai Flix; minimumElevationInMeters: 1967; maximumElevationInMeters: 1967; decimalLatitude: 46.5358; decimalLongitude: 9.6409; eventDate: 2011-07-16; habitat: next to alpine lake; sex: 1 female; recordedBy: Kuntner, Gregorič, Čandek
3. locationID: CH31; country: Switzerland; locality: Grison Alps, Alp Flix - Lai Neir; minimumElevationInMeters: 1910; maximumElevationInMeters: 1910; decimalLatitude: 46.5343; decimalLongitude: 9.6375; eventDate: 2011-07-16; habitat: lake and swamp around forest; sex: 1 female; recordedBy: Kuntner, Gregorič, Čandek

*Micrargus herbigradus* (Blackwall, 1854)

Materials

1. locationID: SI15; country: Slovenia; locality: Apače; minimumElevationInMeters: 220; maximumElevationInMeters: 220; decimalLatitude: 46.6804; decimalLongitude: 15.8988; eventDate: 2011-07-26; habitat: forest; sex: 1 female; recordedBy: Kostanjšek, RTŠB 2011
2. **locationID: CH03; country: Switzerland; locality: Bernese Alps, Gasteretal; minimumElevationInMeters: 1520; maximumElevationInMeters: 1520; decimalLatitude: 46.4498; decimalLongitude: 7.7135; eventDate: 2011-07-07; habitat: spruce forest; sex: 1 female; recordedBy: Kuntner, Gregorič, Čandek**

Page 40, 50-51: The entries for *Mughiphantes cornutus* (Schenkel, 1927) represent that species and *Tenuiphantes mengei* (Kulczyn'ski, 1887) and the corrected text should read:

*Mughiphantes cornutus* (Schenkel, 1927)

Materials

1. locationID: CH16; country: Switzerland; locality: Engadin, Silvaplana; minimumElevationInMeters: 1930; maximumElevationInMeters: 1930; decimalLatitude: 46.4667; decimalLongitude: 9.7946; eventDate: 2011-07-11; habitat: Larix and Pinus forest; sex: 1 female; recordedBy: Kuntner, Gregorič, Čandek
2. locationID: CH22; country: Switzerland; locality: Grison Alps, Alp Flix, Salategnas; minimumElevationInMeters: 1900; maximumElevationInMeters: 1900; decimalLatitude: 46.5152; decimalLongitude: 9.6466; eventDate: 2011-07-12; habitat: forest ground; sex: 1 female, 1 male; recordedBy: Kuntner, Gregorič, Čandek

*Tenuiphantes mengei* (Kulczyn'ski, 1887)

Materials

1. locationID: CH01; country: Switzerland; locality: Bernese Alps, Gasteretal; minimumElevationInMeters: 1662; maximumElevationInMeters: 1662; decimalLatitude: 46.4457; decimalLongitude: 7.7413; eventDate: 2011-07-07; habitat: alpine meadow; sex: 4 females; recordedBy: Kuntner, Gregorič, Čandek
2. locationID: CH02; country: Switzerland; locality: Bernese Alps, Gasteretal; minimumElevationInMeters: 1698; maximumElevationInMeters: 1698; decimalLatitude: 46.4486; decimalLongitude: 7.7438; eventDate: 2011-07-07; habitat: spruce thicket and grass; sex: 14 females, 7 males; recordedBy: Kuntner, Gregorič, Čandek
3. locationID: CH05; country: Switzerland; locality: Bernese Alps, Gasteretal; minimumElevationInMeters: 1380; maximumElevationInMeters: 1380; decimalLatitude: 46.4674; decimalLongitude: 7.6640; eventDate: 2011-07-07; habitat: river vegetation; sex: 1 female; recordedBy: Kuntner, Gregorič, Čandek
4. locationID: CH06; country: Switzerland; locality: Bernese Alps, Kandersteg; minimumElevationInMeters: 1677; maximumElevationInMeters: 1677; decimalLatitude: 46.5020; decimalLongitude: 7.6992; eventDate: 2011-07-07; habitat: alpine meadow; sex: 3 females, 2 males; recordedBy: Kuntner, Gregorič, Čandek
5. locationID: CH09; country: Switzerland; locality: Pennine Alps, Mattertal; minimumElevationInMeters: 1447; maximumElevationInMeters: 1447; decimalLatitude: 46.0976; decimalLongitude: 7.7789; eventDate: 2011-07-08; habitat: forest and meadow near river; sex: 5 females, 1 male; recordedBy: Kuntner, Gregorič, Čandek
6. locationID: CH12; country: Switzerland; locality: Bernese Alps, Nessental; minimumElevationInMeters: 930; maximumElevationInMeters: 930; decimalLatitude: 46.7213; decimalLongitude: 8.3039; eventDate: 2011-07-10; habitat: grassland and lone trees; sex: 1 female, 1 male; recordedBy: Kuntner, Gregorič, Čandek
7. locationID: CH13; country: Switzerland; locality: Bernese Alps, Sustenpass; minimumElevationInMeters: 2040; maximumElevationInMeters: 2040; decimalLatitude: 46.7330; decimalLongitude: 8.4324; eventDate: 2011-07-10; habitat: alpine grassland and shrubs; sex: 4 females; recordedBy: Kuntner, Gregorič, Čandek
8. locationID: CH16; country: Switzerland; locality: Engadin, Silvaplana; minimumElevationInMeters: 1930; maximumElevationInMeters: 1930; decimalLatitude: 46.4667; decimalLongitude: 9.7946; eventDate: 2011-07-11; habitat: Larix and Pinus forest; sex: 1 female; recordedBy: Kuntner, Gregorič, Čandek
9. locationID: CH17; country: Switzerland; locality: Engadin, Bivio; minimumElevationInMeters: 1780; maximumElevationInMeters: 1780; decimalLatitude: 46.4753; decimalLongitude: 9.6469; eventDate: 2011-07-11; habitat: forest and river edge; sex: 1 male; recordedBy: Kuntner, Gregorič, Čandek
10. locationID: CH23; country: Switzerland; locality: Grison Alps, Alp Flix, Salategnas; minimumElevationInMeters: 1900; maximumElevationInMeters: 1900; decimalLatitude: 46.5141; decimalLongitude: 9.6448; eventDate: 2011-07-12; habitat: forest opening, grass and shrubs; sex: 2 females; recordedBy: Kuntner, Gregorič, Čandek
11. locationID: CH24; country: Switzerland; locality: Grison Alps, Alp Flix, Salategnas; minimumElevationInMeters: 1830; maximumElevationInMeters: 1830; decimalLatitude: 46.5131; decimalLongitude: 9.6430; eventDate: 2011-07-12; habitat: meadow and forest; sex: 1 female, 3 males; recordedBy: Kuntner, Gregorič, Čandek
12. locationID: CH25; country: Switzerland; locality: Grison Alps, Alp Flix, Salategnas; minimumElevationInMeters: 1950; maximumElevationInMeters: 1950; decimalLatitude: 46.5159; decimalLongitude: 9.6496; eventDate: 2011-07-12/16; habitat: meadow and shrubs at stream; sex: 1 female, 1 male; recordedBy: Kuntner, Gregorič, Čandek
13. locationID: CH28; country: Switzerland; locality: Grison Alps, Alp Flix, Salategnas; minimumElevationInMeters: 1713; maximumElevationInMeters: 1713; decimalLatitude: 46.5165; decimalLongitude: 9.6387; eventDate: 2011-07-15; habitat: forest edge; sex: 3 females, 3 males; recordedBy: Kuntner, Gregorič, Čandek
14. locationID: CH30; country: Switzerland; locality: Grison Alps, Alp Flix - Lai Flix; minimumElevationInMeters: 1967; maximumElevationInMeters: 1967; decimalLatitude: 46.5358; decimalLongitude: 9.6409; eventDate: 2011-07-16; habitat: next to alpine lake; sex: 4 females, 2 males; recordedBy: Kuntner, Gregorič, Čandek
15. locationID: CH31; country: Switzerland; locality: Grison Alps, Alp Flix - Lai Neir; minimumElevationInMeters: 1910; maximumElevationInMeters: 1910; decimalLatitude: 46.5343; decimalLongitude: 9.6375; eventDate: 2011-07-16; habitat: lake and swamp around forest; sex: 2 females, 1 male; recordedBy: Kuntner, Gregorič, Čandek
16. **locationID: CH32; country: Switzerland; locality: Grison Alps, Alp Flix, Salategnas; **minimumElevationInMeters: 1955; maximumElevationInMeters: 1955; decimalLatitude: **46.5203; decimalLongitude: 9.6458; eventDate: 2011-07-16; habitat: timberline forest, **moss; sex: 1 male; recordedBy: Kuntner, Gregorič, Čandek********

Page 42, 43: The entries for *Neriene clathrata* (Sundevall, 1830) and *Neriene peltata* (Wider, 1834) represent these species and *Microlinyphia impigra* (O. P.-Cambridge, 1871) (new to the checklist) and the corrected text should read:

*Neriene clathrata* (Sundevall, 1830)

Materials

1. locationID: CH09; country: Switzerland; locality: Pennine Alps, Mattertal; minimumElevationInMeters: 1447; maximumElevationInMeters: 1447; decimalLatitude: 46.0976; decimalLongitude: 7.7789; eventDate: 2011-07-08; habitat: forest and meadow near river; sex: 1 female; recordedBy: Kuntner, Gregorič, Čandek
2. locationID: CH15; country: Switzerland; locality: Glarus Alps, near Affeier; minimumElevationInMeters: 817; maximumElevationInMeters: 817; decimalLatitude: 46.7606; decimalLongitude: 9.0933; eventDate: 2011-07-10; habitat: meadow and forest; sex: 1 female; recordedBy: Kuntner, Gregorič, Čandek
3. locationID: SI14; country: Slovenia; locality: Spodnji Velovlek; minimumElevationInMeters: 225; maximumElevationInMeters: 225; decimalLatitude: 46.4768; decimalLongitude: 15.9316; eventDate: 2011-07-25; habitat: forest; sex: 1 female; recordedBy: Kostanjšek, RTŠB 2011
4. locationID: SI56; country: Slovenia; locality: Dinaric Karst, Novelo; minimumElevationInMeters: 358; maximumElevationInMeters: 359; decimalLatitude: 45.8533; decimalLongitude: 13.6552; eventDate: 2011-04-04/05-10; habitat: overgrowth; sex: 1 female, 1 male; recordedBy: Gregorič, Čandek, Kralj-Fišer

*Neriene peltata* (Wider, 1834)

Material

1. locationID: CH02; country: Switzerland; locality: Bernese Alps, Gasteretal; minimumElevationInMeters: 1698; maximumElevationInMeters: 1698; decimalLatitude: 46.4486; decimalLongitude: 7.7438; eventDate: 2011-07-07; habitat: spruce thicket and grass; sex: **1 female**; recordedBy: Kuntner, Gregorič, Čandek

***Microlinyphia impigra* (O. P.-Cambridge, 1871)**

**Materials**

1. **locationID: CH02; country: Switzerland; locality: Bernese Alps, Gasteretal; **minimumElevationInMeters: 1698; maximumElevationInMeters: 1698; decimalLatitude: **46.4486; decimalLongitude: 7.7438; eventDate: 2011-07-07; habitat: spruce thicket and **grass; sex: 1 females; recordedBy: Kuntner, Gregorič, Čandek********
2. **locationID: CH23; country: Switzerland; locality: Grison Alps, Alp Flix, Salategnas; **minimumElevationInMeters: 1900; maximumElevationInMeters: 1900; decimalLatitude: **46.5141; decimalLongitude: 9.6448; eventDate: 2011-07-12; habitat: forest opening, **grass and shrubs; sex: 1 male; recordedBy: Kuntner, Gregorič, Čandek********

Page 44: The entries for *Palliduphantes pallidus* (O. P.-Cambridge, 1871) represent that species and probably *Oryphantes angulatus* (O. P.-Cambridge, 1881) (new to the checklist) and the corrected text should read:

*Palliduphantes pallidus* (O. P.-Cambridge, 1871)

Material

1. locationID: CH03; country: Switzerland; locality: Bernese Alps, Gasteretal; minimumElevationInMeters: 1520; maximumElevationInMeters: 1520; decimalLatitude: 46.4498; decimalLongitude: 7.7135; eventDate: 2011-07-07; habitat: spruce forest; sex: 1 male; recordedBy: Kuntner, Gregorič, Čandek

***Oryphantes cf. angulatus* (O. P.-Cambridge, 1881)**

**Material**

1. **locationID: CH28; country: Switzerland; locality: Grison Alps, Alp Flix, Salategnas; **minimumElevationInMeters: 1713; maximumElevationInMeters: 1713; decimalLatitude: **46.5165; decimalLongitude: 9.6387; eventDate: 2011-07-15; habitat: forest edge; sex: 1 **female; recordedBy: Kuntner, Gregorič, Čandek********

Page 44: *Oedothorax gibbifer* (Kulczyn'ski, 1882) is represented with one male and the corrected text should read:

*Oedothorax gibbifer* (Kulczyn'ski, 1882)

Material

1. locationID: CH25; country: Switzerland; locality: Grison Alps, Alp Flix, Salategnas; minimumElevationInMeters: 1950; maximumElevationInMeters: 1950; decimalLatitude: 46.5159; decimalLongitude: 9.6496; eventDate: 2011-07-12/16; habitat: meadow and shrubs at stream; sex: **1 male**; recordedBy: Kuntner, Gregorič, Čandek

﻿Page 27, 45: Due to misidentification, *Pelecopsis mengei* (Simon, 1884) is omitted, and all representatives reported are added to the material listed for *Ceratinella brevipes* (Westring, 1851) and the corrected text should read:

*Ceratinella brevipes* (Westring, 1851)

Materials

1. locationID: CH32; country: Switzerland; locality: Grison Alps, Alp Flix, Salategnas; minimumElevationInMeters: 1955; maximumElevationInMeters: 1955; decimalLatitude: 46.5203; decimalLongitude: 9.6458; eventDate: 2011-07-16; habitat: timberline forest, moss; sex: 1 male; recordedBy: Kuntner, Gregorič, Čandek
2. **locationID: CH22; country: Switzerland; locality: Grison Alps, Alp Flix, Salategnas; **minimumElevationInMeters: 1900; maximumElevationInMeters: 1900; decimalLatitude: **46.5152; decimalLongitude: 9.6466; eventDate: 2011-07-12; habitat: forest ground; sex:1 **female; recordedBy: Kuntner, Gregorič, Čandek********
3. **locationID: CH24; country: Switzerland; locality: Grison Alps, Alp Flix, Salategnas; **minimumElevationInMeters: 1830; maximumElevationInMeters: 1830; decimalLatitude: **46.5131; decimalLongitude: 9.6430; eventDate: 2011-07-12; habitat: meadow and forest; **sex: 1 female; recordedBy: Kuntner, Gregorič, Čandek********

Page 46: *Pocadicnemis pumila* probably represent that species. The correct number of individuals in **material c** is **one female** and the corrected text in material c should read:

*Pocadicnemis cf. pumila* (Blackwall, 1841)

Materials

1. locationID: CH11; country: Switzerland; locality: Bernese Alps, Lake Brienz; minimumElevationInMeters: 600; maximumElevationInMeters: 600; decimalLatitude: 46.7569; decimalLongitude: 8.0107; eventDate: 2011-07-10; habitat: meadows and forest; sex: **1 female**; recordedBy: Kuntner, Gregorič, Čandek

Page 49: The entries for *Tenuiphantes jacksonoides* (van Helsdingen, 1977) probably represent that species and *Tenuiphantes jacksoni* (Schenkel, 1925) and the corrected text should read:

*Tenuiphantes cf. jacksoni* (Schenkel, 1925)

Materials

1. locationID: CH21; country: Switzerland; locality: Grison Alps, Alp Flix, Salategnas; minimumElevationInMeters: 1970; maximumElevationInMeters: 1970; decimalLatitude: 46.5194; decimalLongitude: 9.6490; eventDate: 2011-07-12; habitat: swamp grazed vegetation; sex: 3 females, 1 male; recordedBy: Kuntner, Gregorič, Čandek
2. **locationID: CH28; country: Switzerland; locality: Grison Alps, Alp Flix, Salategnas; **minimumElevationInMeters: 1713; maximumElevationInMeters: 1713; decimalLatitude: **46.5165; decimalLongitude: 9.6387; eventDate: 2011-07-15; habitat: forest edge; sex: 1 **female; recordedBy: Kuntner, Gregorič, Čandek********

*Tenuiphantes cf. jacksonoides* (van Helsdingen, 1977)

Materials

1. locationID: CH12; country: Switzerland; locality: Bernese Alps, Nessental; minimumElevationInMeters: 930; maximumElevationInMeters: 930; decimalLatitude: 46.7213; decimalLongitude: 8.3039; eventDate: 2011-07-10; habitat: grassland and lone trees; sex: 1 male; recordedBy: Kuntner, Gregorič, Čandek
2. locationID: CH22; country: Switzerland; locality: Grison Alps, Alp Flix, Salategnas; minimumElevationInMeters: 1900; maximumElevationInMeters: 1900; decimalLatitude: 46.5152; decimalLongitude: 9.6466; eventDate: 2011-07-12; habitat: forest ground; sex: 1 female, 4 males; recordedBy: Kuntner, Gregorič, Čandek
3. locationID: CH23; country: Switzerland; locality: Grison Alps, Alp Flix, Salategnas; minimumElevationInMeters: 1900; maximumElevationInMeters: 1900; decimalLatitude: 46.5141; decimalLongitude: 9.6448; eventDate: 2011-07-12; habitat: forest opening, grass and shrubs; sex: 4 females, 3 males; recordedBy: Kuntner, Gregorič, Čandek
4. locationID: CH28; country: Switzerland; locality: Grison Alps, Alp Flix, Salategnas; minimumElevationInMeters: 1713; maximumElevationInMeters: 1713; decimalLatitude: 46.5165; decimalLongitude: 9.6387; eventDate: 2011-07-15; habitat: forest edge; sex: **6 females**; recordedBy: Kuntner, Gregorič, Čandek

Page 54: Due to misidentification, *Alopecosa aculeata* (Clerck, 1757) is omitted, and the only female reported represents the species *Alopecosa taeniata* (C. L. Koch, 1835) (new to the checklist) and the corrected text should read:

***Alopecosa taeniata* (C. L. Koch, 1835)**

**Material**

1. **locationID: CH23; country: Switzerland; locality: Grison Alps, Alp Flix, Salategnas; **minimumElevationInMeters: 1900; maximumElevationInMeters: 1900; decimalLatitude: **46.5141; decimalLongitude: 9.6448; eventDate: 2011-07-12; habitat: forest opening, **grass and shrubs; sex: 1 female; recordedBy: Kuntner, Gregorič, Čandek********

Page 58-59: Due to misidentification, *Pardosa cf. hyperborea* (Thorell, 1872) is omitted, and the only female reported is added to the material listed for *Pardosa oreophila* Simon, 1937 and the corrected text should read:

*Pardosa oreophila* Simon, 1937

Materials

1. locationID: CH10; country: Switzerland; locality: Bernese Alps, Kleine Scheidegg; minimumElevationInMeters: 2061; maximumElevationInMeters: 2061; decimalLatitude: 46.5853; decimalLongitude: 7.9606; eventDate: 2011-07-09; habitat: grassland; sex: 1 female; recordedBy: Kuntner, Gregorič, Čandek
2. locationID: CH13; country: Switzerland; locality: Bernese Alps, Sustenpass; minimumElevationInMeters: 2040; maximumElevationInMeters: 2040; decimalLatitude: 46.7330; decimalLongitude: 8.4324; eventDate: 2011-07-10; habitat: alpine grassland and shrubs; sex: 4 males; recordedBy: Kuntner, Gregorič, Čandek
3. **locationID: CH14; country: Switzerland; locality: Glarus Alps, Oberalppass; **minimumElevationInMeters: 2040; maximumElevationInMeters: 2040; decimalLatitude: **46.6617; decimalLongitude: 8.6719; eventDate: 2011-07-10; habitat: grassland and **shrubs; sex: 1 female; recordedBy: Kuntner, Gregorič, Čandek********

Page 58-59: The entries for *Pardosa cf. lugubris* (Walckenaer, 1802) represent that species and *Pardosa cf. saltans* Töpfer-Hofmann, 2000 (new to the checklist) and the corrected text should read:

*Pardosa cf. lugubris* (Walckenaer, 1802)

Materials

1. locationID: SI15; country: Slovenia; locality: Apače; minimumElevationInMeters: 220; maximumElevationInMeters: 220; decimalLatitude: 46.6804; decimalLongitude: 15.8988; eventDate: 2011-07-26; habitat: forest; sex: 1 female; recordedBy: Kostanjšek, RTŠB 2011
2. locationID: SI38; country: Slovenia; locality: Poreče; minimumElevationInMeters: 135; maximumElevationInMeters: 135; decimalLatitude: 45.8188; decimalLongitude: 13.9692; eventDate: 2011-05-08; habitat: grassland; sex: 1 female; recordedBy: Čandek
3. locationID: SI40; country: Slovenia; locality: Slavnik; minimumElevationInMeters: 816; maximumElevationInMeters: 816; decimalLatitude: 45.5499; decimalLongitude: 13.9619; eventDate: 2010-08-26; habitat: grassland and forest; sex: 3 females; recordedBy: Kuntner, Lokovšek
4. locationID: SI58; country: Slovenia; locality: Budanje; minimumElevationInMeters: 243; maximumElevationInMeters: 243; decimalLatitude: 45.8743; decimalLongitude: 13.9497; eventDate: 2011-05-07; habitat: school and surroundings; sex: 2 females; recordedBy: Čandek
5. locationID: SI59; country: Slovenia; locality: Budanje; minimumElevationInMeters: 305; maximumElevationInMeters: 305; decimalLatitude: 45.8797; decimalLongitude: 13.9468; eventDate: 2011-05-07; habitat: forest; sex: 3 females, 4 males; recordedBy: Čandek
6. locationID: SI60; country: Slovenia; locality: Budanje; minimumElevationInMeters: 295; maximumElevationInMeters: 295; decimalLatitude: 45.8799; decimalLongitude: 13.9459; eventDate: 2011-05-07; habitat: forest clearing; sex: 2 females; recordedBy: Čandek
7. locationID: SI61; country: Slovenia; locality: Sekirišče; minimumElevationInMeters: 750; maximumElevationInMeters: 750; decimalLatitude: 45.8631; decimalLongitude: 14.5367; eventDate: 2011-06-23/2012-06-21; habitat: house, grassland, overgrowth; sex: 4 females; recordedBy: Čandek

***Pardosa cf. saltans* Töpfer-Hofmann, 2000**

**Materials**

1. **locationID: CH11; country: Switzerland; locality: Bernese Alps, Lake Brienz; minimumElevationInMeters: 600; maximumElevationInMeters: 600; decimalLatitude: 46.7569; decimalLongitude: 8.0107; eventDate: 2011-07-10; habitat: meadows and forest; sex: 3 females; recordedBy: Kuntner, Gregorič, Čandek**
2. **locationID: CH12; country: Switzerland; locality: Bernese Alps, Nessental; **minimumElevationInMeters: 930; maximumElevationInMeters: 930; decimalLatitude: **46.7213; decimalLongitude: 8.3039; eventDate: 2011-07-10; habitat: grassland and lone **trees; sex: 2 females; recordedBy: Kuntner, Gregorič, Čandek********
3. **locationID: CH15; country: Switzerland; locality: Glarus Alps, near Affeier; **minimumElevationInMeters: 817; maximumElevationInMeters: 817; decimalLatitude: **46.7606; decimalLongitude: 9.0933; eventDate: 2011-07-10; habitat: meadow and forest; **sex: 4 females; recordedBy: Kuntner, Gregorič, Čandek********
4. **locationID: CH27; country: Switzerland; locality: Grison Alps, road to Davos; **minimumElevationInMeters: 1180; maximumElevationInMeters: 1180; decimalLatitude: 46.6808; decimalLongitude: 9.6557; eventDate: 2011-07-15; habitat: roadside vegetation and forest edge; sex: 6 females, 1 male; recordedBy: Kuntner, Gregorič, Čandek****

Page 69-70: The entries for *Evarcha laetabunda* (C. L. Koch, 1846) represent that species and *Evarcha michailovi* Logunov, 1992 and the corrected text should read:

*Evarcha laetabunda* (C. L. Koch, 1846)

Materials

1. locationID: SI38; country: Slovenia; locality: Poreče; minimumElevationInMeters: 135; maximumElevationInMeters: 135; decimalLatitude: 45.8188; decimalLongitude: 13.9692; eventDate: 2011-05-08; habitat: grassland; sex: 2 females; recordedBy: Čandek
2. locationID: SI52; country: Slovenia; locality: Dinaric Karst, Griže; minimumElevationInMeters: 484; maximumElevationInMeters: 484; decimalLatitude: 45.7506; decimalLongitude: 13.9509; eventDate: 2011-04-04/05-10; habitat: overgrowth; sex: 1 male; recordedBy: Gregorič, Čandek, Kralj-Fišer
3. locationID: SI53; country: Slovenia; locality: Dinaric Karst, Griže; minimumElevationInMeters: 434; maximumElevationInMeters: 434; decimalLatitude: 45.7548; decimalLongitude: 13.9495; eventDate: 2011-05-10/2011-06-21; habitat: grassland; sex: **1 female**; recordedBy: Gregorič, Čandek

*Evarcha michailovi* Logunov, 1992

Materials

1. locationID: SI52; country: Slovenia; locality: Dinaric Karst, Griže; minimumElevationInMeters: 484; maximumElevationInMeters: 484; decimalLatitude: 45.7506; decimalLongitude: 13.9509; eventDate: 2011-04-04/05-10; habitat: overgrowth; sex: 1 male; recordedBy: Gregorič, Čandek, Kralj-Fišer
2. **locationID: SI56; country: Slovenia; locality: Dinaric Karst, Novelo; **minimumElevationInMeters: 358; maximumElevationInMeters: 359; decimalLatitude: **45.8533; decimalLongitude: 13.6552; eventDate: 2011-04-04/05-10; habitat: overgrowth; **sex: 5 females, 2 males; recordedBy: Gregorič, Čandek, Kralj-Fišer********
3. **locationID: SI53; country: Slovenia; locality: Dinaric Karst, Griže; **minimumElevationInMeters: 434; maximumElevationInMeters: 434; decimalLatitude: **45.7548; decimalLongitude: 13.9495; eventDate: 2011-05-10/2011-06-21; habitat: **grassland; sex: 1 female; recordedBy: Gregorič, Čandek********

Page 72: Misspelling: *Leptorchetes* should read ***Leptorchestes***

Page 74: Due to misidentification, *Pellenes tripunctatus* (Walckenaer, 1802) is omitted, and the only male reported is added to the material listed for *Pellenes seriatus* (Thorell, 1875) and the corrected text should read:

*Pellenes seriatus* (Thorell, 1875)

Materials

1. locationID: SI38; country: Slovenia; locality: Poreče; minimumElevationInMeters: 135; maximumElevationInMeters: 135; decimalLatitude: 45.8188; decimalLongitude: 13.9692; eventDate: 2011-05-08; habitat: grassland; sex: 1 female; recordedBy: Čandek
2. locationID: SI52; country: Slovenia; locality: Dinaric Karst, Griže; minimumElevationInMeters: 484; maximumElevationInMeters: 484; decimalLatitude: 45.7506; decimalLongitude: 13.9509; eventDate: 2011-04-04/05-10; habitat: overgrowth; sex: 1 male; recordedBy: Gregorič, Čandek, Kralj-Fišer
3. locationID: SI53; country: Slovenia; locality: Dinaric Karst, Griže; minimumElevationInMeters: 434; maximumElevationInMeters: 434; decimalLatitude: 45.7548; decimalLongitude: 13.9495; eventDate: 2011-05-10/2011-06-21; habitat: grassland; sex: 1 female; recordedBy: Gregorič, Čandek
4. locationID: SI55; country: Slovenia; locality: Dinaric Karst, Lokvice; minimumElevationInMeters: 273; maximumElevationInMeters: 275; decimalLatitude: 45.8659; decimalLongitude: 13.6102; eventDate: 2011-04-04/05-10; habitat: overgrowth; sex: 1 female; recordedBy: Gregorič, Čandek, Kralj-Fišer
5. **locationID: SI56; country: Slovenia; locality: Dinaric Karst, Novelo; **minimumElevationInMeters: 358; maximumElevationInMeters: 359; decimalLatitude: **45.8533; decimalLongitude: 13.6552; eventDate: 2011-04-04/05-10; habitat: overgrowth; **sex: 1 male; recordedBy: Gregorič, Čandek, Kralj-Fišer********

Page 78: The entries for *Metellina merianae* (Scopoli, 1763) represent that species and *Metellina segmentata* (Clerk, 1757) and the corrected text should read:

*Metellina merianae* (Scopoli, 1763)

Materials

1. locationID: CH02; country: Switzerland; locality: Bernese Alps, Gasteretal; minimumElevationInMeters: 1698; maximumElevationInMeters: 1698; decimalLatitude: 46.4486; decimalLongitude: 7.7438; eventDate: 2011-07-07; habitat: spruce thicket and grass; sex: 2 females; recordedBy: Kuntner, Gregorič, Čandek
2. locationID: SI01; country: Slovenia; locality: Biš; minimumElevationInMeters: 225; maximumElevationInMeters: 225; decimalLatitude: 46.5374; decimalLongitude: 15.8963; eventDate: 2011-07-22; habitat: forest; sex: 1 female; recordedBy: Kostanjšek, RTŠB
3. locationID: SI29; country: Slovenia; locality: Gradišče pri Lukovici, Gradiško jezero; minimumElevationInMeters: 360; maximumElevationInMeters: 360; decimalLatitude: 46.1626; decimalLongitude: 14.7127; eventDate: 2011-10-06; habitat: lake edge; sex: 2 females, 1 male; recordedBy: Čandek

***Metellina segmentata* (Clerk, 1757)**

**Materials**

1. **locationID: SI36; country: Slovenia; locality: Močilnik; minimumElevationInMeters: 318; **maximumElevationInMeters: 318; decimalLatitude: 45.9547; decimalLongitude: 14.2925; **eventDate: 2011-10-02; habitat: forest edge; sex: 1 male; recordedBy: Kuntner******
2. **locationID: SI50; country: Slovenia; locality: Sp. Prapreče; minimumElevationInMeters: **351; maximumElevationInMeters: 351; decimalLatitude: 46.1620; decimalLongitude: **14.6933; eventDate: 2010-08-03/2012-05-28; habitat: house and surroundings; sex: 1 **female, 1 male; recordedBy: Kuntner, Čandek********

Page 86: The entries for *Robertus lividus* (Blackwall, 1836) represent that species and *Robertus mediterraneus* Eskov, 1987 and the corrected text should read:

*Robertus lividus* (Blackwall, 1836)

Material

1. locationID: CH24; country: Switzerland; locality: Grison Alps, Alp Flix, Salategnas; minimumElevationInMeters: 1830; maximumElevationInMeters: 1830; decimalLatitude: 46.5131; decimalLongitude: 9.6430; eventDate: 2011-07-12; habitat: meadow and forest; sex: 1 female; recordedBy: Kuntner, Gregorič, Čandek

*Robertus mediterraneus* Eskov, 1987

Materials

1. locationID: CH15; country: Switzerland; locality: Glarus Alps, near Affeier; minimumElevationInMeters: 817; maximumElevationInMeters: 817; decimalLatitude: 46.7606; decimalLongitude: 9.0933; eventDate: 2011-07-10; habitat: meadow and forest; sex: 1 male; recordedBy: Kuntner, Gregorič, Čandek
2. **locationID: CH09; country: Switzerland; locality: Pennine Alps, Mattertal; **minimumElevationInMeters: 1447; maximumElevationInMeters: 1447; decimalLatitude: **46.0976; decimalLongitude: 7.7789; eventDate: 2011-07-08; habitat: forest and meadow **near river; sex: 1 male; recordedBy: Kuntner, Gregorič, Čandek​********

Page 96: *Uloborus walckenaerius* Latreille, 1806 is represented with one female and the corrected text should read:

Material

1. locationID: SI55; country: Slovenia; locality: Dinaric Karst, Lokvice; minimumElevationInMeters: 273; maximumElevationInMeters: 275; decimalLatitude: 45.8659; decimalLongitude: 13.6102; eventDate: 2011-04-04/05-10; habitat: overgrowth; **sex: 1 female**; recordedBy: Gregorič, Čandek, Kralj-Fišer

Due to less reliable identification the following species should read as corrected:

Page 25: *Bolyphantes alticeps* (Sundevall, 1833) should read *Bolyphantes cf. alticeps* (Sundevall, 1833)

Page 26: *Bolyphantes luteolus* (Blackwall, 1833) should read *Bolyphantes cf. luteolus* (Blackwall, 1833)

Page 29: *Erigone svenssoni* Holm, 1975 should read *Erigone cf. svenssoni* Holm, 1975

Page 35: *Meioneta affinis* (Kulczyn'ski, 1898) should read *Meioneta cf. affinis* (Kulczyn'ski, 1898)

Page 35: *Meioneta alpica* (Tanasevitch, 2000) should read *Meioneta cf. alpica* (Tanasevitch, 2000)

Page 35: *Meioneta fuscipalpa* (C. L. Koch, 1836) should read *Meioneta cf. fuscipalpa* (C. L. Koch, 1836)

Page 36: *Meioneta gulosa* (L. Koch, 1869) should read *Meioneta cf. gulosa* (L. Koch, 1869)

Page 36: *Meioneta orites* (Thorell, 1875) should read *Meioneta cf. orites* (Thorell, 1875)

Page 36: *Meioneta rurestris* (C. L. Koch, 1836) should read *Meioneta cf. rurestris* (C. L. Koch, 1836)

Page 37: *Meioneta saxatilis* (Blackwall, 1844) should read *Meioneta cf. saxatilis* (Blackwall, 1844)

Page 37: *Meioneta simplicitarsis* (Simon, 1884) should read *Meioneta cf. simplicitarsis* (Simon, 1884)

Page 46: *Pocadicnemis pumila* (Blackwall, 1841) should read *Pocadicnemis cf. pumila* (Blackwall, 1841)

Page 46: *Pocadicnemis juncea* Locket & Millidge, 1953 should read *Pocadicnemis cf. juncea* Locket & Millidge, 1953

Page 49: *Tenuiphantes jacksoni* (Schenkel, 1925) should read *Tenuiphantes cf. jacksoni* (Schenkel, 1925)

Page 49: *Tenuiphantes jacksonoides* (van Helsdingen, 1977) should read *Tenuiphantes cf. jacksonoides* (van Helsdingen, 1977)

New page: *Oryphantes angulatus* (O. P.-Cambridge, 1881) should read *Oryphantes cf. angulatus* (O. P.-Cambridge, 1881)

## Supplementary Material

Supplementary material 1 Corrected TaxPub XML of the original publication Data type: XML
Brief description: Corrected TaXpub XML of the original publication
File: oo_37244.xml Jeremy Miller

Supplementary material 2 Corrected occurrences as Darwin Core Archive Data type: occurences
File: oo_37245.zip Klemen Čandek, Matjaž Gregorič, Rok Kostanjšek, Holger Frick, Christian Kropf, Matjaž Kuntner
